# Advanced WBG power semiconductor packaging: nanomaterials and nanotechnologies for high-performance die attach paste

**DOI:** 10.1186/s40580-025-00503-3

**Published:** 2025-07-23

**Authors:** Young-Min Ju, Tae-Wan Kim, Seung-Hyun Lee, Ho-Jin Lee, Jinho Ahn, Hak-Sung Kim

**Affiliations:** 1 Department of Mechanical Engineering, Hanyang University, 222, Wangsimni-ro, Seongdong-gu, Seoul, 04763 Republic of Korea; 2 Division of Materials Science and Engineering, Hanyang University, 222, Wangsimni-ro, Seongdong-gu, Seoul, 04763 Republic of Korea; 3 Hanyang Institute of Smart Semiconductor, Hanyang University, 222, Wangsimni-ro, Seongdong-gu, Seoul, Republic of Korea; 4 Hanyang Research Center for Advanced Semiconductor Packaging, Hanyang University, 222, Wangsimni-ro, Seongdong-gu, Seoul, 04763 Republic of Korea; 5 Department of Mechanical Engineering, Hanyang University, 17 Haengdang-Dong, Seongdong-gu, Seoul, 133-791 Republic of Korea; 6 Hanyang Research Center for Advanced Semiconductor Packaging, Hanyang University, 17 Haengdang-Dong, Seongdong-gu, Seoul, 133-791 Republic of Korea

**Keywords:** Wideband gap semiconductor package, Reliability, Nanomaterial, Die attach technology

## Abstract

Wide bandgap (WBG) power semiconductors have attracted significant attention from both academia and industry because they are superior to conventional silicon-based devices. In WBG power semiconductor packages, die attach materials play a crucial role in maximizing device performance and reliability. The die attach interfaces in WBG packages must withstand high operating temperatures (200–300 °C), fast switching frequencies, and great power densities while maintaining excellent thermomechanical reliability. Traditional die attach materials have significant limitations when applied to WBG devices, which has led to intensive research into nanomaterial-based alternatives during the past decade. This review summarizes current state-of-the-art nano-enabled die attach technologies: nanocomposite solders, nano-sintering approaches, and novel nanomaterial formulations specifically engineered for WBG power semiconductor packages. We examine the fundamental mechanisms behind the performance of nanomaterial die attach solutions and their ability to address the thermal management challenges of WBG devices. Furthermore, we examine the reliability of these materials in extreme operating conditions by evaluating their thermal cycling performance, shear strength stability, and microstructural evolution.

**Graphical abstract:**

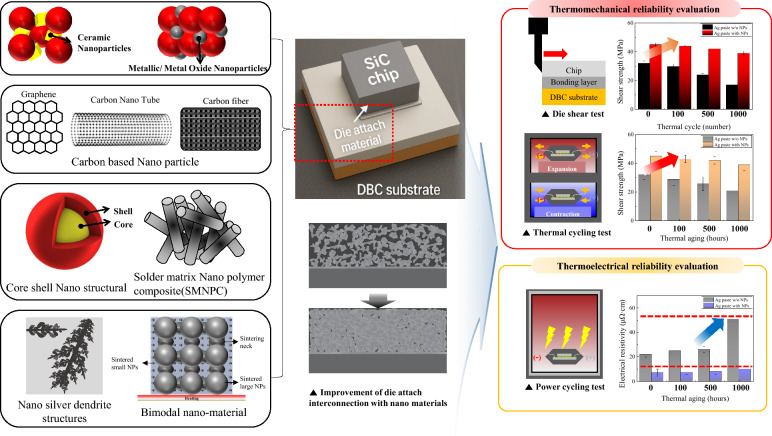

## Introduction

**Fig. 1:**
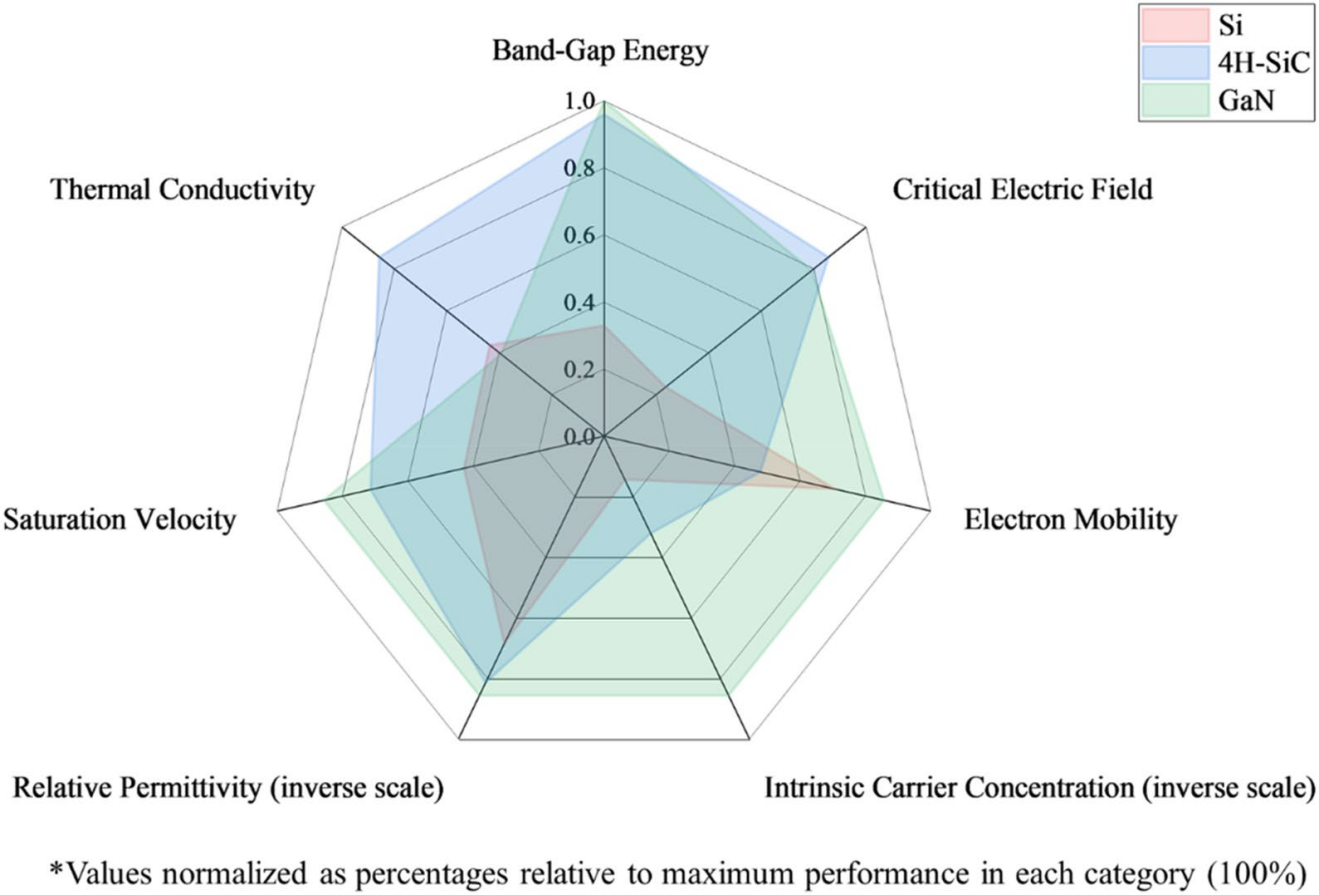
Comparison of power semiconductor materials (Si, 4H-SiC, and GaN) normalized as percentages relative to the maximum performance in each category (100%) [Data based on [10]]

The rapid evolution of power electronic systems has driven a demand for semiconductor devices that can operate at high voltages, frequencies, and temperatures while maintaining excellent efficiency [1–6]. Traditional silicon-based power devices have approached their theoretical limits in terms of power handling capability, switching speed, and thermal performance. This fundamental limitation has led to the emergence of wide bandgap (WBG) semiconductors, particularly silicon carbide (SiC) and gallium nitride (GaN), as promising alternatives for next-generation power electronic applications. WBG semiconductors have excellent material properties compared to silicon-based devices. These include higher breakdown field strength, wider bandgap energy, and better thermal conductivity. Such properties enable significant improvements in device performance and system efficiency [7–9]. As shown in Fig. 1, WBG semiconductors have substantial advantages over silicon in terms of electrical properties. Baliga's figure of merit, a critical indicator of power device performance, reveals that SiC and GaN materials can be 136 times more effective than silicon (Table 1). These properties allow WBG devices to operate in extreme conditions, making them particularly valuable for high-power-density applications.

**Table 1:** Comparison of Si & WBG bulk materials physical properties [6, 11–15]

Material	${E}_{g}$(eV)	${E}_{crit}$(MV cm^−1^)	${\mu }_{n}$(cm^2^ V^−1^ s^−1^)	${\varepsilon }_{r}$	${n}_{i}$(cm^−3^)	$\lambda$@300 K (W cm^−1^ K^−1^)	BFOM*
Si	1.12	0.29	1350	11.9	1 × 10^10^	1.5	1
3C-SiC	2.35	1.5	900	9.7	1.5 × 10^–1^	3.2	29.4
4H-SiC	3.28	2.2	800	9.6	8 × 10^–9^	3.7	80.9
6H-SiC	2.96	3.2	370	9.6	5 × 10^–9^	4.9	116.1
GaN	3.4	2	1700	9	2 × 10^–10^	1.3	136
GaAs	1.42	0.4	8500	13.1	1.8 × 10^–6^	0.55	278.5
GaP	2.26	1	250	11.1	2.7 × 10^6^	1.1	27.75
Diamond	5.47	5.6	1800	5.7	1 × 10^–20^	20	5016.6

*BFOM: Baliga’s figure of merit for the power devices relative to Si:${\varepsilon }_{r}{ \mu }_{n} {E}_{crit}3$

**Fig. 2:**
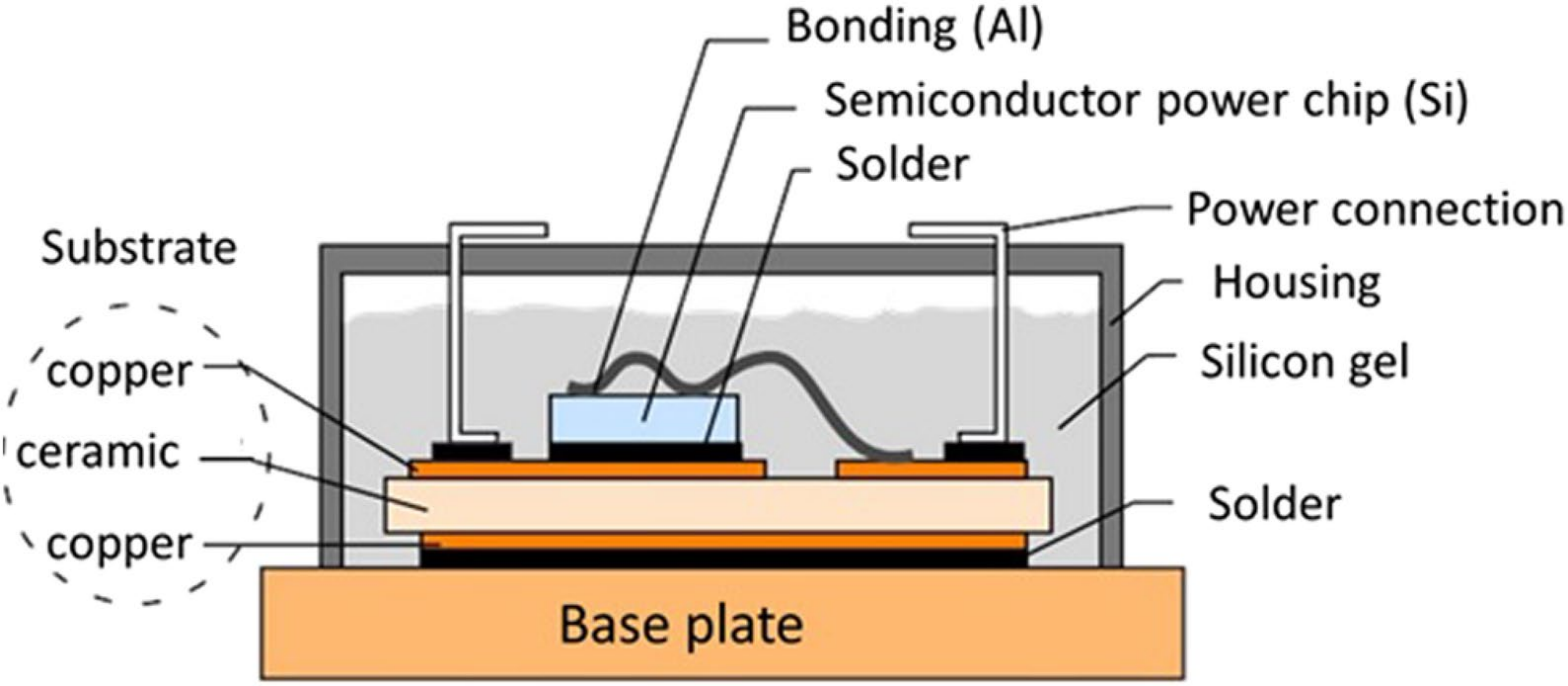
Schematic image of a WBG semiconductor package [Source [19]: T. Hamieh, Z. Khatir, and A. Ibrahim, "New solution of the partial differential equation of the grain groove profile problem in the case of evaporation/condensation", Scientific Reports, Vol. 9, 10143, 2019. © 2019 The Author(s). This article is licensed under a Creative Commons Attribution 4.0 International License.]

Despite these advantages, implementing WBG semiconductor devices introduces significant packaging challenges, particularly regarding die attach technologies [16–18]. Die attach materials serve as the critical thermal and electrical interface between the semiconductor die and the substrate in power electronic packages, as illustrated in Fig. 2. This interconnection layer must meet three critical requirements. First, it needs excellent thermal conductivity for heat dissipation. Second, it requires sufficient mechanical strength to withstand thermo-mechanical stress. Finally, it must maintain reliable electrical conductivity throughout the device's operational lifetime.

**Fig. 3:**
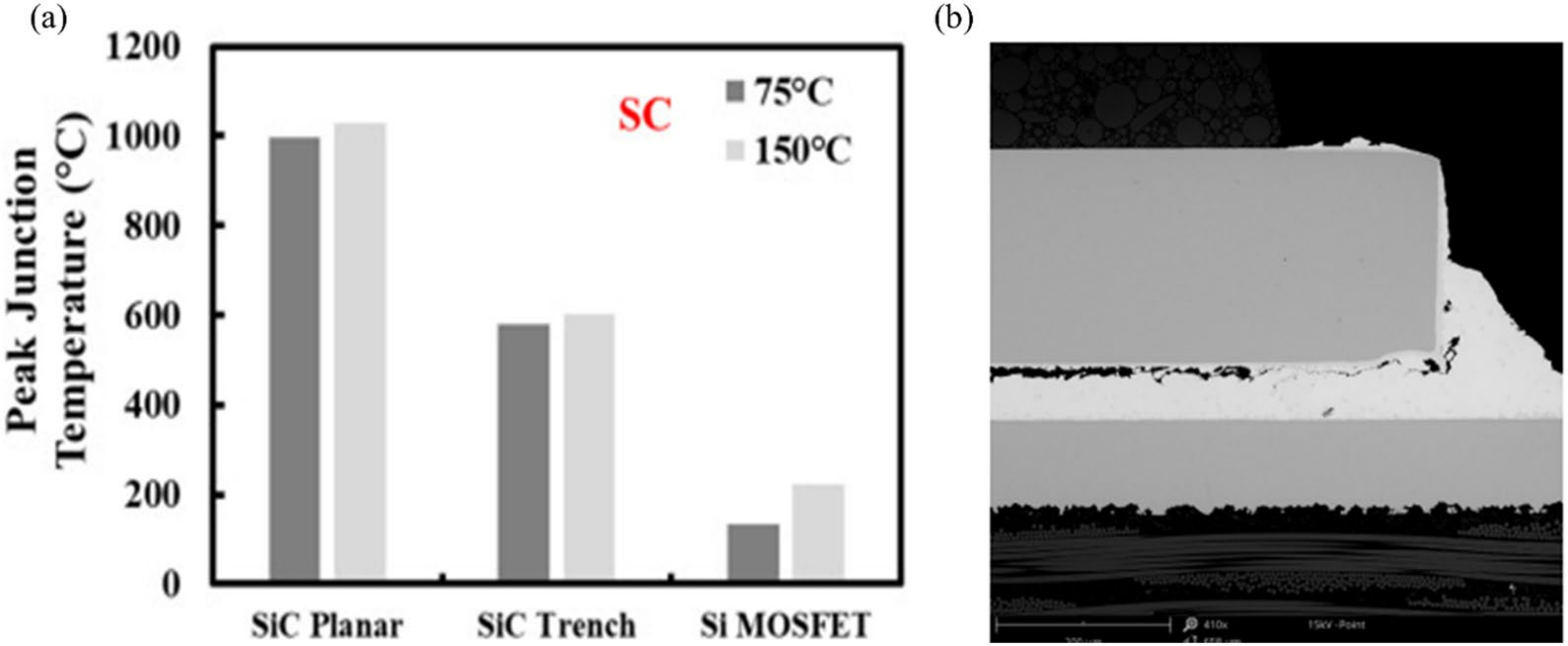
**a** Estimated peak junction temperatures during short-circuit testing [Source [20]: J. Ortiz Gonzalez et al., "Benchmarking the robustness of Si and SiC MOSFETs: Unclamped inductive switching and short-circuit performance," Microelectronics Reliability, November 2022. Licensed under CC BY]. **b** Cross-sectional SEM analysis of PKG after 5000 temperature cycles; massive delamination between the solder and gate is visible. [Source [44]: M. Calabretta et al., "Power Semiconductor Devices and Packages: Solder Mechanical Characterization and Lifetime Prediction," IEEE Access, Vol. 9. Licensed under CC BY 4.0.]

A critical distinction between WBG and conventional silicon-based semiconductors is their operating temperature range. Whereas traditional silicon devices typically operate at junction temperatures of 125–150 °C, WBG semiconductors function effectively at much higher temperatures, often exceeding 300–500 °C (Fig. 3a). This imposes severe thermal stress on the entire package, particularly the die-attach interface. Such stress can accelerate degradation mechanisms and compromise long-term reliability [20, 21]. Conventional silicon device packaging typically uses solders and sintering materials for die attachment. However, those materials have limitations for WBG devices: (1) inadequate thermal conductivity for efficient heat dissipation; (2) insufficient mechanical strength during thermomechanical cycling; and (3) poor stability at elevated temperatures. As shown in Fig. 3b, thermal aging of traditional solder joints leads to intermetallic compound (IMC) growth, which increases interfacial brittleness and ultimately causes mechanical failure [15].

To address those challenges, researchers have developed two primary methods that use nanomaterials for advanced die attach solutions: nanocomposite solders and nano-sintering technologies. The effectiveness of nanomaterials for performance enhancement has been well-established across various electronic applications, including flexible electronics [22], photovoltaic [23], composite electronics [24], bioelectronics [25], and energy storage devices [26, 27]. Based on these demonstrated successes, the integration of nanomaterials into WBG die attach technologies offers advantages through their superior reinforcement properties. Nanocomposite solders enhance conventional solder materials by incorporating nanoparticles. These particles improve mechanical properties, control IMC growth, and increase thermal conductivity [28–36]. These materials maintain processing compatibility with established manufacturing methods while addressing many of the limitations of lead-free solders. The nano-sintering technologies, particularly those based on silver nanoparticles, represent a more fundamental shift in die-attach methodology for WBG semiconductor packaging [37–40]. Unlike traditional soldering, these technologies do not rely on melting and solidification. Instead, they use the unique sintering behavior of nanoscale materials. This creates robust interconnects at relatively low processing temperatures (200–300 °C). The sintered joints offer excellent thermal performance. Their thermal conductivity typically exceeds 100–200 W m^−1^ K^−1^, compared with 30–70 W m^−1^ K^−1^ for conventional solders. In addition, the sintered silver joints exhibit remarkable high-temperature stability because of their high melting points approach that of bulk silver (961 °C). Whereas early silver sintering techniques required substantial external pressure (10–40 MPa) during processing, recent advances in pressure-less sintering processes have significantly improved manufacturing practicality by strategically using nanomaterials. Recent advances include optimized silver nanoparticle morphologies, bimodal particle size distributions, and surface-functionalized nanomaterials. These innovations enable effective sintering without external pressure [41–43].

**Fig. 4:**
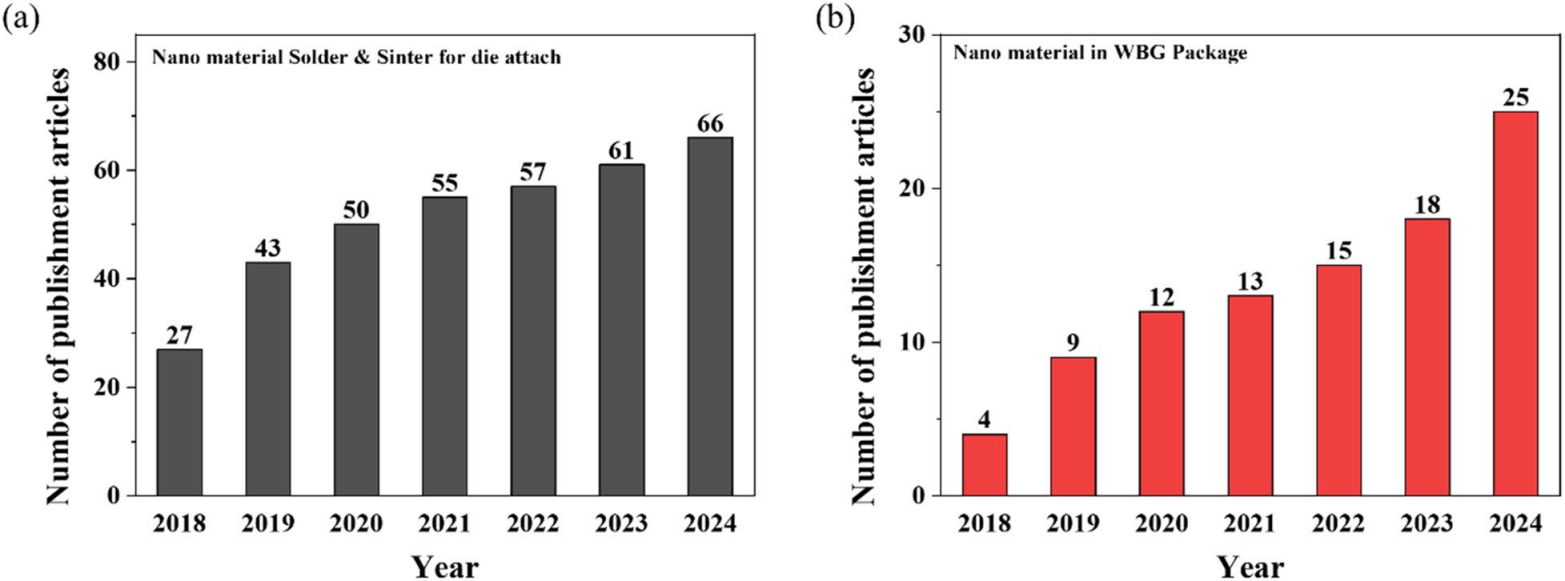
Publication trends from 2018 to 2024: **a** Nanomaterial-based die attach technologies [“Nanomaterial, die attach, solder, and sinter” on Google scholar] and **b** Nanomaterials specifically for WBG semiconductor packaging applications. [“Nanomaterial, die attach, solder, sinter, power electronics, and WBG semiconductor” on Google scholar**]**

Figure 4 shows the significant growth in research publications related to nanomaterial-based die attach technologies during the past several years. As shown in Fig. 4a, publications on nanomaterial solders and sintering for die attach have increased steadily from 27 articles in 2018 to 66 articles in 2024, reflecting growing interest in these advanced interconnection technologies. Similarly, Fig. 4b demonstrates the rising trend in publications specifically focused on nanomaterial applications in WBG packaging, with a six-fold increase from only 4 articles in 2018 to 25 articles in 2024. This substantial growth in research activity underscores the critical importance of nanomaterials when addressing the unique packaging challenges posed by WBG semiconductor devices.

In this review, we comprehensively analyze the current state-of-the-art nano-enabled die attach technologies for WBG power semiconductor packaging. While recent reviews have addressed related topics, this work provides a distinctly comprehensive perspective that differentiates it from existing literature. Paknejad and Mannan [45] focused on silver nanoparticle-based die attach materials for high-temperature applications, concentrating primarily on processing parameters and mechanical properties of nano-silver systems. Liang  et al. [46] reviewed nanoparticle-reinforced lead-free solder composites, emphasizing conventional nanocomposite solders without systematic analysis of advanced nano-sintering technologies. Cui et al. [47] reviewed shear strength and reliability of nanoparticle sintered joints for power electronics packaging, focusing predominantly on Ag and Cu sintering materials without comprehensive coverage of emerging nanomaterial architectures. While these reviews provide valuable insights into specific nanomaterial categories, none systematically integrates the various nanomaterials based on WBG semiconductor requirements.

This review covers advanced architectures such as core–shell nanostructures, transient liquid phase sintering (TLPS) systems, carbon-based nanocomposites, dendritic nanostructures, and novel nanowire reinforcement strategies that have emerged specifically for WBG semiconductor applications. We examine the fundamental mechanisms, processing parameters, and performance characteristics of both nanocomposite solders and nano-sintering approaches. Furthermore, we investigate the reliability of these materials in extreme operating conditions by evaluating their thermal cycling performance, shear strength stability, and microstructural evolution. This work provides the first comprehensive reliability analysis framework covering intrinsic, package, and module-field levels. We systematically connect diverse nanomaterial design principles with WBG device performance requirements under extreme operating conditions (200–300°C). By analyzing the current state of the art and identifying emerging trends across all nanomaterial categories, we provide valuable insights for optimizing die attach solutions for next-generation WBG power semiconductor applications.

## Nanocomposite solder for WBG power semiconductor packages

### Solder alloy for die-attach technology

Lead-based solders have been widely used in electronic assembly due to their excellent wetting properties, relatively low melting points, and good mechanical characteristics. However, environmental regulations such as the Restriction of Hazardous Substances directive have driven the industry toward lead-free solders [48, 49]. Currently, lead usage is restricted to specific applications with high reliability requirements, such as server-grade computing systems, electronic control units, military applications, and automotive components [49, 50].

**Fig. 5:**
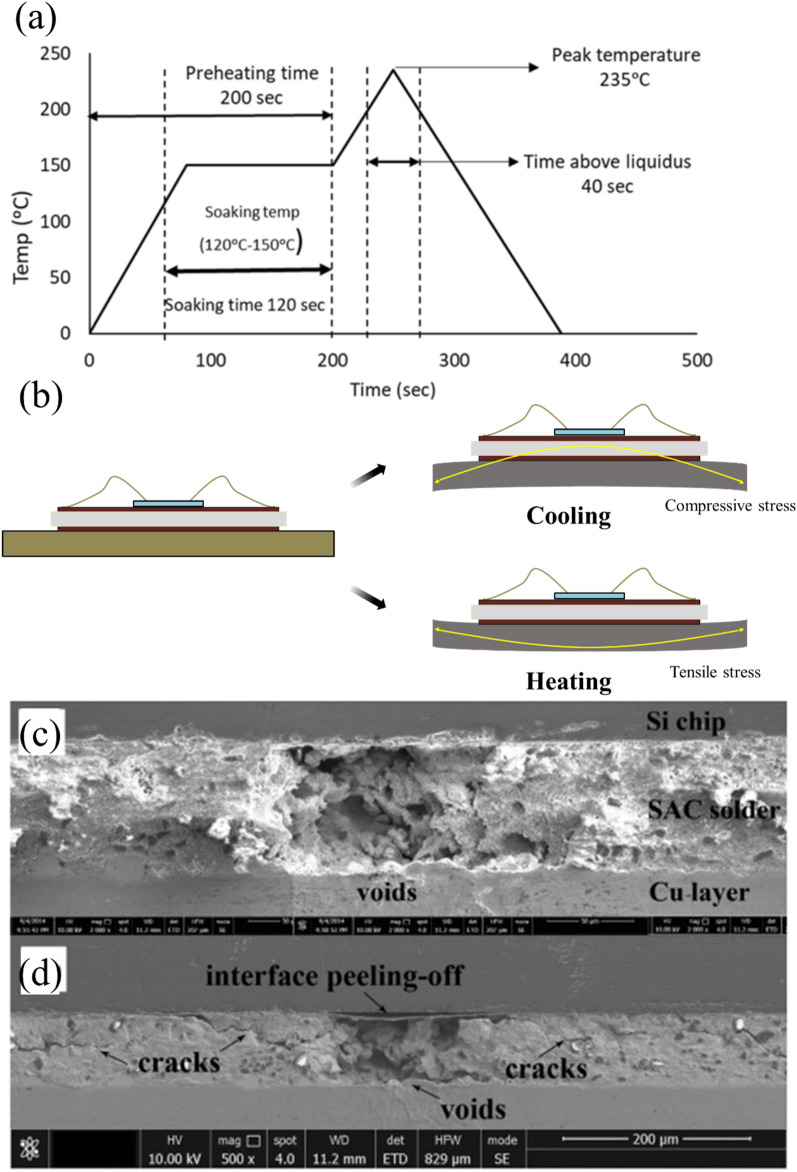
**a** Reflow profile for SAC305 solder joints [Source [78]: D. B. Hani, R. A. Athamneh, M. Abueed, S. Hamasha, "Neural-fuzzy machine learning approach for the fatigue-creep reliability modeling of SAC305 solder joints," Scientific Reports, Vol. 13, 8585, 2023. Licensed under CC BY 4.0.]. **b** power electronics package deformation due to CTE mismatch under cooling and heating condition. Morphology of the cross section of IGBT modules before and after power cycling: Δ*T*_*j*_ = 100 °C, *t*_*on*_/*t*_*off*_ = 1.00 s/1.00 s, *T*_*j_average*_ = 100 °C, **c** 0 cycles, **d** 10,000 cycles. [Source [57]: Reprinted from Microelectronics Reliability, Vol. 109, Huang Yongle, Luo Yifei, Xiao Fei, Liu Binli, Tang Xin, "Physics of failure of die-attach joints in IGBTs under accelerated aging: Evolution of micro-defects in lead-free solder alloys", pp. 113637, Copyright (2020), with permission from Elsevier.]

In response to environmental regulations, lead-free solders based on tin (Sn), particularly Sn–Ag–Cu (SAC) alloy systems, have emerged as the primary replacement [48, 51]. The predominant compositions of SAC alloy systems include SAC305 (Sn–3.0Ag–0.5Cu) in Japan, SAC387 (Sn–3.8Ag–0.7Cu) in the European Union, and SAC396 (Sn–3.9Ag–0.6Cu) in the United States [52]. Lead-free solders satisfy environmental requirements. However, their higher melting point compared to Sn–Pb solder creates challenges for WBG semiconductor packaging [53]. For Sn–Pb solders with a melting point of 183 °C, the typical reflow temperature is approximately 213 °C (melting point plus 30 °C). In contrast, SAC alloys have melting points around 217 °C. They require reflow temperatures of approximately 240 °C (Fig. 5a). This high temperature significantly increases the risk of substrate warpage. The warpage results from CTE mismatch between materials, causing reliability concerns (Fig. 5b) [54–56]. This issue is particularly critical for WBG power semiconductor packages that must operate reliably in high-temperature environments.

The SEM images in Fig. 5c–d clearly illustrate the severe degradation mechanisms that occur in SAC solder joints in thermal aging conditions. As shown in Fig. 5c, the as-bonded state of SAC solder exhibits a relatively intact interface with the silicon chip. However, significant microstructural deterioration can be seen, such as void formation, crack propagation, and interface delamination after thermal aging at 250 °C (Fig. 5d). A cross-sectional analysis reveals that thermal aging causes extensive damage to the solder layer, with voids serving as stress concentration points that facilitate crack initiation and propagation. This microstructural degradation leads to the significant reduction in shear strength reported by Yongle, Huang, et al. [57]. In addition, the observed interface peeling and crack formation create significant thermal barriers that impede efficient heat transfer from the die to the substrate. The vulnerability of conventional solder materials in high-temperature conditions clearly illustrates their fundamental limitations for WBG applications.

To address those challenges, nanocomposite solders have been investigated extensively. Nanoparticle additions improve mechanical and thermal performance through several key mechanisms. First, nanoparticles refine the microstructure through grain size control [58–60]. During solidification, they serve as heterogeneous nucleation sites, promoting the formation of numerous small grains. After solidification, they pin grain boundaries and prevent grain coarsening during thermal exposure. This fine-grained structure enhances strength according to the Hall–Petch relationship. Second, well-dispersed nanoparticles strengthen the matrix through dispersion hardening [61, 62]. They act as obstacles to dislocation movement, forcing dislocations to either bypass or accumulate at particle interfaces. This effect is particularly pronounced when nanoparticles are incoherent with the matrix. The lattice mismatch creates local strain fields that further impede dislocation motion, significantly increasing the resistance of material to plastic deformation.

Beyond mechanical reinforcement, nanoparticles play a crucial role in interfacial stability by IMC growth [33, 35, 63–65]. Nanoparticles act as physical barriers to the diffusion of substrate atoms (typically Cu) into the solder matrix, significantly reducing the growth rate of brittle interfacial IMCs during thermal aging. Furthermore, these nanoscale additives enhance creep resistance by pinning grain boundaries and sub-grain structures at elevated temperatures, which is particularly valuable during WBG device operation when persistent high-temperature exposure occurs [66–68]. The effectiveness of these mechanisms depends strongly on achieving uniform nanoparticle distribution throughout the solder matrix and maintaining stable interfaces between nanoparticles and the surrounding solder during both processing and operation.

Table 2 summarizes various solder alloys with nanoparticle additions. Based on the data presented in Table 2, nanocomposite solders can be categorized into two main groups, metal oxide nanoparticle-enhanced solders and metallic nanoparticle-enhanced solders. The metal oxide group incorporates various ceramic nanoparticles such as TiO_2_, Al_2_O_3_, ZrO_2_, SrTiO_3_, and ZnO into traditional Sn-based solder matrices. These composites generally maintain liquidus temperatures between 217 and 238 °C while significantly improving mechanical properties. In contrast, metal nanoparticle-reinforced solders containing Ag, Cu, or Ni nanoparticles exhibit remarkably low liquidus temperature (201–202 °C) and maintain excellent mechanical strength. In particular, Cu nanoparticle additions to SAC0705-BiNi demonstrated impressive diffusion control capabilities, reducing the IMC growth rate by nearly 60% during high-temperature aging at 180 °C. In addition, Ni-reinforced SAC solder showed excellent interfacial stability, with Cu_3_Sn suppression even after aging at 150 °C for up to 1008 h. These performance improvements highlight the effectiveness of adding nanoparticles to address the thermomechanical reliability challenges of WBG semiconductor packaging. Although both categories show promise, metal oxide nanoparticles have attracted more research attention due to their superior thermal stability, chemical inertness at elevated temperatures, and more consistent performance across the various operating conditions typical of WBG applications.

**Table 2:** Summary of solder with nanoparticles

Solder with nanoparticle system	Liquidus temperature (°C)	Mechanical properties	Reliability tests	References
*Metal oxide nanoparticle enhanced solders*				
Sn–0.7Cu–0.25TiO_2_	226.21	TS: 38.3 ± 2.72 MPa, HV: 11.56 ± 0.28	–	[69]
Sn–0.7Cu–0.5TiO_2_	226.24	TS: 42.08 ± 3.12 MPa, HV: 12.28 ± 0.42	–	[69]
Sn–0.7Cu–1TiO_2_	226.17	TS: 46.3 ± 1.68 MPa, HV: 12.8 ± 0.40	–	[69]
Sn–3.5Ag–0.5TiO_2_	226.6	TS: 47.1 ± 1.34 MPa, HV: 17.1 ± 0.56	–	[70]
Sn–3.5Ag–0.7Cu–0.5TiO_2_	224.1	TS: 51.9 ± 2.15 MPa, HV: 17.6 ± 0.63	–	[70]
Sn–3.5Ag–0.5Cu–0.25Al_2_O_3_	222.3	HV: 14.1	–	[71]
Sn–3.5Ag–0.5Cu–0.5Al_2_O_3_	222.7	HV: 17.0	–	[71]
Sn–3.5Ag–0.5Cu–1Al_2_O_3_	223.0	HV: 19.8	–	[71]
SAC–0.5ZrO_2_	217.08	SS: 40.7 MPa	TC: 39.2 MPa after 16 reflow cycles	[72]
SAC–3ZrO_2_	217.25	SS: 43.4 MPa	TC: 40.9 MPa after 16 reflow cycles	[72]
Sn–9Zn–ZrO_2_	~ 210 (est.)	SS: ~ 44 MPa (est.)	Increased shear stress after multiple reflows cycles	[73]
Sn–Ag–Cu–0.5SrTiO_3_	217.66	SS: 39.1 MPa	AT: 33.5 MPa after 150 °C/40 day, TC: 35.3 MPa after 16 reflow cycles	[74]
Sn–5Sb–0.5Cu–ZnO	238.27	TS: 86.35 MPa	–	[75]
*Metallic nanoparticle enhanced solders*			–	
Sn–9Zn–0.5Ag	201.21	TS: 41.3 MPa, BHN: 14	–	[76]
Sn–9Zn–1.0Ag	201.59	TS: 43.6 MPa, BHN: 16	–	[76]
Sn–9Zn–1.5Ag	201.96	TS: 37 MPa, BHN: 14.5	–	[76]
SAC0705–BiNi + 0.1nano-Cu	223.75	–	AT: diffusion coefficient 0.95 μm^2^/h at 180 °C/100 h	[77]
SAC0705–BiNi + 0.5nano-Cu	223.60	–	AT: diffusion coefficient 0.85 μm^2^/h at 180 °C/100 h	[77]
SAC0705–BiNi + 1.0nano-Cu	223.78	–	AT: IMC thickness 2.70 → 8.76 μm vs. 3.31 → 13.64 μm, diffusion coefficient 0.40 μm^2^/h versus 0.98 μm^2^/h	[77]
Sn–3.8Ag–0.7Cu–0.27Ni	216.8 ± 0.2	–	AT: uniform IMC formation, Cu_3_Sn suppression, improved solder joint reliability at 150 °C/504 h or 1008 h	[75]

TS: Tensile strength, SS: Shear strength, HV: Vickers hardness, BHN: Brinell hardness number, AT: Aging test, TC: Thermal cycling test, SAC:Sn–Ag–Cu alloy, est.:Estimate

### Nanoparticle-reinforced solder composites

Nanoparticles are the most extensively studied reinforcement materials for lead-free solder composites. They offer excellent thermal stability, mechanical properties, and chemical inertness at elevated temperatures. These properties enable the solder to withstand extreme operating conditions in WBG applications. Among the various additives, metal oxide nanoparticles such as NiO, TiO_2_, Al_2_O_3_, ZrO_2_, and SiO_2_ have demonstrated particularly promising results for enhancing the thermo-mechanical reliability of SAC-based solder matrices.

**Fig. 6:**
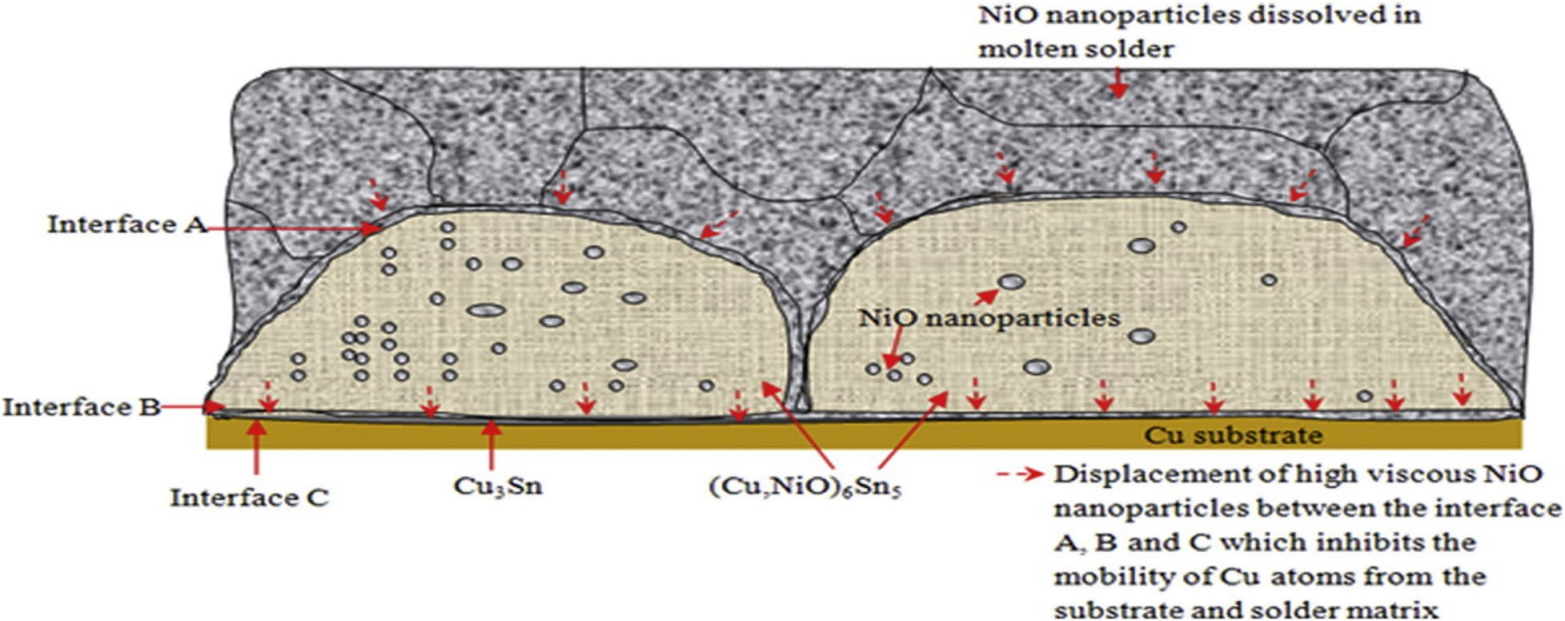
Schematic illustration of interfacial IMC formation and nanoparticle displacement in SAC 305–xNiO solder paste during reflow soldering [Source [58]: Reprinted from Materials & Design, Vol. 90, Srivalli Chellvarajoo, M.Z. Abdullah, "Microstructure and mechanical properties of Pb-free Sn-3.0Ag-0.5Cu solder pastes added with NiO nanoparticles after reflow soldering process", Pages 499–507, Copyright (2016), with permission from Elsevier.]

Chellvarajoo et al. [58] incorporated NiO nanoparticles into Sn–3.0Ag–0.5Cu (SAC 305) solder paste at 0.5, 1.5, and 2.5 wt.%. They found that the NiO nanoparticles dissolved into the molten solder during the reflow process, significantly influencing the interfacial microstructure. They reported that the previously thick and discontinuous Cu_6_Sn_5_ IMC layers observed in the plain SAC solder became thinner and more continuous with the addition of NiO. They attributed that transformation to the high viscosity and reactivity of NiO nanoparticles. As shown in Fig. 6, the NiO nanoparticles inhibited the diffusion of Cu atoms from the substrate, suppressing IMC growth. Moreover, a nano-indentation analysis showed a substantial enhancement in hardness of up to 91.8% with 2.5 wt.% NiO. Therefore, nanoparticles can effectively refine β-Sn grain structures and improve the microstructural characteristics and mechanical properties of solder joints.

**Fig. 7:**
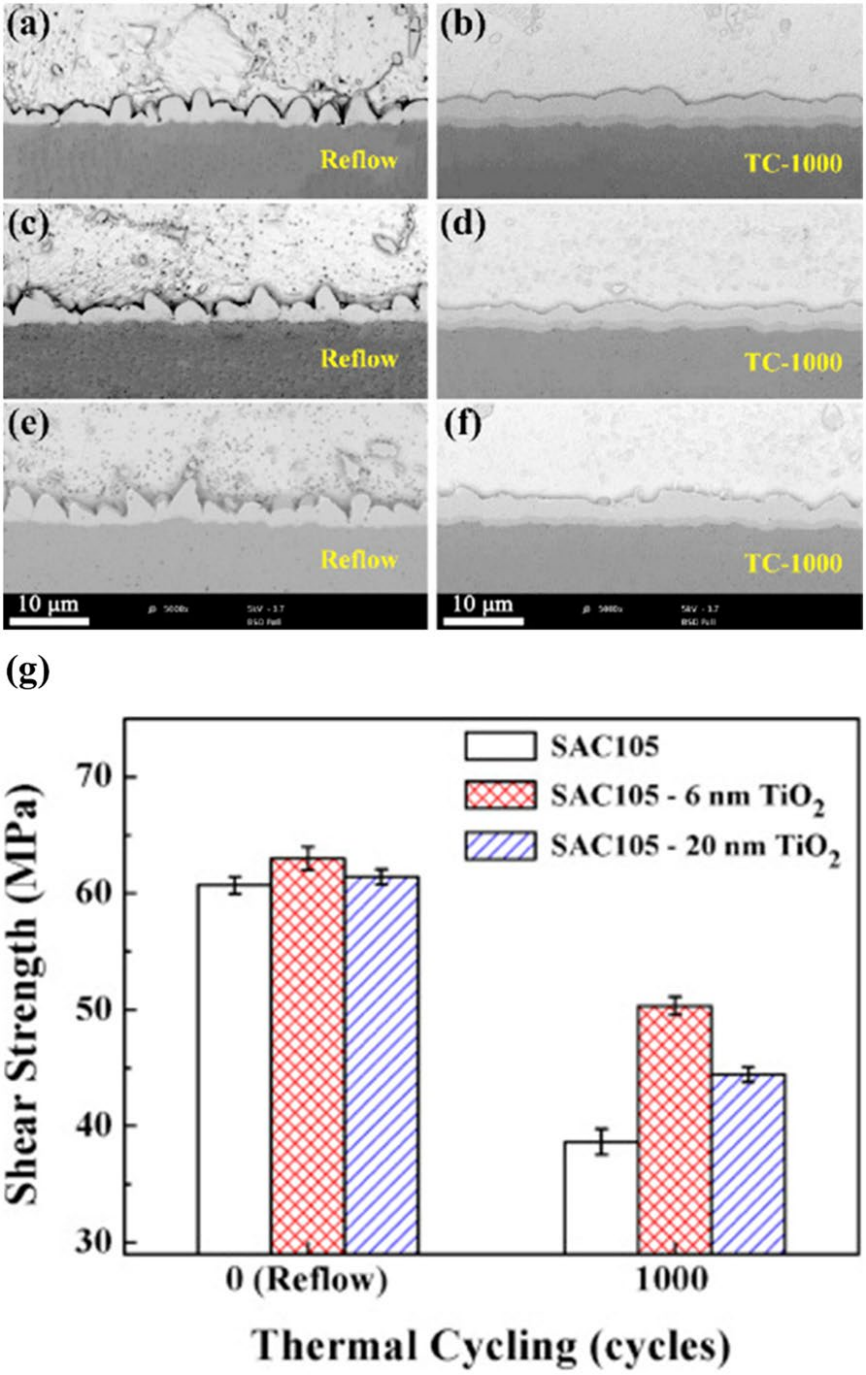
Cross-sectional images of solder/Cu interfaces after reflowing and TC for 1000 cycles (0.5 wt.% TiO_2_ nanoparticles): **a, b** SAC105, **c, d** SAC105–6nm TiO_2_, **e, f** SAC105–20nm TiO_2_, **g** Average shear strengths of solder/Cu joints after reflowing and TC for 1000 cycles. [Source [79]: Reprinted from Journal of Alloys and Compounds, Vol. 696, Yanni Wen, Xiuchen Zhao, Zhuo Chen, Yue Gu, Yong Wang, Zhiwei Chen, Xinyuan Wang, "Reliability enhancement of Sn-1.0Ag-0.5Cu nano-composite solders by adding multiple sizes of TiO_2_ nanoparticles", pp. 799–807, Copyright (2017), with permission from Elsevier.]

Tang et al. [63] reported that incorporating 0.1 wt.% TiO_2_ nanoparticles into Sn–3.0Ag–0.5Cu solder significantly affected IMC characteristics. The IMC layer thickness decreased from 13.15 to 10.33 μm. The average IMC grain size also reduced from 11.07 to 9.91 μm. They suggested that the combined effects of heterogeneous nucleation and grain-refinement mechanisms, produced a significantly more uniform and refined Cu_6_Sn_5_ IMC morphology. Wen et al. [79] further demonstrated the effectiveness of TiO_2_ nanoparticles in enhancing the thermal cycling reliability of lead-free solders. As shown in Fig. 7a–f, the addition of TiO_2_ significantly refined the β-Sn grain structure and suppressed interfacial IMC growth. Due to the inhibited IMC growth, the solder joint maintained higher shear strength after repeated thermal cycling. However, the SAC105 solder without nanoparticles showed significant degradation after 1000 thermal cycles. Therefore, the SAC105 with 6 nm TiO_2_ nanoparticles demonstrated better resistance to shear strength reduction than the control. Moreover, electrical resistance measurements during thermal cycling revealed sharp increases in resistance and crack formation in plain SAC105 joints at 125 °C. In contrast, the TiO_2_-reinforced joints remained electrically stable and free of large interfacial cracks in the same conditions. These results show the critical role of nanoparticles in mitigating thermal fatigue damage and improving both the mechanical and electrical reliability of solder joints during cyclic thermal stress.

Al_2_O_3_ nanoparticles have also demonstrated exceptional reinforcement capabilities in various lead-free solder systems. Gain et al. [80] reported that incorporating Al_2_O_3_ nanoparticles into SAC305 solder resulted in increase its elastic modulus and shear strength. The elastic modulus increased from approximately 51 GPa in the plain solder to 59 GPa with 1 wt.% Al_2_O_3._ The shear modulus also improved from 19 to 22 GPa. This enhancement remained stable across operating temperatures (25–170 °C), demonstrating that Al_2_O_3_-reinforced solders maintained higher elastic moduli than the unreinforced counterpart. The shear strength improvements were particularly notable. As the reaction time (the duration for which the solder is held at an elevated temperature during the soldering process) increased, the Al_2_O_3_-reinforced solders consistently outperformed the plain SAC solder. The 1 wt.% Al_2_O_3_ composite showed the highest shear strength values across all conditions. At 30 min reaction time, this composite maintained 40 MPa strength while plain solder degraded to 33 MPa. This represents a 20% improvement over the unreinforced material.

A SEM analysis further revealed that the Al_2_O_3_ nanoparticles distributed along grain boundaries, effectively refining the microstructure of the solder and slowing IMC growth. This microstructural refinement was attributed to a second phase dispersion strengthening mechanism provided by the Al_2_O_3_ nanoparticles. The surface-active ceramic nanoparticles accumulated at grain boundaries and in IMC layers. They played a pivotal role during solidification. This accumulation significantly affected the resulting microstructure. Additionally, Tsao et al. [62] investigated the reliability of Al_2_O_3_-reinforced Sn–3.5Ag–0.5Cu ball grid array joints after multiple reflows. They found that those containing 0.5 wt.% Al_2_O_3_ retained about 94% of their initial shear strength even after eight reflow cycles. In contrast, the unreinforced solder joints retained only about 91% of their shear strength. That enhanced reliability was attributed to the discrete particle effect of the Al_2_O_3_ nanoparticles on interfacial IMC growth, resulting in suppressed Cu_6_Sn_5_ formation at the interface and effective pinning of grain boundary movement.

**Fig. 8:**
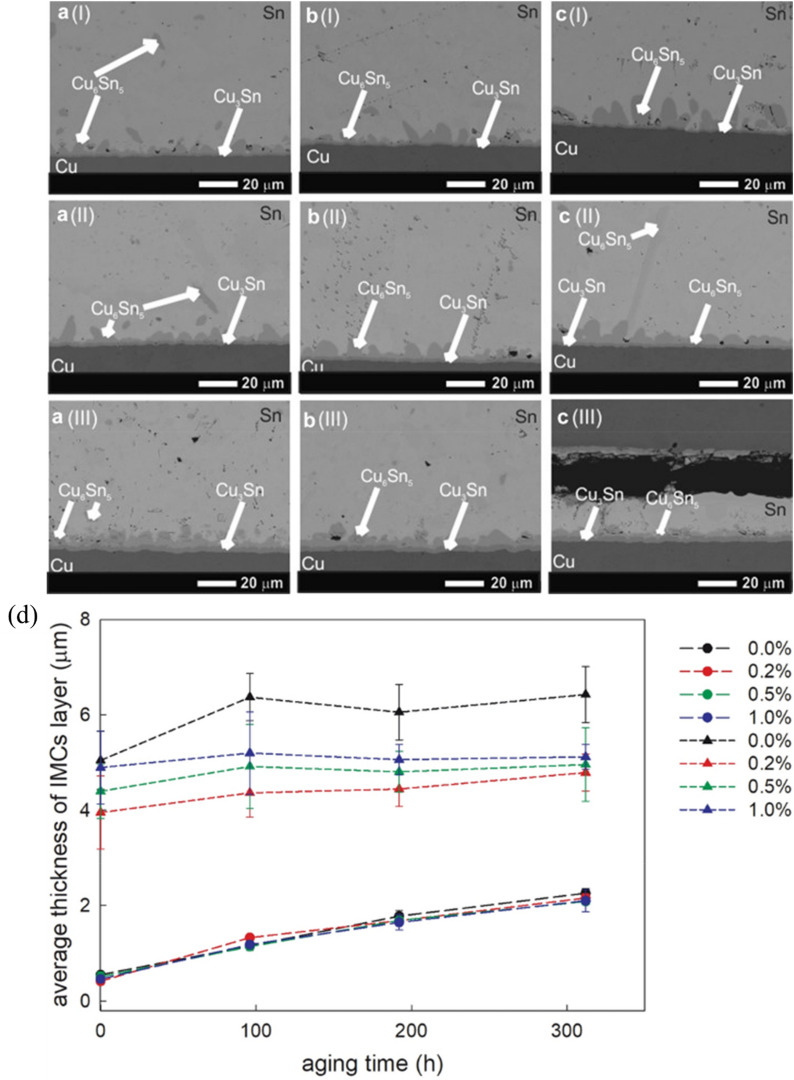
SEM micrographs showing the microstructural evolution of Cu/nanocomposite flux/Sn–3.5Ag/nanocomposite flux/Cu hybrid solder joints containing ZrO_2_ nanoparticles at: 0.2 wt.% (**a **(I–III)), 0.5 wt.% (**b **(I–III)), and 1.0 wt.% (**c **(I–III)) during thermal aging at 423 K for different durations: 96 h (I), 192 h (II), and 312 h (III) and **d** Quantitative analysis showing the average thickness of interfacial Cu_6_Sn_5_ (triangles) and Cu_3_Sn (circles) IMCs in samples containing different concentrations of ZrO_2_ nanoparticles (0.0%, 0.2%, 0.5%, and 1.0%) as a function of aging time at 453 K [Source [81]: Wodak, I., Yakymovych, A., Svec Sr., P. et al., "Hybrid solder joints: the effect of nanosized ZrO_2_ particles on morphology of as-reflowed and thermally aged Sn–3.5Ag solder joints", Applied Nanoscience, Vol. 13, 7379–7385, 2023. Licensed under CC BY 4.0.]

Thermal aging resistance is a critical factor determining the long-term reliability of solder joints in WBG semiconductor packaging. ZrO_2_ nanoparticles have demonstrated considerable potential in slowing IMC growth during prolonged thermal exposure. Wodak et al. [81] investigated Sn–3.5Ag hybrid solder joints incorporating nanosized ZrO_2_ particles via flux-assisted doping. They reported a notable suppression in the growth kinetics of Cu–Sn IMC layers during isothermal aging at 453 K (180 °C). The microstructural evolution analysis revealed that the ZrO_2_ nanoparticles fundamentally altered both the morphology and growth rate of interfacial IMCs. In conventional SAC solder joints, Cu_6_Sn_5_ IMCs typically exhibit a discontinuous scallop-type structure. It grows rapidly during thermal aging and leads to brittle failure. However, with the addition of ZrO_2_ nanoparticles, the interfacial Cu_6_Sn_5_ layer transformed from this scallop-like morphology to a more continuous, planar prism-like structure (Fig. 8a).

Figure 8a–c shows the thermally aged hybrid Sn–3.5Ag solder joints containing different concentrations of ZrO_2_ nanoparticles (0.2 wt.%, 0.5 wt.%, and 1.0 wt.%). The images labeled (I), (II), and (III) represent aging times of 96, 192, and 312 h at 453 K (180 °C), respectively. It clearly show how the initially scallop-shaped Cu_6_Sn_5_ and Cu_3_Sn layers evolve into more planar morphologies with increased nanoparticle content and aging time, particularly the samples with 1.0 wt.% ZrO_2_ (samples c(I)-c(III)). Figure 8d quantitatively demonstrates the effectiveness of nanoparticle additions in suppressing IMC growth during prolonged thermal exposures, by showing the average thickness of interfacial IMCs versus aging time with different concentrations of ZrO_2_ nanoparticles. Whereas the undoped samples (0 wt.%) show rapid IMC growth reaching approximately 7 μm after 300 h of aging, the samples with 1.0 wt.% ZrO_2_ maintain significantly thinner IMC layers of only about 5 μm in the same conditions.

Wodak et al. reported that the ZrO_2_ nanoparticles reduce the surface energy of the IMC layer and shift the IMC growth rate toward a more planar direction. This mechanism significantly reduces the growth velocity and overall thickness of the Cu_6_Sn_5_ interfacial layer. This morphological change significantly effects joint reliability. Because excessive IMC growth typically increases brittleness at the interface. Therefore, restricting IMC growth by the adsorption of ZrO_2_ particles on IMC grain boundaries allows the solder joint to maintain better mechanical properties during extended thermal exposure. This property is particularly critical for WBG semiconductor packages that must withstand higher operating temperatures and more severe thermal cycling conditions than conventional silicon-based devices.

Recent studies have explored more complex nanoparticle systems to enhance solder reliability. Fouzder et al. [74] investigated the effects of adding SrTiO_3_ nanoparticle to SAC305 solder. They found that adding 0.5 wt.% yielded the optimal combination of mechanical properties and thermal stability. The SrTiO_3_-reinforced solder exhibited a shear strength of 39.1 MPa with minimal degradation after thermal aging at 150 °C for 40 days, retaining approximately 85.7% of its initial strength.

**Fig. 9:**
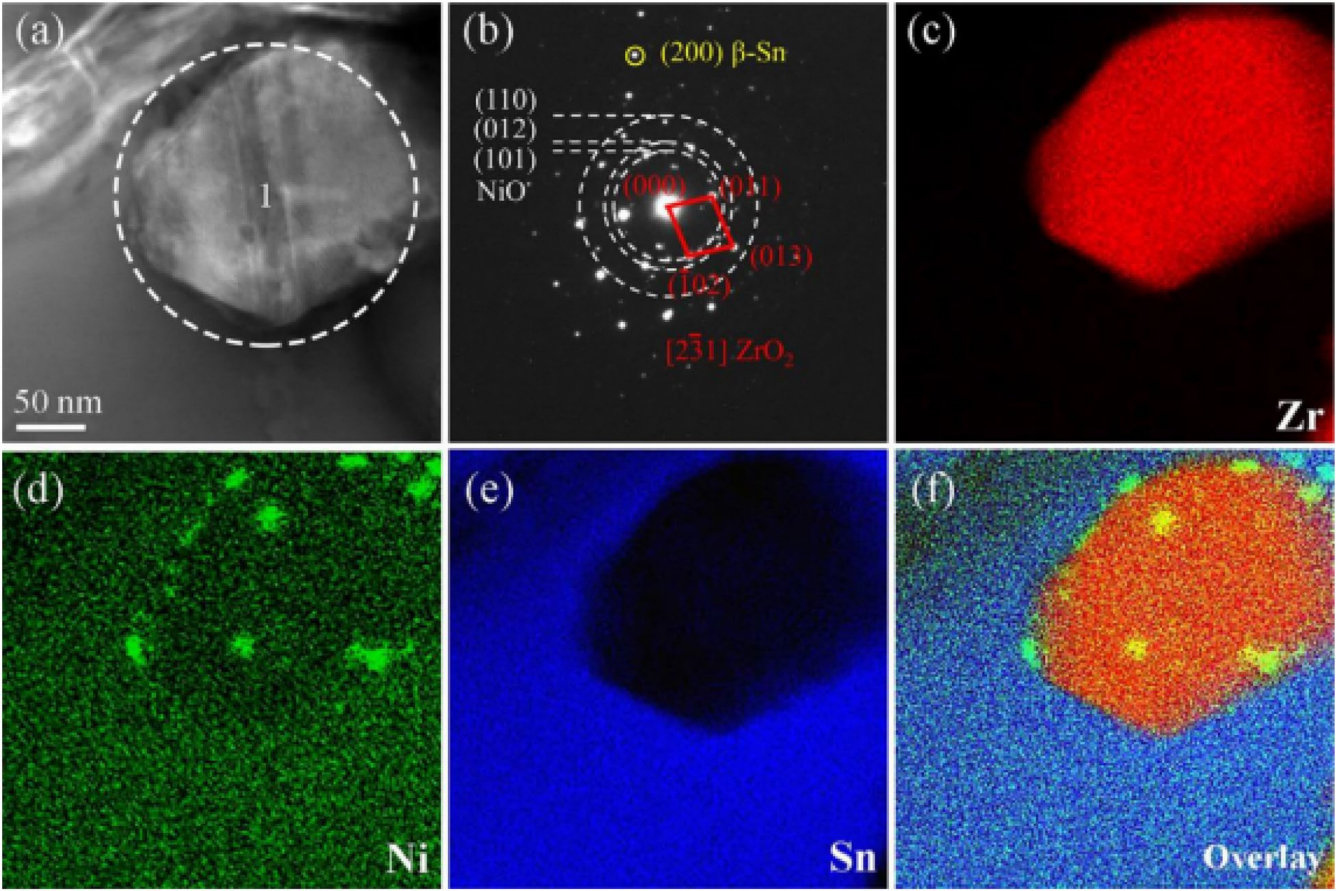
**a** TEM image of the interface between the reinforcements and solder matrix, **b** SAED patterns of area 1 in **a**. EDS mappings of **c** Zr, **d** Ni, and **e** Sn, and **f** their overlay. [Source [82]: Reprinted from Journal of Materials Science & Technology, Vol. 125, Huo, F., Jin, Z., Le Han, D. et al., "Novel interface regulation of Sn1.0Ag0.5Cu composite solders reinforced with modified ZrO2: Microstructure and mechanical properties", pp. 157–170, Copyright (2022), with permission from Elsevier.]

In addition, Huo et al. [82] investigated Sn1.0Ag0.5Cu composite solders reinforced with NiO-modified ZrO_2_ nanoparticles at various concentrations. As shown in Fig. 9(a–f), an appropriate addition of NiO/ZrO_2_ could refine the microstructure of composite solders because the particles evenly adhere to the ZrO_2_ surface. The nanoparticles acted as heterogeneous nucleation sites and created pinning effects at the grain boundaries, refining both the β-Sn matrix and eutectic structure while inhibiting grain boundary migration. This refined microstructure significantly improves both mechanical properties and thermal stability.

According to the literature, the optimal addition of 0.3 wt.% NiO/ZrO_2_ refined the microstructure, reduced the IMC size, and improved wettability (lowest contact angle of 8.75°). At that concentration, the ultimate tensile strength increased from 27.9 to 34.8 MPa (24.7% improvement) and elongation improved from 21.6 to 27.9% (29.2% increase). In addition, the fracture mode evolved from a ductile–brittle mixed fracture to a ductile fracture. This transformation confirmed the role of NiO/ZrO_2_ in promoting mechanical stability and interfacial bonding through effective load transfer across the engineered interface. Furthermore, the composite solder showed better durability during thermal aging tests. After 1008 h at 150 °C, it maintained superior structural and mechanical integrity compared to plain solder. This demonstrates excellent resistance to thermal degradation, particularly IMC coarsening. These results demonstrate that the strategically designed Sn/NiO/ZrO_2_ interface system offers a viable pathway for developing high-performance die attach materials for next-generation WBG power semiconductor packaging.

Conventional lead-free solders exhibit limited performance in the high operating temperatures (200–300 °C) and thermomechanical stresses of WBG semiconductors. To address those challenges, researchers have developed nanocomposite solders that incorporate various nanomaterials into conventional solder matrices. These nanocomposite solders exhibit improved performance by multiple strengthening mechanisms, including grain refinement, dispersion strengthening, and IMC growth inhibition. Our comprehensive review of the literature demonstrates that both metal oxide nanoparticles (TiO_2_, Al_2_O_3_, ZrO_2_, SrTiO_3_) and metallic nanoparticles (Ag, Cu, Ni) significantly improve mechanical strength, thermal stability, and reliability when they are incorporated into traditional Sn-based solder systems. These enhancements directly address the critical reliability requirements of WBG semiconductor packaging, offering promising pathways toward next-generation high-temperature power electronic applications.

### Advanced nanocomposite solder architectures

Beyond simple nanoparticle additions, researchers have developed more sophisticated nanocomposite solder architectures to address the specific requirements of WBG power semiconductor packaging. These advanced structures include carbon-based nanocomposites, transient liquid phase sintering systems, and core–shell nanostructures.

#### Carbon-based nanocomposites

**Fig. 10:**
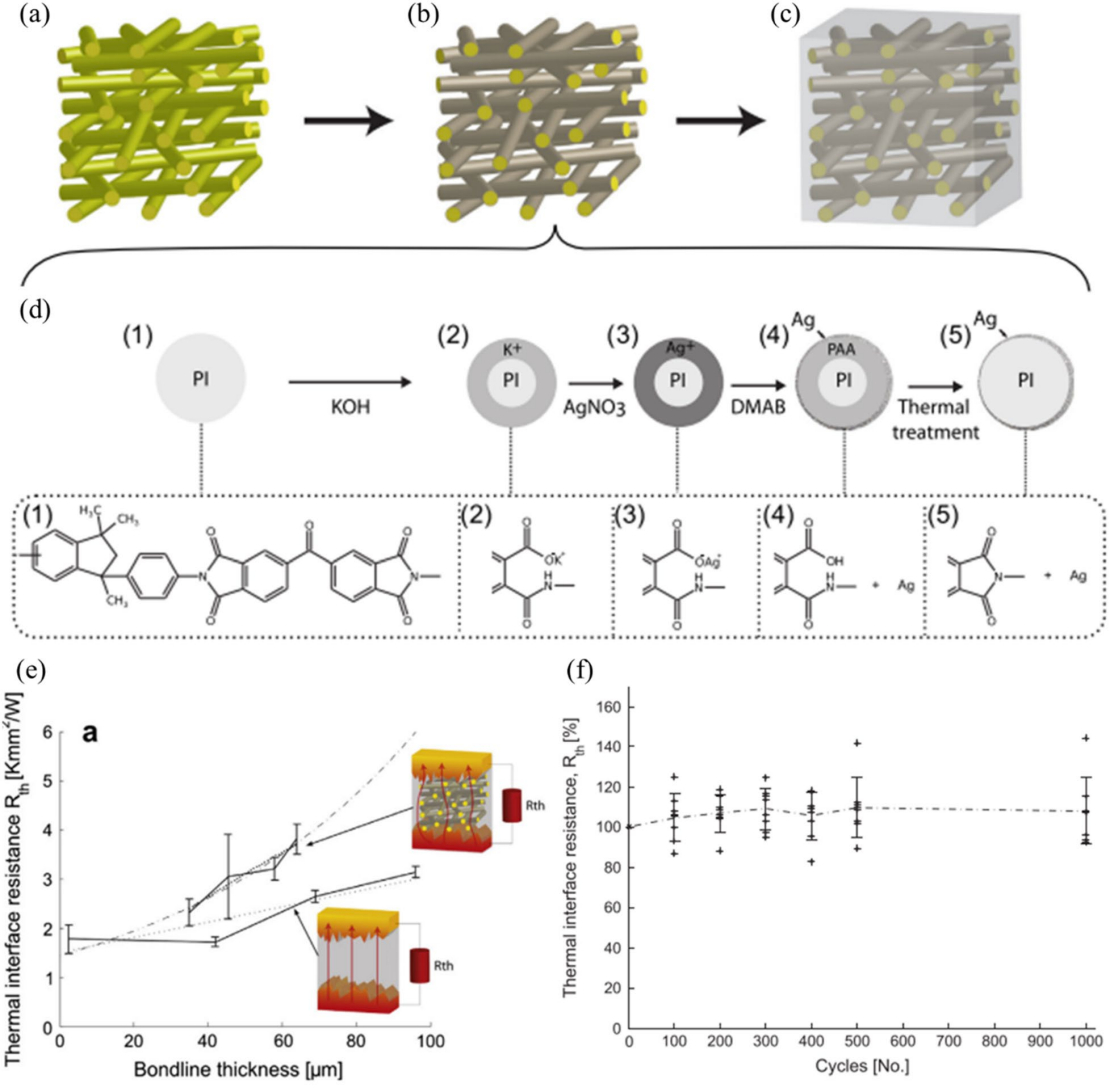
Schematic illustrating the fabrication process of the SMNPC: **a** The fiber carrier is formed through electrospinning, **b** Chemical reduction-based plating of Ag nanoparticles onto the fiber surface, and **c** Pressure -assisted infiltration of the metal matrix, **d** The major sub-steps in the plating process: (1) washing with ethanol, (2) imide cleavage induced from KOH, (3) ion exchange of K+ to Ag+ in AgNO3, (4) Ag+ reduction using DMAB, and (5) re-imidization through thermal treatment (5), **e** Thermal interface resistance as a function of the BLT of the solder matrix nano polymer composite and pure Sn–Ag–Cu alloy and ENIG–coated Cu and, **f** Change in thermal resistance during thermal cycling. [Source [36]: Reprinted from Composites Science and Technology, Vol. 94, Zandén, C., Luo, X., Ye, L. et al., "A new solder matrix nano polymer composite for thermal management applications", pp. 54–61, Copyright (2014), with permission from Elsevier.]

The incorporation of carbon-based nanomaterials, such as carbon nanotubes, graphene, and carbon fibers, offers dramatic improvements in thermal conductivity while maintaining or enhancing mechanical properties. Zandén et al. [36] introduced a novel solder matrix nano polymer composite (SMNPC) designed to address both the thermal and mechanical challenges of high-power electronics. The SMNPC is composed of a nano-silver, surface-functionalized, highly porous, electrospun non-woven, polyimide fiber network, embedded in a Sn95.5–Ag3.8–Cu0.7 alloy matrix. Figure 10a–d shows an overview of the SMNPC fabrication process in three sequential steps: the creation of a polyimide fiber network, its surface modification with silver nanoparticles, and embedding the functionalized structure into the SAC solder matrix.

Figure 10e shows the total thermal interface resistance at various bond line thicknesses (BLTs). It demonstrates that thinner bond lines exhibit lower thermal resistance, while thicker bonds show higher resistance due to the increased length of the heat conduction path. Importantly, the SMNPC demonstrates exceptional thermal stability during thermal cycling (Fig. 10f), maintaining its thermal interface resistance at approximately 80% of its initial value even after 1000 thermal cycles. This remarkable reliability can be attributed to the mechanical reinforcement provided by the polymer network. The network prevented crack propagation and inhibited the formation of voids during thermal cycling, serving as a mechanical framework that maintained structural integrity by distributing thermal stresses throughout the solder joint. That distribution reduced the strain concentration that typically leads to failure in conventional solder interfaces.

**Fig. 11:**
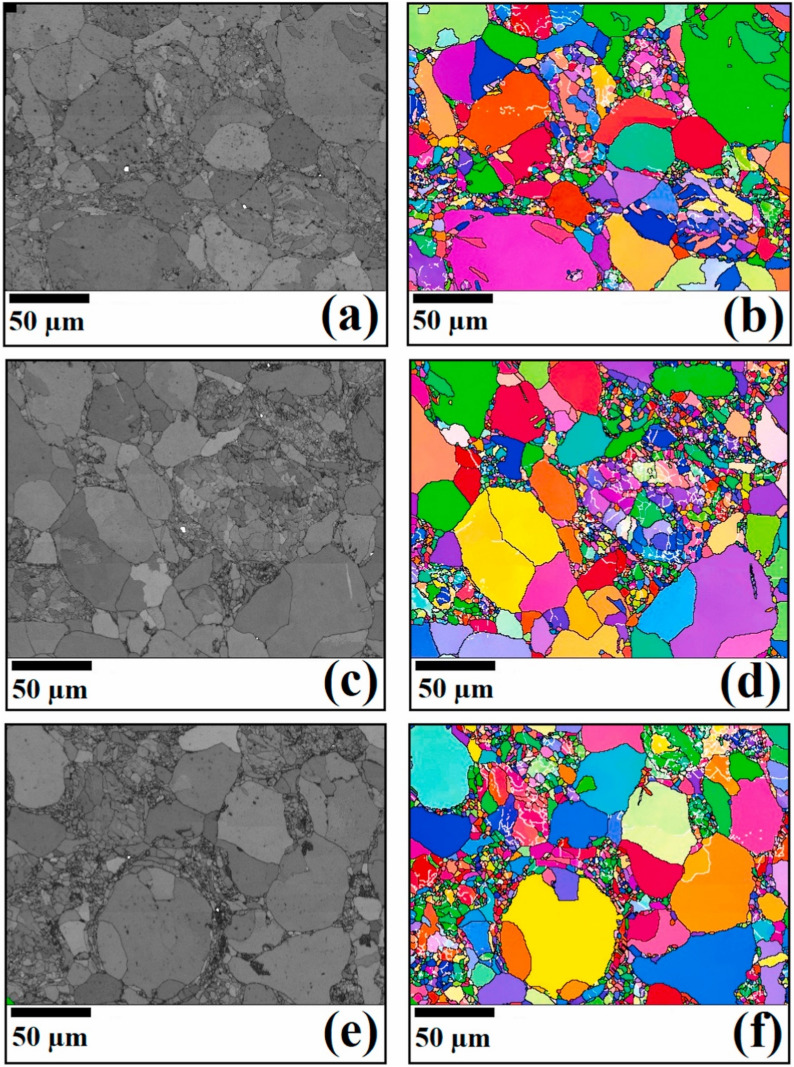
EBSD analysis results illustrating the **a, c, e** Grain structure and **b, d, f** Orientation maps of the prepared solder materials: **a**, **b** SAC solder alloy, **c**, **d** SAC/0.1 wt.% Ni-coated MW-CNT, and **e**, **f** SAC/0.2 wt.% Ni-coated MW-CNT solder nanocomposites. [Source [83]: Reprinted from Materials Science and Engineering, Vol. 797, Khodabakhshi, F., Zareghomsheh, M., Khatibi, G., "Nanoindentation creep properties of lead-free nanocomposite solders reinforced by modified carbon nanotubes", 140203, Copyright (2020), with permission from Elsevier.]

Creep occurs when die-attach materials face high temperatures and stress. The resulting deformation increases thermal resistance and promotes interfacial cracking. This ultimately reduces power-cycle reliability. Therefore, high creep resistance through the integration of nanomaterials is essential for long‑term thermal and mechanical stability. Khodabakhshi et al. [83] investigated how Ni-coated multi-walled carbon nanotubes (MW-CNTs) affect the properties of SAC alloys. Figure 11a–f shows the electron backscatter diffraction (EBSD) analysis of the SAC solder microstructure and corresponding orientation maps for different Ni-coated MW-CNT contents. These EBSD maps reveal that uniformly dispersing a small amount (≤ 0.1 wt.%) of MW-CNTs refines and stabilizes the grain structure. This improvement is attributed to the Ni-coated CNTs pinning dislocations and anchoring grain boundaries. This mechanism promotes Hall–Petch strengthening and suppresses both diffusion- and sliding-controlled creep pathways.

**Fig. 12:**
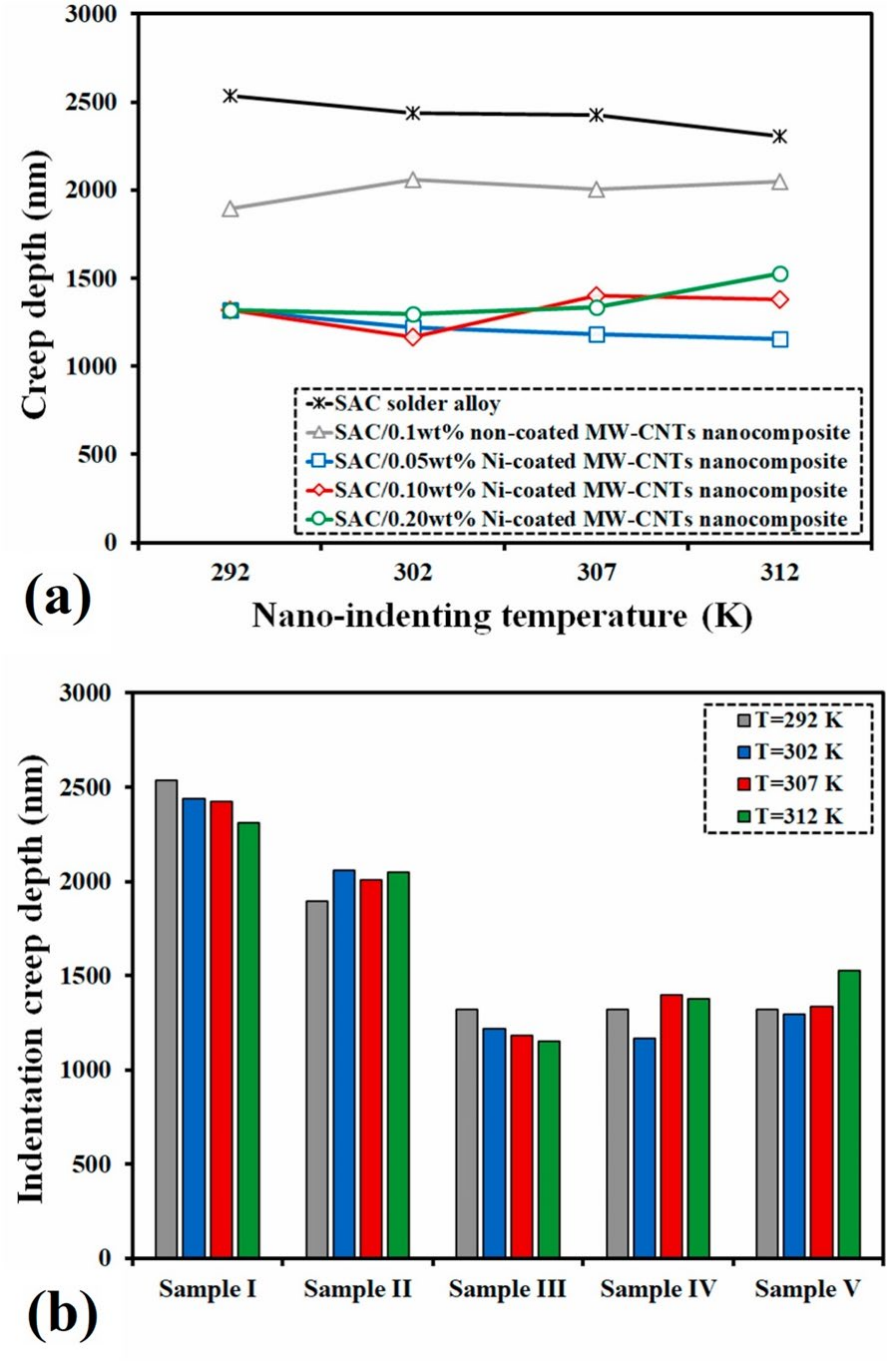
**a** Variations in creep depth for solder alloy and different solder nanocomposites versus the nano-indentation temperature after a holding time of 1200 s, **b** Histogram plots for the indentation creep depth of the solder alloy and nanocomposites at different testing temperatures for a holding time of 1200 s. [Source [83]: Reprinted from Materials Science and Engineering, Vol. 797, Khodabakhshi, F., Zareghomsheh, M., Khatibi, G., "Nanoindentation creep properties of lead-free nanocomposite solders reinforced by modified carbon nanotubes", 140203, Copyright (2020), with permission from Elsevier.]

Figure 12a presents the nanoindentation creep depth at 292–312 K (18.85–38.85 °C) and Fig. 12b shows a histogram comparing creep depth across different nanocomposites after 1200 s under constant load. These results confirm that an optimal MW-CNT content (around 0.1 wt.%) markedly improves creep resistance. However, excessive addition (> 0.1 to 0.2 wt.%) leads to agglomeration and local inhomogeneities. Agglomeration can reduce creep performance by inhibiting the pinning effect at grain boundaries. Notably, Ni‑coating the MW‑CNTs further enhanced interfacial bonding with the solder matrix, as evidenced by improved metallurgical compatibility and reduced creep deformation. These findings suggest that Ni‑coated CNT‑reinforced SAC alloys can effectively maintain their structural integrity under both thermal and mechanical stresses, making them promising candidates for next‑generation WBG power semiconductor packaging applications requiring reliable high‑temperature operation.

#### Transient liquid phase sintering (TLPS)

TLPS is an innovative approach to creating high-temperature-stable interconnects while using relatively low processing temperatures. The main characteristic of TLPS bonding is the use of a low-melting-point metal such as Sn or Bi. During processing, this metal temporarily melts and diffuses into a higher melting metal, creating intermetallic phases with significantly elevated melting points. The key advantage of TLPS is that it allows assembly at relatively low temperatures (typically 200–250 °C) while providing joints capable of withstanding operating temperatures exceeding 300–400 °C [84].

Therefore, TLPS-based interconnects can widely be used in power semiconductor packaging and electronics that operate in harsh environments. They offer stronger mechanical properties and better thermal reliability than traditional solder joints [85, 86]. TLPS materials can range from Sn- or Bi-based systems to precious-metal alloys (e.g., Au–Sn), and they can be applied via paste, preform, or plating methods according to design requirements. Depending on the process parameters, either solid-state diffusion or liquid-state reactions may dominate. Single and multi-step heat treatments can be used to tailor microstructures and optimize device performance [84, 87]. This flexibility allows manufacturers to fine-tune the device performance characteristics. In summary, TLPS bonding is a reliable method for creating thermally stable, high-performance electronic connections. This technology is particularly valuable in industries that required advanced packaging solutions for increasingly demanding applications.

**Fig. 13:**
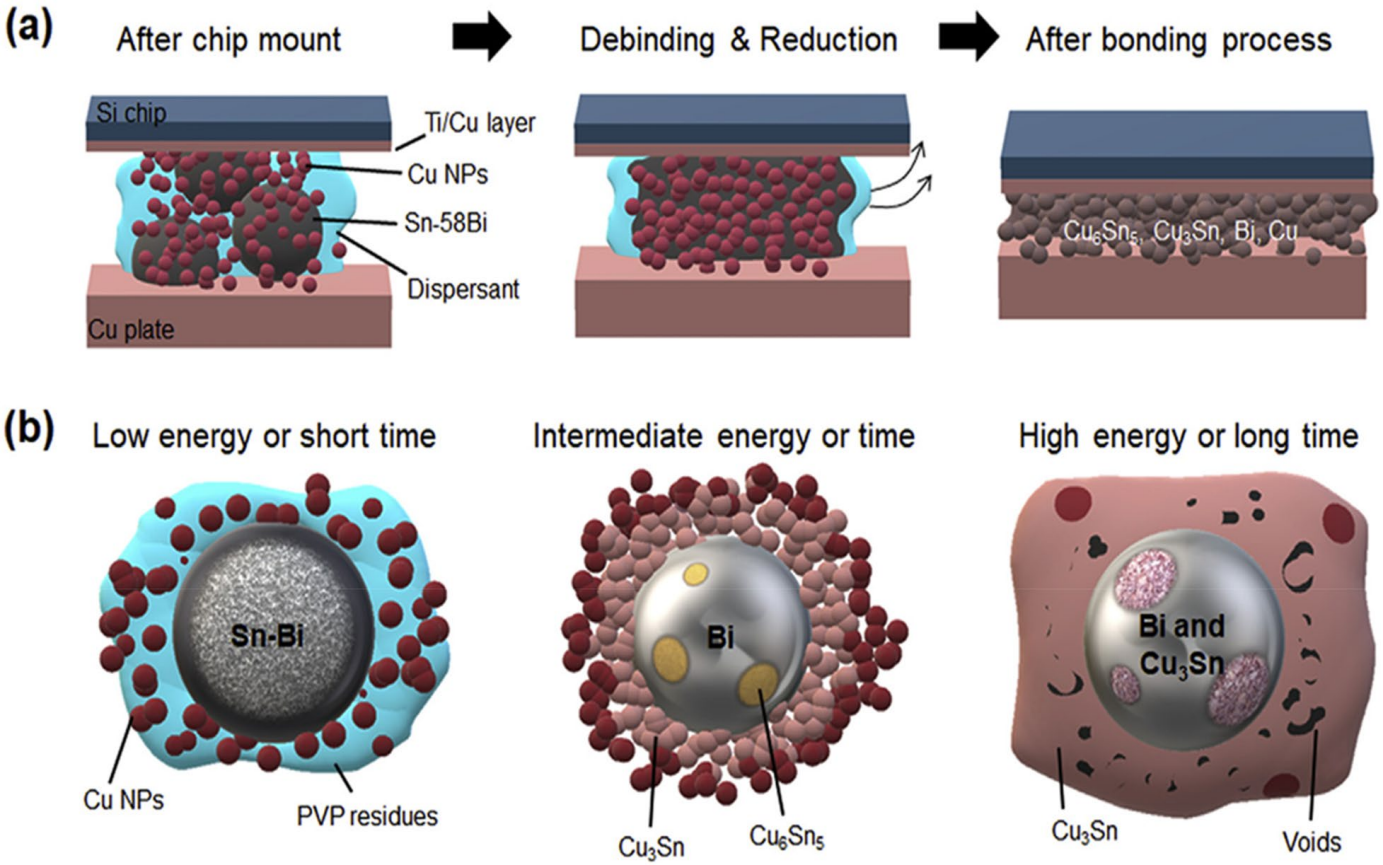
Schematic diagrams of **a** the TLPS bonding process and **b** TLPS reactions in various conditions of thermal energy and bonding time. [Source [88]: Reprinted from Journal of Alloys and Compounds, Vol. 781, Jung, K.-H., Min, K.D., Lee, C.-J. et al., "Pressureless die attach by transient liquid phase sintering of Cu nanoparticles and Sn-58Bi particles assisted by polyvinylpyrrolidone dispersant", pp. 657–663, Copyright (2019), with permission from Elsevier.]

Building on the TLPS fundamentals, Jung et al. [88] produced a detailed investigation of pressure-less die attach made using Cu nanoparticles (NPs), Sn-58Bi particles, and polyvinylpyrrolidone (PVP), as a dispersant. That study attempted to use nanomaterials for TLPS bonding at a relatively low temperature (190–250 °C). As shown in Fig. 13, they used the transient melting of Sn-58Bi to wet Cu NPs, followed by IMC formation and sintering. A significant advance in that research was the precise regulation of the PVP molecular weight, which effectively optimized the dispersion characteristics of the nanoparticles. That optimization substantially reduced void formation and organic residue content while enhancing the uniformity of microstructural development. Although the achieved shear strengths of approximately 7 MPa without bonding pressure was below commercial requirements for high-reliability power electronics, this pioneering work demonstrates the potential of nanomaterial-based TLPS for die attach applications. It established important fundamentals about the relationships among processing parameters, microstructural development, and the mechanical properties of nano-enabled TLPS systems, creating a foundation for subsequent advances in the field.

**Fig. 14:**
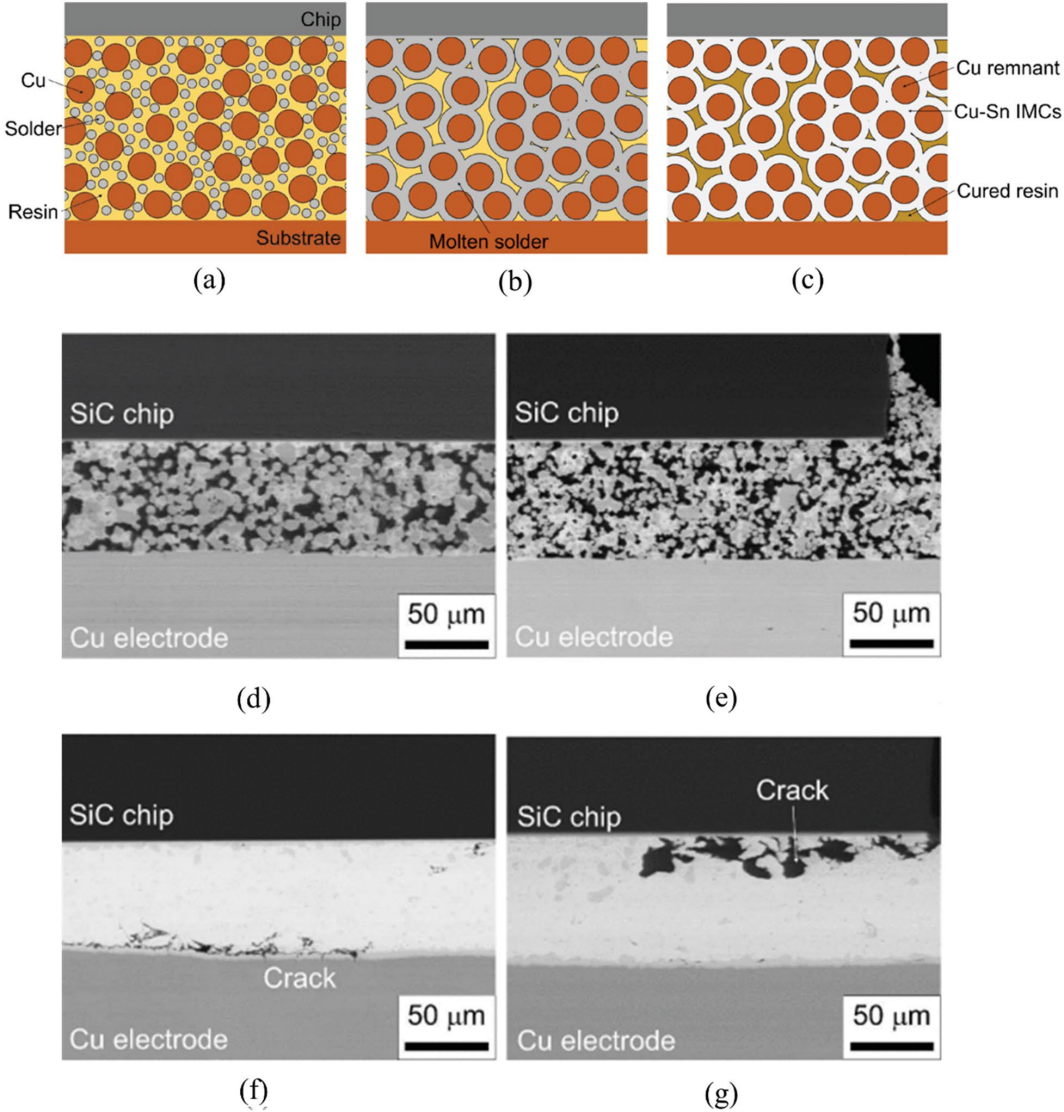
Schematics of the TLPS process using a Cu-solder-resin composite **a** Before, **b** During, and **(c)** After processing, and cross-sectional SEM image of **d, e **a TLPS joint after 1200 thermal cycles and **f–g** a SAC305 solder joint after 600 thermal cycles from − 55 to 175 °C [Source [89]: Reprinted with permission from IEEE Transactions on Components, Packaging and Manufacturing Technology. H. Tatsumi, A. Lis, H. Yamaguchi, Y. Kashiba, and A. Hirose, "Evaluation of Stiffness-Reduced Joints by Transient Liquid-Phase Sintering of Copper-Solder-Resin Composite for SiC Die-Attach Applications", IEEE Transactions on Components, Packaging and Manufacturing Technology, Vol. 9, No. 10, pp. 2111-2121, 2019. © 2019 IEEE]

Furthermore, Tatsumi et al. [89] demonstrated that incorporating a polyimide resin matrix into Cu–Sn TLPS joints significantly enhanced thermal cycling reliability through the formation of a unique skeleton-shaped microstructure. As shown in Fig. 14a–c, the microstructure consisted of Cu particles interconnected by Cu–Sn IMCs, with polyimide resin partially filling the interstitial spaces. During the TLPS process, the solder melted and reacted with Cu particles to form Cu–Sn IMCs while the resin cured in the vacant spaces, creating a composite structure that combines metallurgical bonding strength with mechanical compliance. The cross-sectional SEM images in Fig. 14d–g reveal the superior reliability of these composite joints over conventional SAC305 solder joints after thermal cycling. After 1200 thermal cycles between − 55 and 175 °C, the TLPS joints (Fig. 14d–e) maintained their structural integrity with no observable crack propagation, whereas the SAC305 solder joints (Fig. 14f–g) exhibited significant cracking after only 600 cycles. This enhanced performance is attributed to the reduced stiffness of the composite structure. The lower stiffness allows better accommodation of thermomechanical stresses from CTE mismatch between the SiC chip and substrate.

#### Core–shell nanostructures

Core–shell nanostructures have attracted growing interest in solder technology due to their ability to combine a high-melting-point core material (e.g., Cu or Ag) with a low-melting alloy shell (e.g., Sn) [90–92]. By encapsulating core particles in a reactive metal layer, these nanostructures allow for rapid wetting and diffusion-driven bonding at relatively low temperatures, while still achieving a mechanically robust joint after solidification. The shell provides initial liquid-phase flow and surface activation, mitigating oxidation and promoting uniform interfacial contact [93, 94]. Meanwhile, the remaining core preserves high-temperature stability, leading to excellent mechanical strength and thermal reliability in harsh environments [90]. As a result, core–shell solder nanoparticles balance the demands for low reflow temperatures and high reliability in advanced power electronic packaging.

**Fig. 15:**
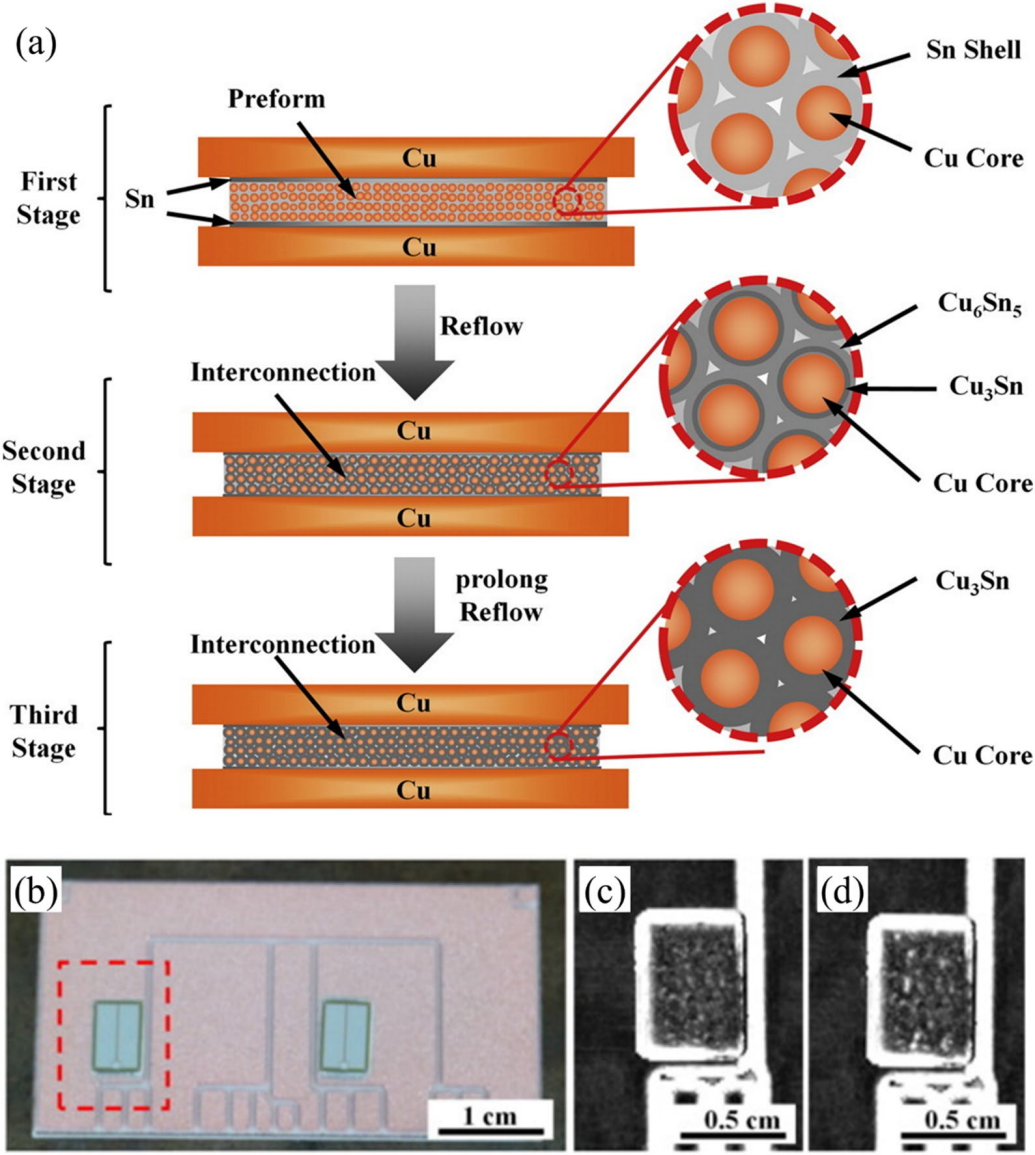
**a** Schematic diagram of the bonding process for the high-temperature shearing sample based on a preform fabricated with Cu@Sn particles, **b** Silicon IGBT power devices bonded onto a DBC using the Cu@Sn preform, **c** SAM image of the interconnect, and **d** SAM image of the interconnection after thermal cycling. [Source [95]: Reprinted from Materials & Design, Vol. 108, Hu, T., Chen, H., Li, M., "Die attach materials with high remelting temperatures created by bonding Cu@Sn microparticles at lower temperatures", pp. 383–390, Copyright (2016), with permission from Elsevier.]

Hu and colleagues [95] presented an innovative die attach solution based on Cu@Sn core–shell microparticles that addressed a critical challenge in WBG device packaging: high temperature stability with conventional processing compatibility. The particles, consisting of copper cores (30 μm diameter) with a 3**–**5 μm tin shell, transformed during reflow at 250 °C into a robust layer capable of withstanding temperatures up to 676 °C. As shown in Fig. 15a, the bonding process that transformed the microstructure occurred in three stages. Initially, the Sn shell melted during the reflow process and connected the Cu cores. Subsequently, the structure progressively transformed into network-like Cu_6_Sn_5_ and Cu_3_Sn IMCs, created a skeleton-shaped structure in which the copper cores were interconnected by thermally stable IMC bridges.

The thermal cycling reliability of this approach is particularly outstanding. The researchers subjected samples to thermal cycling between − 55 and 200 °C for 500 cycles. Figure 15b shows silicon insulated gate bipolar transistor (IGBT) power devices bonded onto a direct bonded copper (DBC) substrate using the Cu@Sn preform, with corresponding scanning acoustic microscopy (SAM) images before and after thermal cycling (Fig. 15c, d). The samples preserved their complete bond structure across the entire interface without voids or delamination. This exceptional reliability results from several complementary factors. First, the elimination of low-melting-point phases prevented typical failure modes associated with conventional solders. Second, the network-like arrangement of Cu cores distributed thermal stress uniformly throughout the joint structure. Finally, the 400 μm bond line effectively absorbed thermo-mechanical stresses arising from the CTE mismatch between the chip and substrate. Unlike thin transient liquid phase bond lines that are prone to cracking during thermal cycling, this thicker bond line provided superior mechanical resilience. These combined properties make Cu@Sn core–shell microparticle technology an excellent die attach solution for next-generation WBG power devices operating in harsh thermal environments.

The advanced nanocomposite solder architectures discussed in this section demonstrate significant progress in addressing the challenges of WBG semiconductor packaging through innovative material designs. Carbon-based nanocomposites provide enhanced thermal conductivity and mechanical resilience, TLPS systems enable high-temperature stability through strategic intermetallic formation, and core–shell nanostructures offer controlled processing with superior joint properties. However, despite these advances, solder-based approaches still face fundamental limitations when operating at extreme temperatures (exceeding 300 °C) and demanding reliability requirements of next-generation WBG devices. The inherent melting point constraints of solder alloys create an upper bound on operational temperature capabilities. Furthermore, the complexity of multi-component systems and the potential for IMCs degradation mechanisms remain concerns for long-term reliability. These limitations have driven researchers to explore alternative die attach methodologies that can overcome the temperature boundaries. Consequently, nano-sintering technologies have emerged as a paradigm-shifting solution, offering the potential for operation at temperatures approaching the melting point of pure metals while maintaining processing compatibility with semiconductor manufacturing. The following section examines how nanoscale sintering approaches address these fundamental challenges through entirely different bonding mechanisms.

## Nanocomposite sintering for die attach

### Fundamentals of nanoscale sintering

The transition from conventional soldering to sintering technologies for die attachment in WBG power semiconductor packages has been driven primarily by the thermal limitations of traditional solder materials. Because WBG semiconductors operate at temperatures exceeding 200 °C, conventional solder joints experience accelerated degradation through IMCs growth, creep, and thermomechanical fatigue [96, 97]. Even solders for high temperature applications (Au or Ag) face challenges with poor wettability, high cost, or insufficient reliability in thermal cycling conditions [98]. Furthermore, the CTE mismatch between WBG semiconductor dies and substrate materials generates substantial stress at the die attach interface during temperature fluctuations, leading to premature failure of conventional solder joints through crack propagation and delamination. These fundamental limitations have driven the development of alternative joining technologies than can withstand the extreme operating conditions of WBG devices.

Sintering technology represents a paradigm shift in die attachment methodology by creating bonds through diffusion and neck formation between adjacent particles without the need for a complete melting phase [99]. The process typically uses metal nanoparticles with dramatically reduced melting points, compared with their bulk counterparts, due to the thermodynamic size effect [100]. When these nanoparticles are heated to moderate temperatures (typically 100–300 °C) [45, 101], they undergo solid-state diffusion and eventually form a continuous interconnected network with high thermal stability. Sintered joints offer significant advantages over conventional soldered joints. While soldered joints rely on alloying and IMC formation, sintered joints maintain much higher remelting temperatures that often approach those of the bulk metal. Additionally, sintered joints demonstrate superior resistance to thermomechanical fatigue. These characteristics enable sintered joints to maintain their structural and electrical integrity throughout the operational lifetime of WBG devices, even under extreme temperature fluctuations [97].

**Fig. 16:**
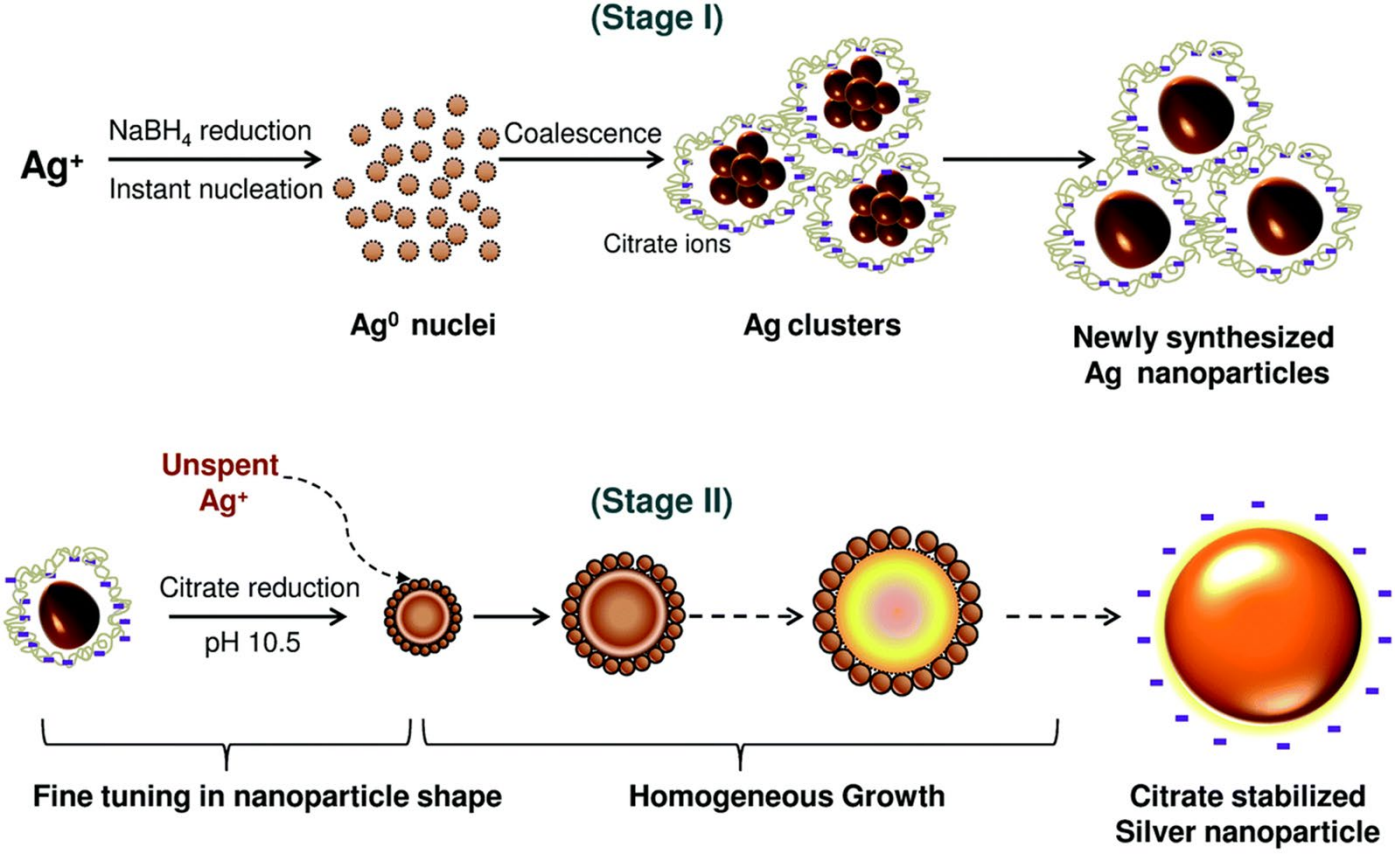
Schematic image of a silver nanoparticle synthesis process. [Source [105]: S. Agnihotri, S. Mukherji, and S. Mukherji, "Size-controlled silver nanoparticles synthesized over the range 5–100 nm using the same protocol and their antibacterial efficacy", RSC Advances, Vol. 4(8), pp. 3974–3983, 2014. © 2014 The Royal Society of Chemistry. This article is licensed under a Creative Commons Attribution 3.0 Unported License.]

Nanoparticles for sintering applications can be synthesized by various methods, including citrate-only reduction (e.g., the Turkevich method), polyol-based synthesis, and a multi-step process involving seed formation with sodium borohydride (NaBH_4_) followed by secondary growth [102–104]. Figure 16 shows the typical two-stage silver nanoparticle synthesis process described by Agnihotri et al. [105]. In Stage I, silver ions (Ag⁺) undergo reduction using NaBH_4_ to form Ag^0^ nuclei, which subsequently coalesce into silver clusters stabilized by citrate ions. In Stage II, further refinement occurs through citrate reduction at elevated pH levels (pH 10.5), allowing fine-tuning of the nanoparticle shape followed by homogeneous growth. This precise control over the nanoparticle characteristics is crucial for optimizing sintering behavior, because the particle size and surface chemistry significantly influence the sintering temperature, kinetics, and resulting microstructure.

**Fig. 17:**
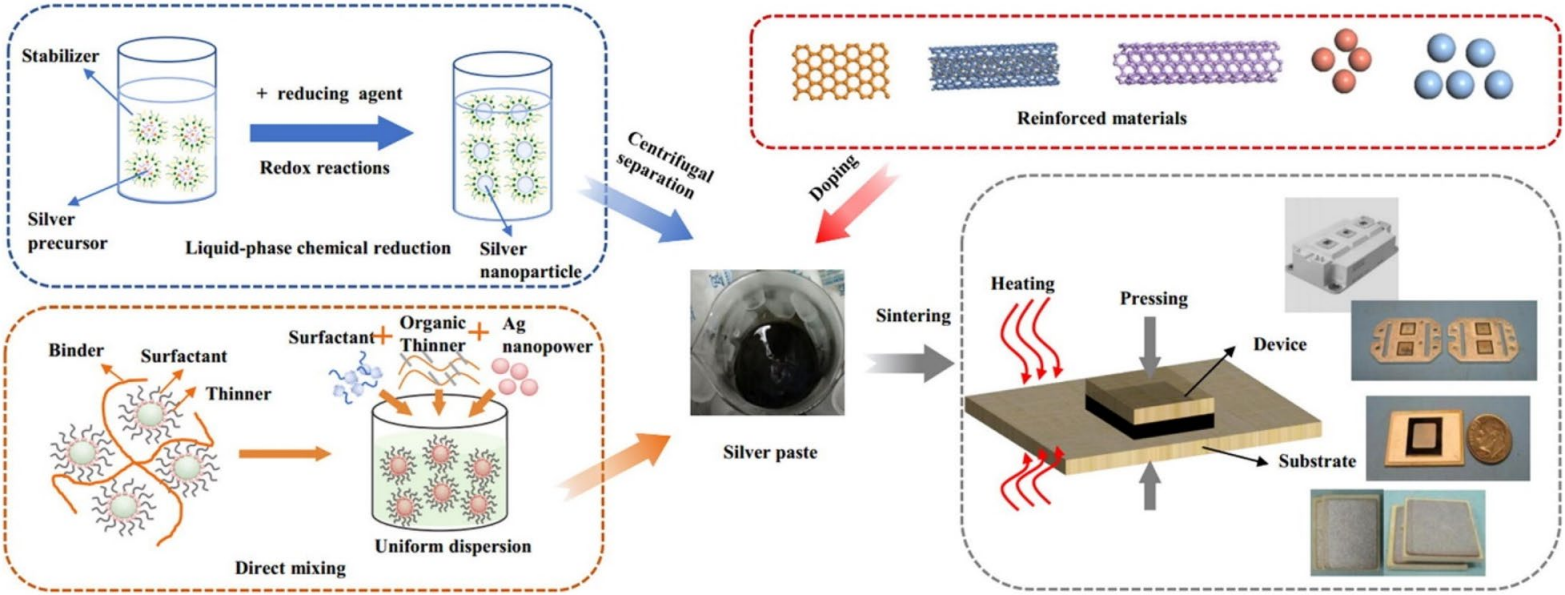
The formulation and processing of nano-Ag paste. [Source [106]: Reprinted from International Journal of Heat and Mass Transfer, Vol. 127, Zhang, P., Jiang, X., Yuan, P. et al., "Silver nanopaste: Synthesis, reinforcements and application", pp. 1048–1069, Copyright (2018), with permission from Elsevier.]

The preparation of silver sinter paste for die attach applications involves multiple processing steps, as shown in Fig. 17 [106]. Silver nanoparticle paste preparation typically begins with a liquid-phase chemical reduction of silver precursors in the presence of stabilizers. These nanoparticles are then separated through centrifugation and incorporated into a formulated paste system. The paste formulation process involves directly mixing binders, surfactants, organic thinners, and the silver nano powder to achieve a uniform dispersion with optimized rheological properties. The final paste can be applied through various deposition methods, including screen printing, stencil printing, or dispensing. The sintering parameters, including temperature, pressure (if applied), atmosphere, and time, must be precisely controlled to achieve optimal densification without damaging the die or substrate. Additionally, reinforcement materials such as graphene [107–109], CNTs [110, 111], or ceramic particles (SiC, AIN, and Al_2_O_3_) [112–115] can be incorporated to enhance the mechanical properties and reliability of the sintered joint. This comprehensive approach to silver paste preparation enables the production of high-performance die attach materials with properties tailored for specific WBG device requirements.

Optimal sintered joints must simultaneously demonstrate high thermal conductivity to efficiently dissipate heat from WBG devices, excellent electrical conductivity to minimize power losses, high mechanical strength to withstand thermomechanical stresses, and long-term stability at operating temperatures up to 300 °C [116]. Additionally, the sintering paste formulation should meet several critical requirements. First, it requires rheological properties appropriate for consistent deposition during manufacturing. The particle size distribution, typically ranging from 20–100 nm, must be suitable for facilitating neck formation at moderate processing temperatures. Furthermore, controlled surface chemistry is essential to prevent premature agglomeration of particles. Finally, minimal organic content is necessary to reduce porosity after sintering [117, 118]. The processing parameters, including temperature, pressure, atmosphere, and time, must be precisely controlled to achieve optimal densification without damaging the WBG die or substrate [119]. These demanding requirements have driven significant research to develop various nanoparticle compositions, surface treatments, paste formulations, and processing methodologies for sintering solutions that meet the complex needs of WBG power electronics.

### Nanoparticle sintering technology

Sintering technology uses nanoparticles of materials such as copper, gold, and silver. Copper nanoparticle sintering offers excellent thermal conductivity and low cost but faces challenges with oxidation sensitivity [120, 121]. Gold nanoparticle sintering provides exceptional reliability and oxidation resistance but at significantly higher material costs [122]. Hybrid systems combining nanoparticles with microparticles [123, 124] or flakes [125–127] have also been explored to optimize processing conditions and physical properties. Among those various options, silver nanoparticle sintering has attracted the most research and commercial attention due to its excellent thermal conductivity (bulk Ag: 429 W m^−1^ K^−1^) [128], superior electrical conductivity (bulk Ag: 1.59 μΩ cm) [129], and high melting point (bulk Ag: 961 °C) [130].

In this section, we comprehensively analyze the fundamental principles of nanoscale sintering technology, exploring how the unique properties of nanoparticles enable low-temperature processing while achieving high-temperature stability. We examine various nanoparticle sintering approaches including pressure-assisted and pressure-less techniques, along with advanced nanocomposite sintering systems that incorporate reinforcement materials.

**Fig. 18:**
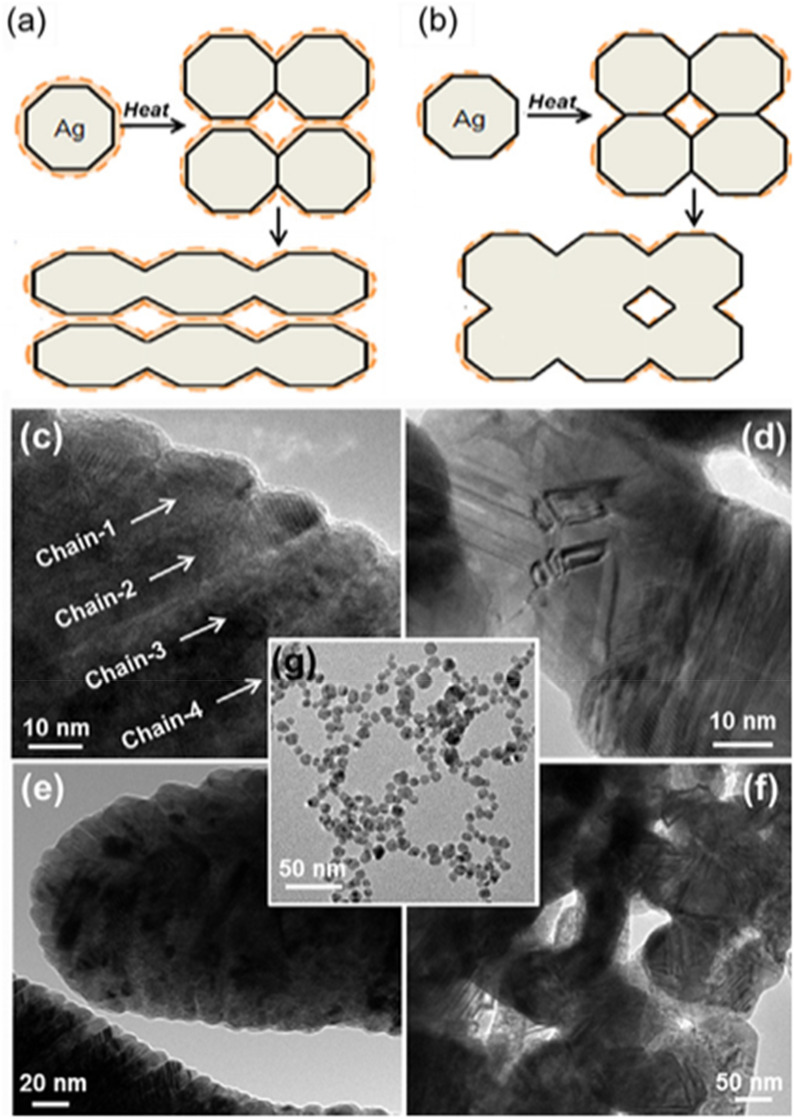
Schematic diagrams of the sintering process for Ag nanoparticles with **a** Thick organic shells and **b** Thin organic shells. TEM images of Ag nanoparticles sintered at 200 °C for 20 min with **c** Thick organic shells and **d** Thin organic shells. **e, f** are low magnification TEM images of **c** and **d**, respectively. **g** TEM image of the initial Ag nanoparticles. [Source [131]: Reprinted from Scripta Materialia, Vol. 69, No. 11-12, Wang, S., Li, M., Ji, H. et al., "Rapid pressureless low-temperature sintering of Ag nanoparticles for high-power density electronic packaging", pp. 789–792, Copyright(2013), with permission from Elsevier.]

Wang et al. [131] specifically investigated how controlling the organic shell thickness around silver nanoparticles influences the sintering behavior and resulting properties. As shown in Fig. 18a, the process of sintering Ag nanoparticles follows different pathways depending on the thickness of the organic shells on their surfaces. With thick organic shells (Fig. 18a), Ag nanoparticles have fewer contact points, resulting in the chain-like sintered structures shown in Fig. 18c, e. In contrast, Ag nanoparticles with thin organic shells form more extensive interparticle connections, creating the net-like sintered morphology shown in Fig. 18d, f. That structure enables more efficient densification during the sintering process. For comparison, Fig. 18g shows the initial Ag nanoparticles before sintering. Wang et al. reported that the thermal conductivity of sintered silver with thin organic shells increases dramatically with the sintering temperature, reaching values of 229 W m^−1^ K^−1^ at 200 °C while maintaining relatively low porosity (approximately 27%). This conductivity is significantly higher than that achieved with thick organic shells (74 W m^−1^ K^−1^) and conventional solder materials.

**Fig. 19:**
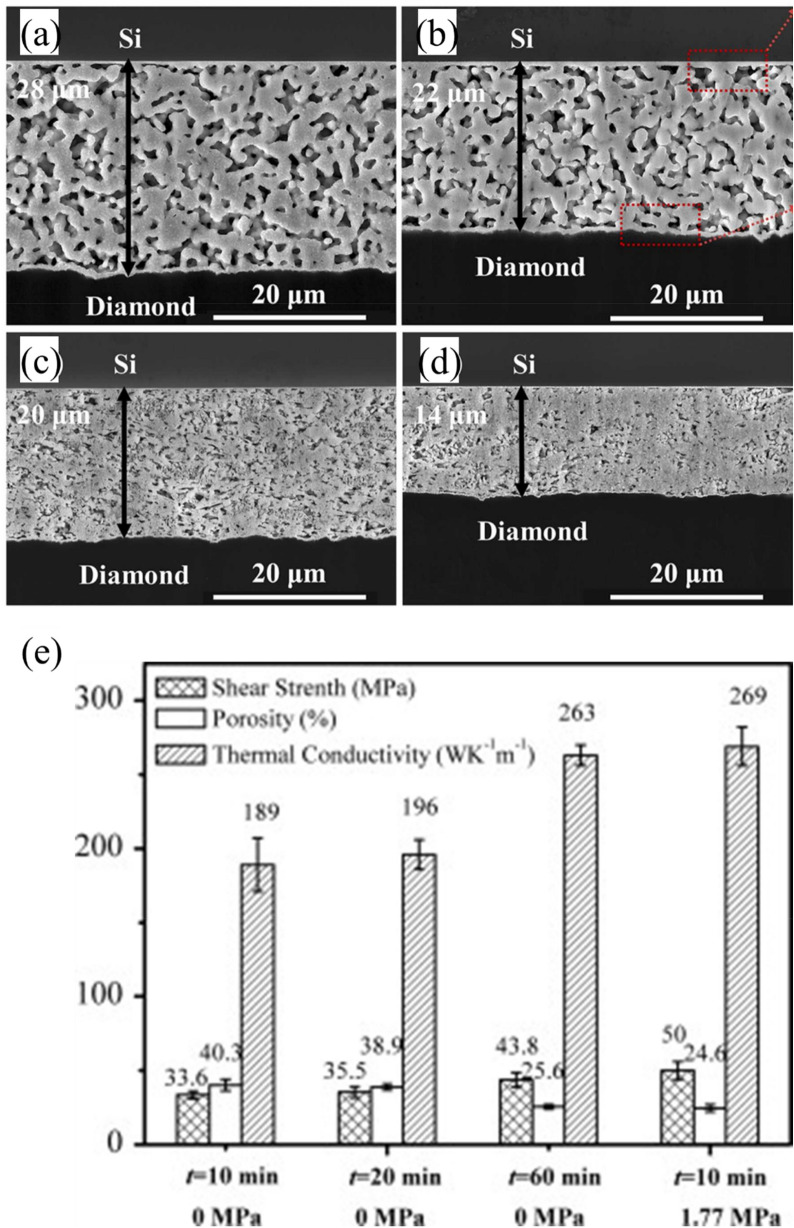
Cross-sectional SEM images of the Si/diamond bonding interfaces under sintering pressure of **a** 2, **b** 5, **c** 7, and **d** 10 MPa. [Source [133]: Reprinted from Materials Today Communications, Vol. 34, Zhao, K., Zhao, J., Wei, X. et al., "Mechanical properties and microstructure of large-area diamond/silicon bonds formed by pressure-assisted silver sintering for thermal management", 105230, Copyright (2023), with permission from Elsevier.] **e** Average shear strength, porosity and thermal conductivity of specimens made using different combinations of holding time and pressure. [Source [134]: Reprinted from Materials Letters, Vol. 128, Fu, S., Mei, Y., Lu, G.-Q. et al., "Pressureless sintering of nanosilver paste at low temperature to join large area (≥ 100 mm^2^) power chips for electronic packaging", pp. 42–45, Copyright (2014), with permission from Elsevier.]

External pressure and sintering time are important factors in achieving stable die attachment. Yu et al. [132] investigated sintering at temperatures up to 300 °C at various pressures, pressure-less bonding (~ 0 MPa) and moderate pressures up to ~ 7.6 MPa. They reported that the porosity could be reduced from 15–17% to 30–40% when moderate pressure (7.6 MPa) was applied. This reduction in porosity increased the shear strengths to more than 50 MPa and enhanced joint reliability. The combined heat-and-pressure profile not only effectively removed organic residues but also promoted intimate particle-to-particle contact, resulting in a denser and more uniform microstructure. Similarly, Zhao et al. [133] investigated Si chip/diamond heat spreader systems using pressure-assisted Ag sintering at 220 °C. As shown in Fig. 19a–d, the thickness of the bonding layer decreased from 28 to 14 *µ*m, and the porosity decreased from 22.87 to 9.54% as the pressure increased from 2 to 10 MPa. The reduction in interfacial thickness, porosity, and pore size at the same sintering temperature reveals that higher pressure facilitates the densification of the bonding layer. These cross-sectional SEM analyses clearly demonstrate that higher bonding pressure promotes intimate particle-to-particle contact, resulting in denser and more uniform microstructures essential for reliable die attachment in WBG power semiconductor applications.

Despite the advantages of pressure-assisted sintering, industrial applications ultimately require pressure-less bonding processes for practical manufacturing considerations, particularly when dealing with large-area dies or complex multi-die modules. Therefore, Fu et al. [134] studied large area die attach bonding (≥ 100 mm^2^) using a specially formulated nano-silver paste that mixed silver nanoparticles with larger microparticles for pressure-less bonding. Despite applying little to no external force, they achieved high-performance joints with high shear strengths (43.8 MPa), low porosity (25.6%), and high thermal conductivity (263 W m^−1^ K^−1^). Additionally, even minimal pressure (1.77 MPa) further improved densification, increasing shear strength to approximately 50 MPa and lowering porosity below 25% (Fig. 19e). The excellent performance of these pressure-less joints can be attributed to the size-dependent diffusion behavior of nanoparticles. Small nanoparticles with high surface energy actively diffuse and fill the gaps around large microparticles, promoting extensive neck formation and reducing the formation of large voids. In addition, the precise temperature profile (gradual ramping to 250 °C) ensures the consistent removal of organic components, enabling the particles to rearrange and sinter into a contiguous metallic network without requiring high compressive forces. This approach allowed large-area chips to be robustly bonded with minimal porosity while maintaining excellent mechanical and thermal properties. The research clearly demonstrates that nanomaterials play a pivotal role in high reliability die attach technologies, particularly for WBG devices that must withstand extreme thermal and power-cycling demands.

**Fig. 20:**
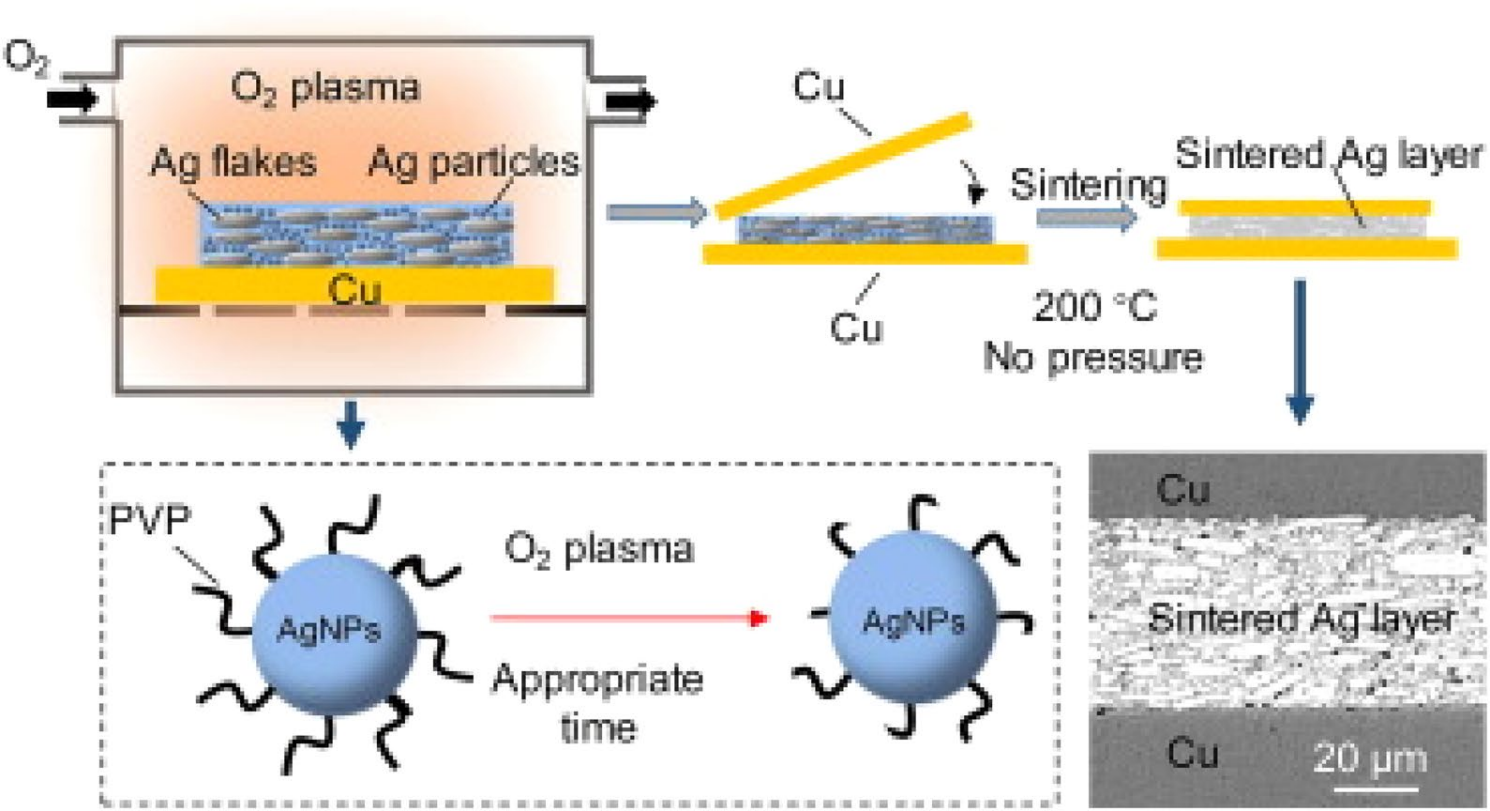
Schematic image of a controllable plasma-activated method for pressure-less sintering of Ag nanoparticles [Source [135]: Reprinted from Materials Letters, Vol. 256, H. Fang, C. Wang, T. Wang et al., "Pressureless low-temperature sintering of plasma activated Ag nanoparticles for high-power device packaging", 126620, Copyright (2019), with permission from Elsevier.]

As another approach to pressure-less sintering, Fang et al. [135] studied an innovative oxygen plasma activation method. As shown in Fig. 20, this approach treated a composite silver paste containing both Ag flakes and nanoparticles with oxygen plasma to effectively remove the organic shells (primarily PVP) surrounding the silver nanoparticles. The surface modification facilitates efficient sintering at a relatively low temperature (200 °C) without requiring external pressure. A cross-sectional SEM analysis confirmed the formation of a uniform sintered silver layer between copper substrates. The researchers systematically evaluated plasma treatment parameters. The activation depth increased linearly with plasma output power. Different properties showed distinct optimal treatment times: thermal conductivity peaked at 5 s, while shear strength maximized at 10 s with the added benefit of reduced porosity. This plasma activation technique represents a significant advancement in pressure-less silver sintering technology for high-performance die attach materials.

### Enhanced nanocomposite sintering systems

Although pure Ag NP sintering offers excellent performance, researchers have developed various nanocomposite sintering systems to further enhance specific properties or address challenges in WBG power semiconductor packaging applications. These approaches typically involve combining AgNPs with other nanomaterials to create synergistic effects that overcome the limitations of single-material systems. Several key factors drive these developments: (1) improved thermal conductivity for efficient heat dissipation from WBG devices operating at elevated temperatures (200–300 °C); (2) enhanced mechanical strength and reliability to withstand severe thermomechanical stress cycling; (3) reduced processing temperatures or pressures to ensure manufacturing compatibility with temperature-sensitive components; and (4) optimized electrical performance to minimize power losses in high-density applications.

In the following sections, we introduce several advanced nanocomposite sintering approaches: metal nanowire reinforcement [136], bimodal particle size distribution systems [137], Cu–Ag composite structures [138], and silicon-containing composites [139]. Each approach offers distinct advantages for meeting the demanding performance requirements of next-generation WBG power electronic devices.

#### Nanowire reinforcement

**Fig. 21:**
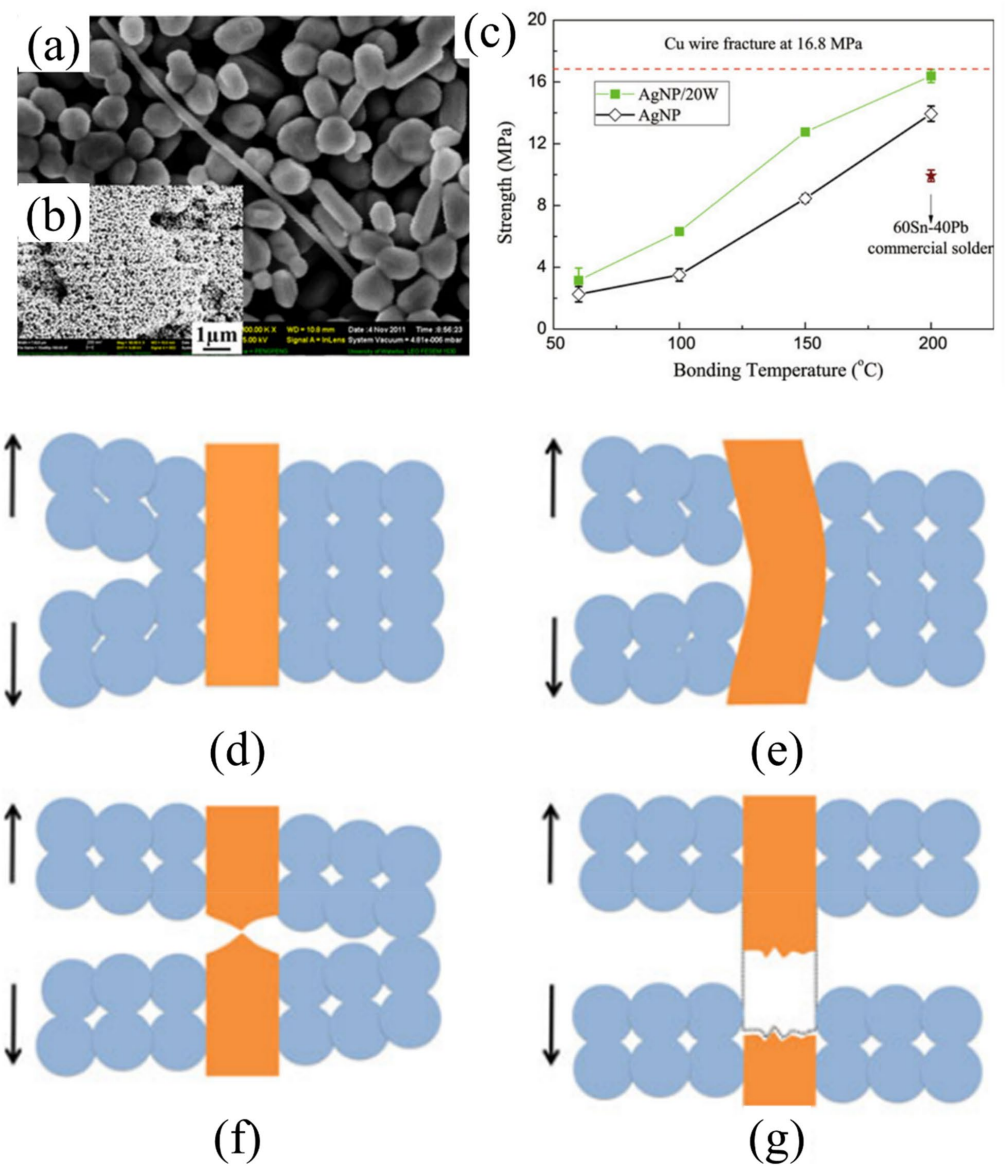
**a** SEM images of sintered paste with Ag NW added at 10 vol.% (**b**: low magnification image), **c** Bond strength of AgNP/20NW and AgNP pastes as a function of the bonding temperature. The star represents a 60Sn–40%Pb commercial solder joint soldered at 200°C. Schematic mechanisms of Ag NW reinforcement in Ag NP/NW joints: **d** Energy absorption causes crack closure by NP–NP debonding and crack propagation (NP–NP bond\stress on crack tip), **e** While the crack grows in front of the Ag NWs, the deformation splits along the NWs and forms compressive stress that bends the NWs. The dispersed strain and energy consumption of bending the NWs stops the crack propagation or causes crack deflection (stress on crack tip\NW yield strength\NP–NW bond). **f** Deformation or breaking of the NWs (NW-yield strength\stress on crack tip\NP–NW bond), **g** NW being pulled-out by debonding of NP–NW and further diversion of the crack (NP–NW bond\stress on crack tip). The newly generated smaller crack can propagate in the debonded areas. [Source [136]: P. Peng, A. Hu, B. Zhao, A. P. Gerlich, and Y. N. Zhou, "Reinforcement of Ag nanoparticle paste with nanowires for low temperature pressureless bonding", Journal of Materials Science, Vol. 47(19), pp. 6801-6811, 2012. Reproduced with permission from SNCSC.]

Nanowire reinforcement has emerged as a promising way to enhance the mechanical and electrical properties of die attach materials for power semiconductor packaging. By incorporating nanowires into pastes, many researchers have developed composite bonding materials with better strength, reliability, and thermomechanical performance than conventional die attach materials [136, 140, 141]. For example, Peng et al. [136] conducted a comprehensive investigation of silver nanowire (AgNW) reinforcement in silver nanoparticle (AgNP) matrices. As illustrated in Fig. 21a, b, the resulting microstructure exhibited spherical Ag nanoparticles effectively strengthened by elongated Ag nanowires, creating a distinctive composite architecture. Mechanical characterization (Fig. 21c) demonstrated that the Ag NP/NW composites (AgNP/20W) consistently outperformed pure AgNP joints across the entire bonding temperature range from 60 to 200 °C. At a bonding temperature of 200 °C, the nanowire-reinforced composite achieved a bond strength of approximately 16 MPa. This strength is comparable to the fracture strength of Cu wire (16.8 MPa). Furthermore, it significantly exceeds the strength of pure AgNP joints (13.5 MPa) and commercial 60Sn–40Pb solder (10 MPa).This strength enhancement becomes particularly pronounced at lower processing temperatures, with the reinforced composite delivering nearly twice the strength of pure AgNP solder at 100 and 150 °C.

The toughening mechanisms are clearly illustrated in Fig. 21d–g, which reveals a sequential process in which nanowires bridge developing cracks (e), undergo controlled stretching (f), and ultimately experience breakage or pull-out (g) during progressive failure. When a crack begins in the AgNP matrix, the nanowires act as bridges that span across the crack faces, effectively restricting crack opening displacement and transferring load between disconnected regions. As crack propagation continues, the high-aspect-ratio nanowires undergo elastic and plastic deformation, accommodating substantial strain energy before reaching critical thresholds. The nanowires ultimately fail through either necking and fracture mechanisms or gradual pull-out from the surrounding matrix, with both processes dissipating considerable energy through plastic work and frictional resistance. According to the research results, the nanowire-reinforced composite paste exhibited fracture energy values up to three times higher than those of conventional AgNP joints, despite showing similar maximum failure loads. Such enhanced energy absorption capabilities make these nanowire-reinforced materials exceptionally well-suited for WBG power semiconductor packaging applications that experience severe thermal cycling, mechanical vibration, and impact loading conditions.

#### Bimodal particle size distribution

The process of sintering Cu nanoparticles presents significant challenges due to oxidation and porosity formation in the die attach layer. In particular, small nanoparticles exhibit enhanced sintering activity due to their high surface energy, but they frequently cause oxidation issues and undesirable pore structures. In contrast, larger particles have high oxidation resistance, but they require elevated sintering temperatures or pressures [101]. Therefore, the bimodal particle size distribution strategy has emerged as an effective solution. It has been reported that when two types of spheres with different diameters are arranged together, the smaller particles can occupy the interstitial spaces created by the larger particles, thus increasing particle contact and enhancing overall packing density [142, 143].

**Fig. 22:**
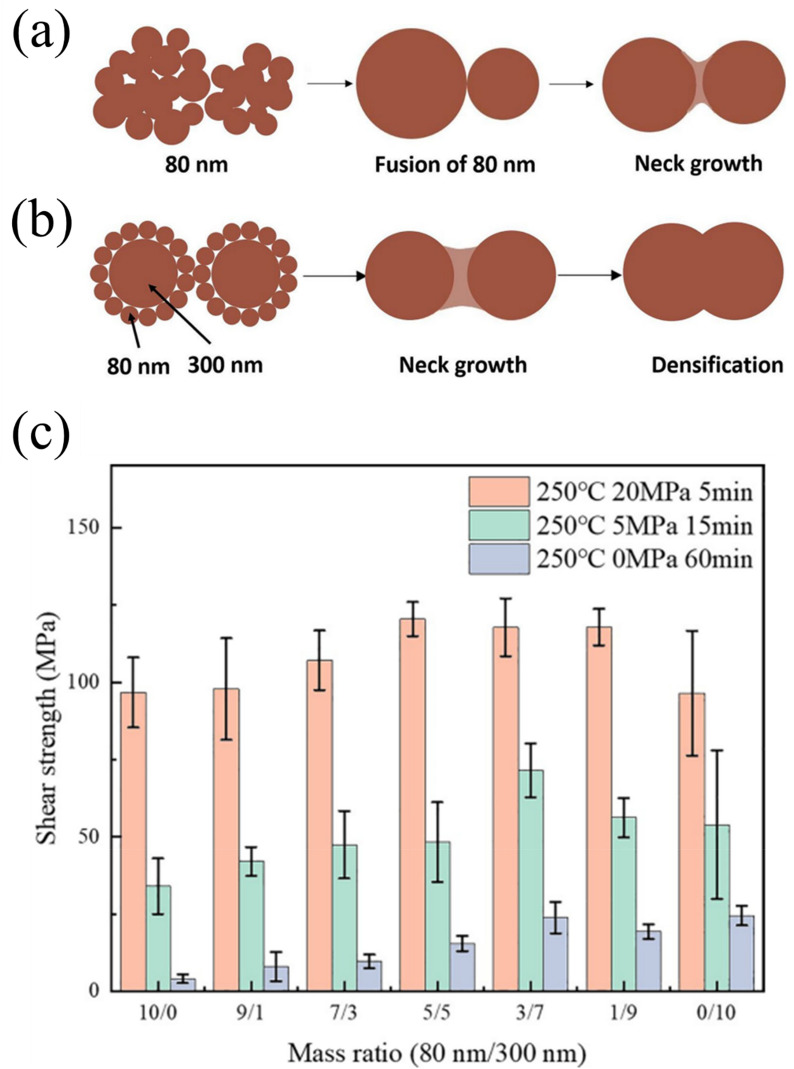
Schematic of the sintering mechanism: **a** Pure-size Cu nanoparticle paste and **b** Bimodal-sized Cu nanoparticle paste, **c** Shear strength of Cu–Cu joints with different composite pastes sintered in a formic acid atmosphere [Source [137]: L. Ma, Z. Lu, Q. Jia, Z. Cui, Y. Wang, D. Li, H. Zhang, G. Zou, and F. Guo, "Sintering Mechanism of Bimodal-Sized Cu Nanoparticle Paste for Power Electronics Packaging", Journal of Electronic Materials, Vol. 53(6), pp. 2988-2998, 2024. Reproduced with permission from SNCSC.]

Ma et al. [137] investigated this approach by developing Cu nanoparticle pastes combining 80 and 300 nm particles in various ratios. As shown in Fig. 22a, b, the sintering mechanisms differed significantly between mono-sized and bimodal particle distributions. In the mono-sized system (Fig. 22a), individual 80-nm particles fused together and formed neck structures through surface diffusion and particle coalescence. On the other hand, the bimodal system (Fig. 22b) demonstrated superior behavior, with the 80-nm particles fill the voids between the 300-nm particles, enabling more efficient neck growth and subsequent densification. The effectiveness of this gap-filling mechanism closely relates to the optimal ratio between particles with small and large dimensions. Their systematic study revealed that a mass ratio of 3:7 (80:300 nm) provided optimal performance through ideal packing density and network formation. At that ratio, the larger particles formed a structural backbone. Meanwhile, the smaller particles with high surface activity effectively bridged connections between them. This arrangement created an interconnected mesh-like structure. They also reported that deviating from that optimal ratio led to either localized agglomeration (with pure 80 nm particles) or limited neck formation and poor network development (with pure 300 nm particles).

The enhanced densification and interconnected network structure of the bimodal system resulted in superior mechanical performance, as demonstrated in Fig. 22c. At 250 °C with 20 MPa of pressure applied for 5 min, the 3:7 bimodal mixture achieved the exceptional shear strength of 102.46 MPa, significantly outperforming both mono-sized compositions. In addition, the bimodal sintered joints had excellent thermal conductivity (284 W m^−1^ K^−1^), low electrical resistivity (4.42 μΩ cm), and minimal porosity (11.78%). This bimodal approach is particularly valuable for Cu-based sintering systems, in which rapid densification helps minimize oxidation during the bonding process while maintaining the excellent thermomechanical properties required for WBG semiconductor packaging applications.

#### Cu–Ag composite systems

Although pure Ag nanoparticles offer excellent sintering properties, their high cost and insufficient mechanical reliability limit their widespread adoption in WBG power semiconductor packaging. Similarly, Cu nanoparticles provide cost-effectiveness, but they suffer from oxidation issues and require higher sintering temperatures. Cu–Ag composite nanoparticle systems have emerged as a promising solution that combines the cost advantage of Cu with the superior sintering characteristics of Ag [144–146].

**Fig. 23:**
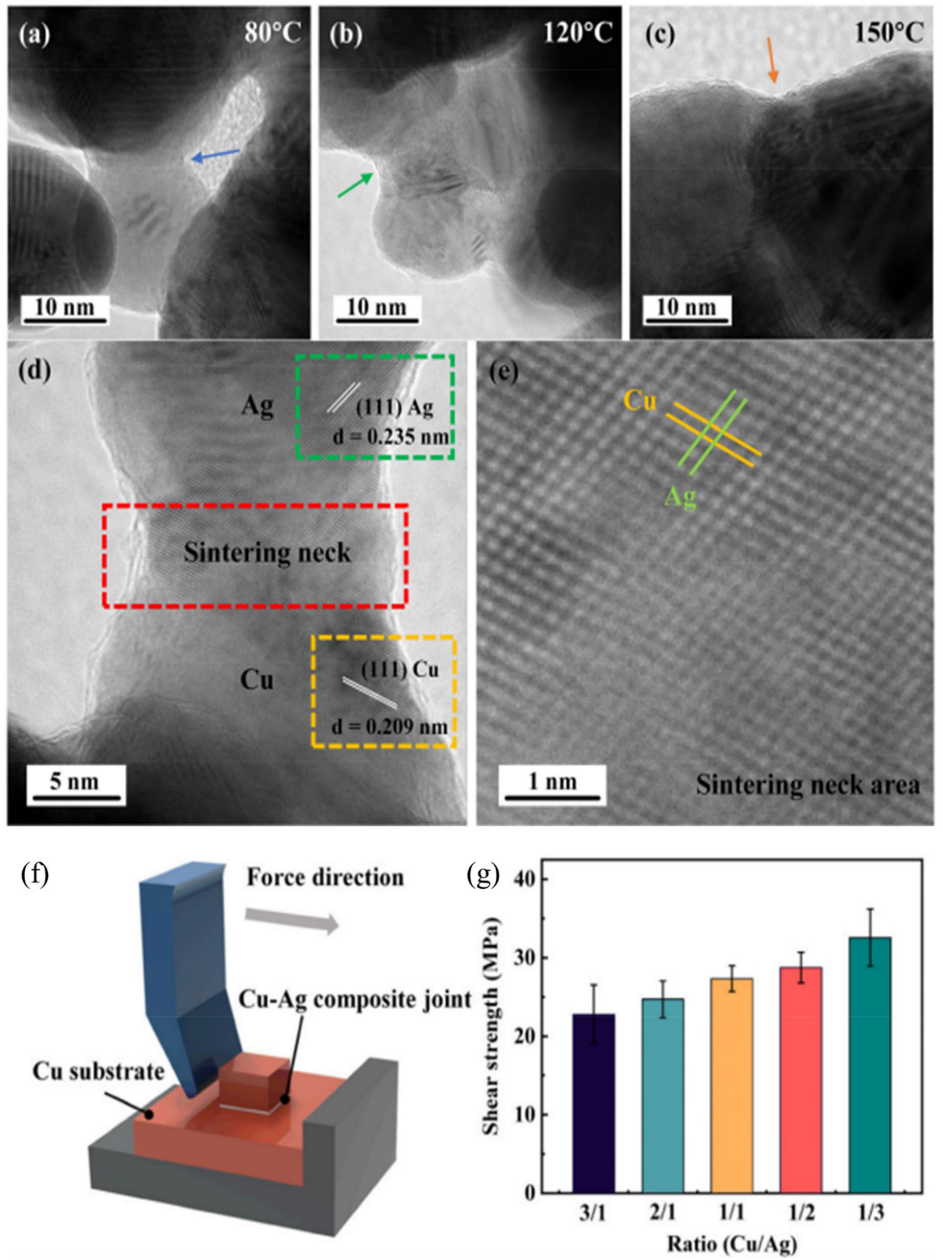
**a–c** TEM images of sintering at different temperatures, **d** HRTEM image of sintering at 100°C, **e** Red area in (**d**), **f** Diagram for the shear test, **g** Shear strength of joints with different Cu and Ag ratios. [Source [138]: Reprinted with permission from IEEE Transactions on Electron Devices. W. Lv, J. Liu, Y. Mou, Y. Ding, M. Chen, and F. Zhu, "Fabrication and Sintering Behavior of Nano Cu-Ag Composite Paste for High-Power Device", IEEE Transactions on Electron Devices, Vol. 70, No. 6, pp. 3202-3207, 2023. © 2023 IEEE.]

Lv et al. [138] investigated the sintering behavior of Cu–Ag composite nanoparticles with different Cu ratios (Cu_3_Ag_1_, Cu_2_Ag_1_, Cu_1_Ag_1_, Cu_1_Ag_2_, Cu_1_Ag_3_). Figure 23a–c shows transmission electron microscopy (TEM) images of sintering at different temperatures. The adjacent nanoparticles were initially connected at 80 °C, and a stable sintering neck was formed between Cu and AgNPs, as shown in Fig. 23a. As the sintering temperature increased, the sintering neck expanded rapidly, and the NPs were sufficiently sintered. The width of the sintering neck was almost equal to the particle diameter when sintering occurred at 150 °C, and the composite NPs achieved a stable interconnection, as shown in Fig. 23c. A high-resolution TEM image of Cu–Ag composite NPs sintered at 100 °C are shown in Fig. 23d. The (111) crystal planes of the Cu and Ag NPs are marked by white lines, and the interplanar distances were 0.209 and 0.235 nm, respectively. The particles are closely connected without any visual void. In addition, the superposition and interference of the Cu and Ag lattice cause many moiré stripes in the sintering neck area, indicating that a compact sintering neck was formed between two the NPs, as described in Fig. 23e.

Figure 23f shows the shear force test diagram used to measure the shear strengths of the nano Cu-Ag composite paste bonding joints after sintering. Figure 23g shows the shear strength results for the joints. The shear strength of the joint increased from 22.8 to 32.6 MPa as the Ag ratio increased. Molecular dynamics simulations support the experimental trends. The Cu1Ag3 composite system reached first contact at only 420 K (146.85 °C) and developed the fastest neck growth among the other ratios tested. This phenomenon deemed to be a consequence of the high surface area of the small Cu particles combined with the lower surface energy and higher diffusivity of Ag.

These findings highlight the value of Cu**–**Ag composites for WBG power semiconductor packaging. The combined system provides both oxidation resistance and cost-effectiveness, with Ag-rich compositions exhibiting excellent electrical conductivity. The ability to tailor the Cu ratio allows designers to optimize the balance between performance requirements and material costs. Additionally, the composite structure creates enough sintering necks and denser enough interconnections to withstand the high-temperature operation (200**–**300 °C) typical of WBG devices.

#### Si-containing composite systems

Incorporating silicon particles into Ag sintering pastes can substantially enhance the thermal cycling reliability. Silicon offers unique advantages for WBG power semiconductor packaging due to its excellent thermal stability, low cost, and notably low CTE of approximately 2.6 ppm K^−1^ [147]. This low CTE value is particularly valuable because it can help address the CTE mismatch between die attach materials and semiconductor chips or ceramic substrates, which typically have CTE values of 4.0 ppm K^−1^ for SiC and 2.3 ppm K^−1^ for Si_3_N_4_ [147].

Liu et al. [139] recently developed an innovative Ag@Si composite sintering strategy using micron-scale Ag-flakes as fillers and micron-scale Si particles as additives. Through low-temperature (250 °C) and pressure-less sintering processes, they achieved an integrated Ag@Si sintering network. TEM observations revealed a potentially slight diffusion of native SiO_2_ on the Si surface toward the Ag, enabling a well-bonded composite structure rather than a simple physical mixture.

**Fig. 24:**
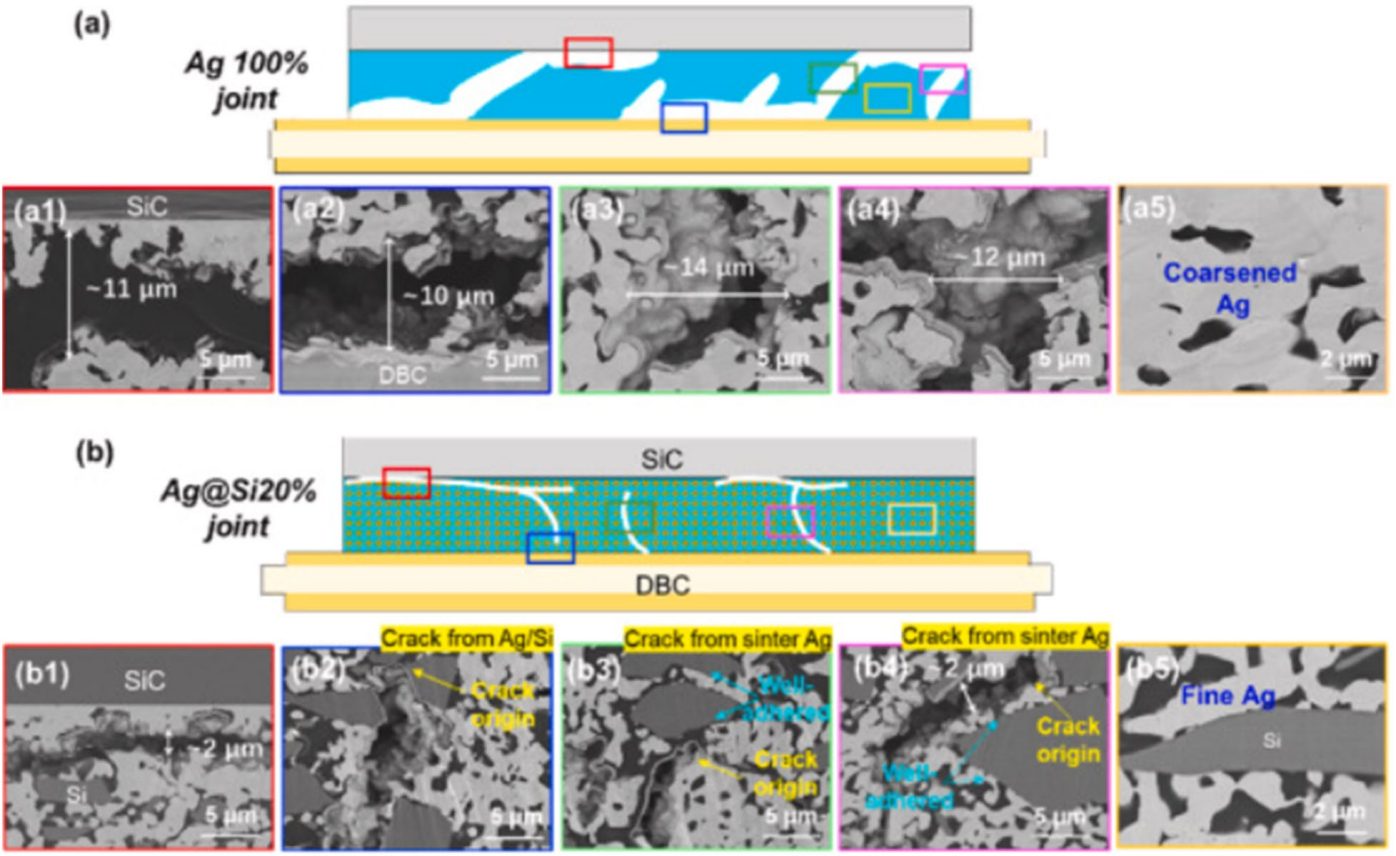
Comparative schematic drawing of the microstructure evolution between **(a)** Ag100% joint and **(b)** Ag@Si20% joint structures after 1000 thermal cycles. (a1-a5) and (b1-b5) are the evolving details at multiple locations within the two joint structures, respectively. [Source [139]: Reprinted from Composites Part B: Engineering, Vol. 281, Y. Liu, C. Chen, Y. Wang et al., "Development of Ag@Si composite sinter joining with ultra-high resistance to thermal shock test for SiC power device: Experiment validation and numerical simulation", 111519, Copyright (2024), with permission from Elsevier.]

To evaluate the effect of Si particles on reliability, the researchers performed a thermal cycling test. A comparative microstructure evolution between pure Ag (Ag100%) and Ag@Si20% joint structures after 1000 thermal cycles from − 50 to 250 °C is shown in Fig. 24a, b. The pure Ag joints exhibited significant degradation during thermal cycling, with large vertical cracks and horizontal delamination exceeding 10 μm in width, leading to poor joint reliability. In contrast, the Ag@Si20% joints maintained their homogeneity and integrity throughout thermal cycling, with only minor deteriorations including a few fine vertical cracks and limited horizontal cracks at the upper bonding interface.

**Fig. 25:**
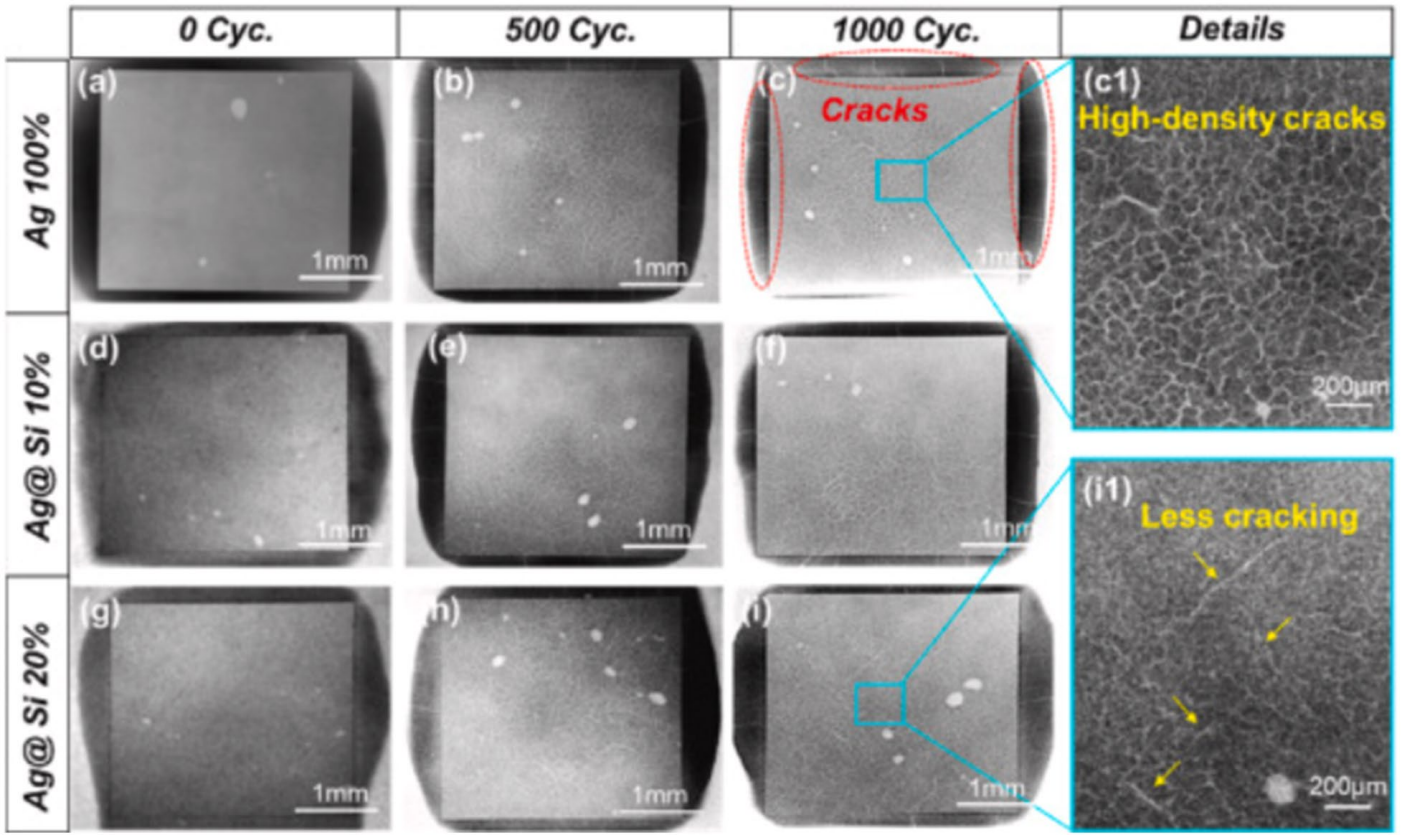
X-ray CT images of three types of die attach structures before thermal cycling and at 500 cycles and 1000 cycles: **a–c** Ag100%, **d–f** Ag@Si10%, **g–i** Ag@Si20%; **(c1)** and **(i1)** are magnified details in the Ag100% and Ag@Si20% joint structures after 1000 thermal cycles. [Source [139]: Reprinted from Composites Part B: Engineering, Vol. 281, Y. Liu, C. Chen, Y. Wang et al., "Development of Ag@Si composite sinter joining with ultra-high resistance to thermal shock test for SiC power device: Experiment validation and numerical simulation", 111519, Copyright (2024), with permission from Elsevier.]

An X-ray computed tomography analysis, shown in Fig. 25, further confirms the superior thermal cycling resistance of the Ag@Si composite joints. After 1000 thermal cycles, the Ag100% joints displayed a dense spiderweb-like pattern of cracks (Fig. 25a–c), but the Ag@Si10% joints showed reduced crack propagation (Fig. 25d–f). Most impressively, the Ag@Si20% joints exhibited significantly fewer cracks, with only sparse defects visible even after 1000 cycles (Fig. 25g–i).

The enhanced thermal reliability of the Ag@Si composite joints can be attributed to several synergistic factors. First, CTE modification plays a crucial role, as the incorporation of 20% Si significantly reduced the CTE value of the die attach material from 16.03 ppm K^−1^ (pure Ag) to 11.08 ppm K^−1^ (Ag@Si20%), resulting in less thermal stress during temperature cycling. Additionally, finite element method simulations revealed improved stress distribution characteristics: the addition of Si led to more uniform stress distribution throughout the joint structure, effectively reducing the edge-concentrated stress that typically initiates crack formation. Furthermore, microstructural stability was enhanced because the Si particles hindered the coarsening of Ag grains during thermal cycling, maintaining the fine structure of the sintered joint and improving resistance to thermal fatigue.

The exceptional performance of the Ag@Si composite joints was reflected in their mechanical reliability. Although the initial shear strength of the Ag@Si20% joints (33.99 MPa) was lower than that of pure Ag joints (60.10 MPa), the Ag@Si20% joints retained 64.93% of their strength after 1000 thermal cycles, compared with only 32.82% for the pure Ag joints. This remarkable retention rate, combined with their lower cost and improved thermal compatibility, makes Ag@Si composite joints a promising solution for high-temperature WBG power semiconductor applications.

### Novel nanomaterial approaches for die attach

Beyond conventional nanoparticle-based systems, researchers have developed innovative nanomaterial approaches to address specific challenges in WBG die attach applications: nanoparticle/solder hybrid joints that combine the advantages of both technologies, dendritic silver nanostructures that create extensive interconnection networks with minimal pressure requirements, and nanoporous metal films that offer organic-free alternatives with excellent thermal stability. These emerging technologies use unique material architectures and nanoscale phenomena to overcome traditional processing limitations while meeting the demanding reliability requirements of high-temperature WBG power semiconductor applications.

#### Nanoparticle/solder hybrid joints

**Fig. 26:**
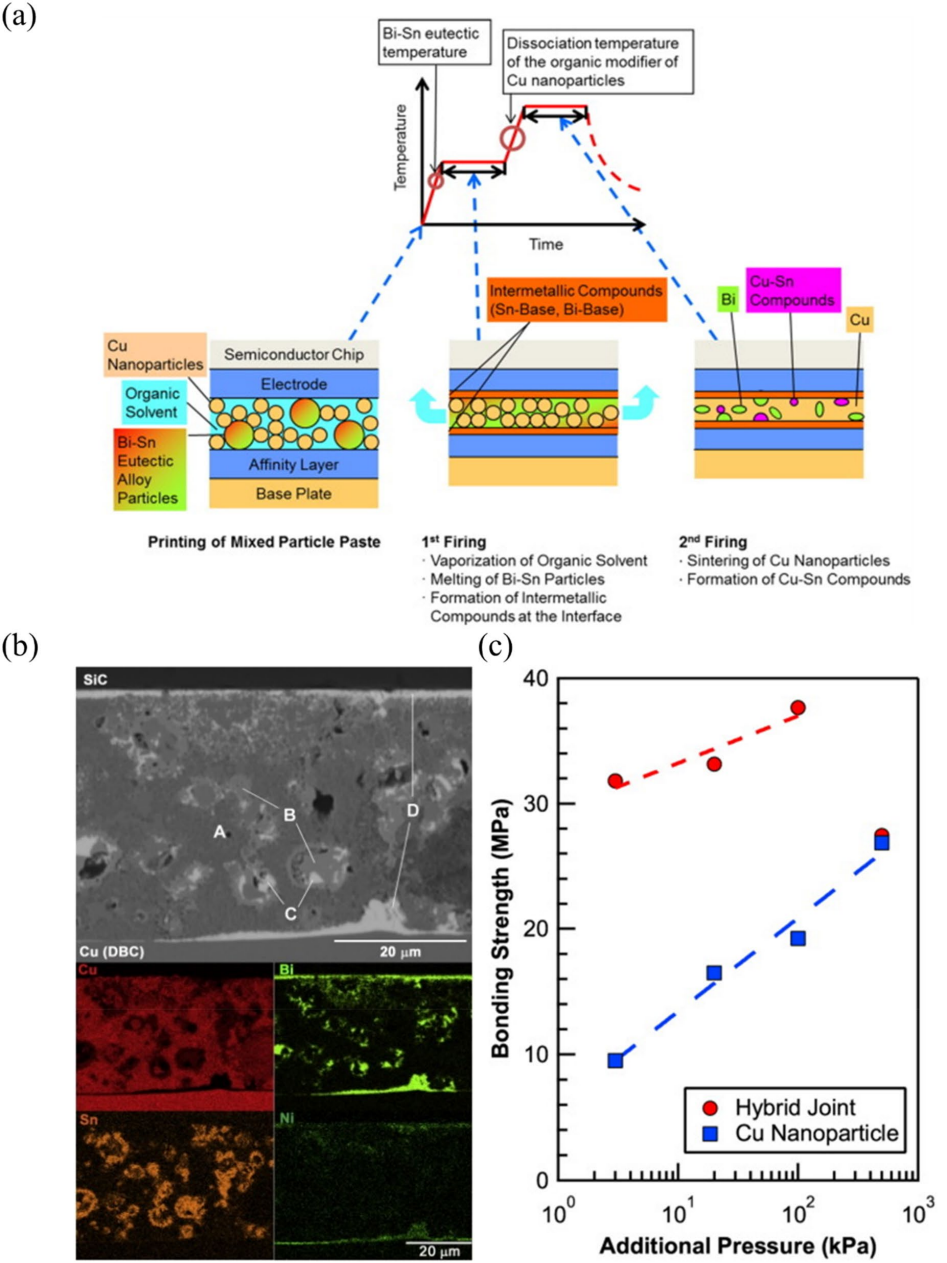
**a** A schematic drawing of the proposed particle/solder hybrid joint method, **b** Cross-sectional SEM and EDS mapping images showing the Cu, Bi, Sn, and Ni phases of the SiC/DBC joint produced by the hybrid joint method, and **c** Dependence of the bonding strength of hybrid SiC/DBC joints and Cu nanoparticle joints on the pressure applied during firing. [Source [148]: Reprinted from Materials & Design, Vol. 124, T. Satoh, T. Ishizaki, and M. Usui, "Nanoparticle/solder hybrid joints for next-generation power semiconductor modules", pp. 203–210, Copyright (2017), with permission from Elsevier.]

Nanoparticle/solder hybrid joining technologies have emerged as an innovative way to maximize the advantages of both nanoparticle sintering and conventional soldering for WBG power semiconductor applications. Satoh et al. [148] demonstrated the feasibility of Cu nanoparticle/Bi–Sn solder hybrid joints that had exceptional bonding strength and thermal stability. As shown in Fig. 26a, this hybrid approach involved a two-step firing process. In the first step (200 °C), the Bi–Sn eutectic solder melted and wet the interfaces, and the Cu nanoparticles remained solid. In the second step (350 °C), the Cu nanoparticles sintered and formed Cu–Sn IMCs by reacting with the Sn component, leaving Bi as a separate phase. The resulting microstructure (Fig. 26b) contains a complex arrangement of metallic Cu, Cu–Sn compounds, fine Bi species, and an interfacial Bi-enriched layer.

The excellent performance of these hybrid joints can be attributed to several synergistic effects. During the first firing step, the liquid Bi–Sn phase improved interfacial wetting and facilitated solid–liquid reactions. According to Satoh's findings [148], the Sn component reacted with Cu nanoparticles to form Cu_6_Sn_5_ and Cu_3_Sn IMCs through the reaction:

1 $$\frac{5\text{a}}{6}\text{Cu}(s)+{\text{Bi}}_{\left(1-a\right)}{\text{Sn}}_{\left(a\right)}(l)\to a{\text{Cu}}_{6}{\text{Sn}}_{5}(s)+(1-a)\text{Bi}(s) (410 K< T< 538 K)$$

where *T* is temperature, and $a$ is an arbitrary value < 1,

Concurrently, the remaining Bi created a network that promoted the liquid-phase sintering of Cu nanoparticles through the following eutectic reaction:

2 $$\text{Bi}\left(s\right)+ b\text{Cu}\left(s\right) \left(T< 538 K\right)\leftrightarrow {\text{BiCu}}_{b}\left(l\right) (T> 538 K)$$

where, *b* is an arbitrary value less than the solubility limit of Cu in liquid phase Bi at *T*.

This liquid-phase sintering mechanism significantly enhances densification compared with conventional solid-state sintering, creating robust interconnections while maintaining high-temperature stability through the formation of Cu–Sn IMCs with melting points exceeding 600 °C. Figure 26c clearly demonstrates that the hybrid joints exhibited substantially higher bonding strength than conventional nanoparticle joints, particularly at lower pressures. Whereas Cu nanoparticle joints require high pressure for adequate bonding, the hybrid approach maintained excellent strength (> 30 MPa) at pressures as low as 3 kPa. This reduced pressure dependency is particularly valuable for large area die attachment applications in which uniform pressure applications are challenging. Furthermore, these hybrid joints showed heat resistance superior to that of conventional Pb–Sn solder. The joints maintained higher bonding strength than Pb–10wt.% Sn solder up to 225 °C. However, some strength decrease was observed above 100 °C due to softening of the Bi-rich interfacial layer. These characteristics make hybrid nanoparticle/solder joints particularly promising for WBG semiconductor applications that require operation at elevated temperatures.

#### Dendritic nanostructures

**Fig. 27:**
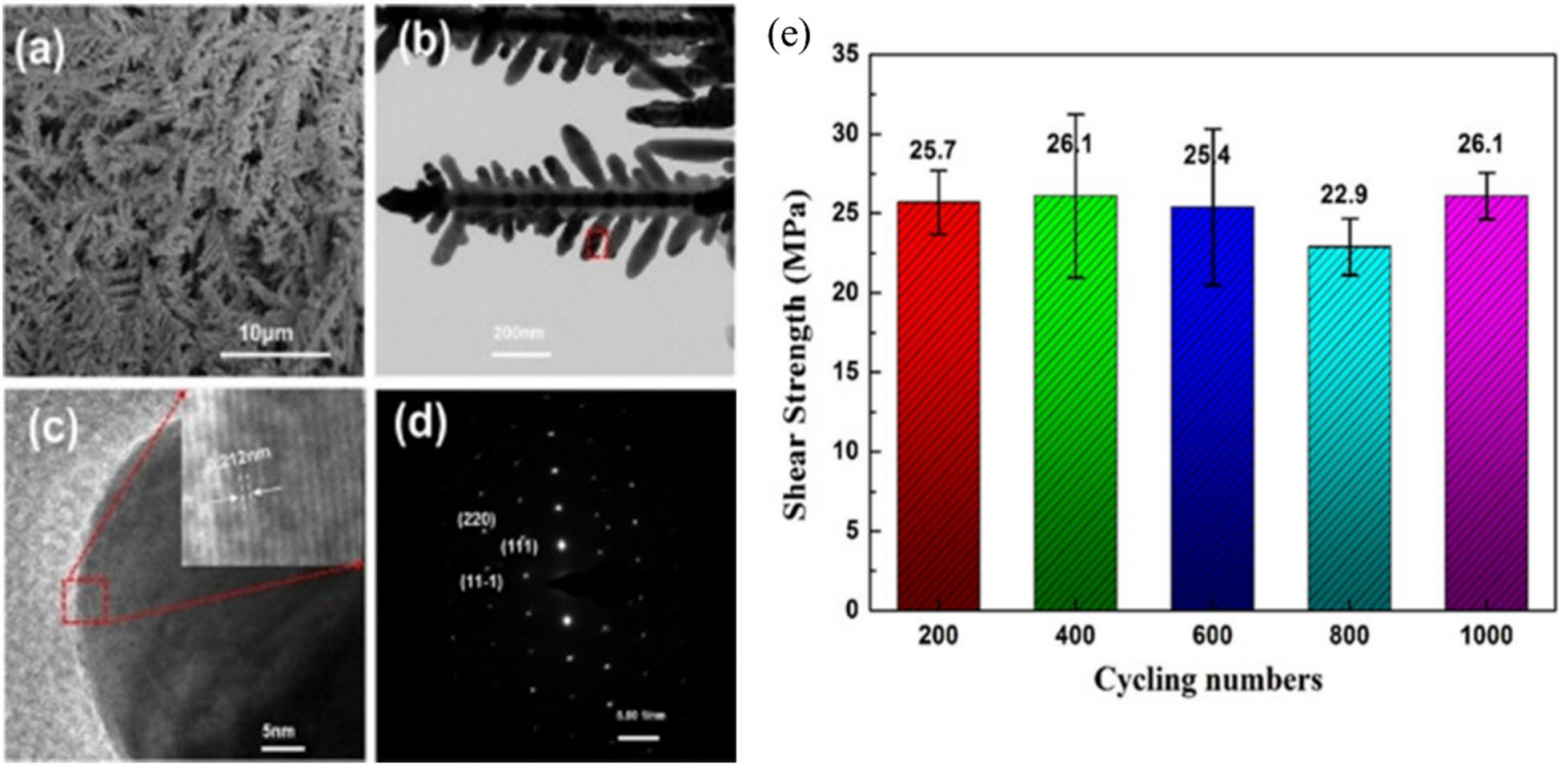
Surface morphological analysis of prepared silver dendrite structures: **a** Low magnified SEM characterization, **b** TEM image of individual silver dendrite structure, **c** High-magnified TEM image and its **d** Corresponding SAED image of dendritic silver, and **e** Shear strength of dendritic Ag–based paste after sintering at 300°C under 10 MPa and subsequently undergoing thermal cycling from − 55 to 125 °C. [Source [149]: J. Fan et al., "Synergistic size and shape effect of dendritic silver nanostructures for low-temperature sintering of paste as die attach materials", Journal of Materials Science: Materials in Electronics, Vol. 32(1), pp. 323-336, 2021. © 2021 Springer Nature. Reproduced with permission from SNCSC.]

Fan et al. [149] investigated the synthesis and application of dendritic silver nanostructures for low-temperature sintering as die attach materials. As shown in Fig. 27a, these hierarchical nanostructures consisted of a central trunk (5–10 μm) and numerous primary branches (approximately 200 nm) that created a complex three-dimensional architecture. The dendritic structure, as confirmed by TEM imaging (Fig. 27b), exhibited a crystalline face-centered structure with well-defined (111), (220), and (11-1) lattice planes visible in the corresponding selected area electron diffraction pattern (Fig. 27c, d).

Unlike spherical nanoparticles that have limited contact points during sintering, dendritic silver offers a distinct advantage. The branched morphology creates extensive interconnection networks with minimal applied pressure. This structure facilitates densification at relatively low sintering temperatures (200–300 °C). Consequently, excellent mechanical performance is achieved. The authors reported that dendritic Ag-based pastes sintered at 300 °C under 10 MPa pressure achieved shear strengths of approximately 28 MPa, significantly higher than comparable silver microspheres (9 MPa) and micro-flakes (11 MPa) in identical conditions.

Most impressively, these dendritic silver joints demonstrated exceptional thermal cycling reliability. As shown in Fig. 27e, samples maintained nearly consistent shear strength values of 25–26 MPa even after 1000 thermal cycles between − 55 and 125 °C. This remarkable stability can be attributed to the ability of the hierarchical structure to accommodate thermomechanical stresses through its complex branched network. These joints also demonstrated excellent thermal and electrical properties. Thermal conductivity reached nearly 79 W m^−1^ K^−1^ at 300 °C sintering temperature. Furthermore, electrical resistivity improved significantly to 9.85 μΩ cm.

The enhanced performance of dendritic structures stems from two key mechanisms: (1) increased contact area between interlaced nanoscale primary branches, which provides extensive sintering interfaces; and (2) the unique ability of the hierarchical structure to distribute thermomechanical stresses during temperature cycling. The micro-sized trunks provide mechanical stability, and the nanoscale branches facilitate low temperature sintering through their high surface energy and enhanced diffusion kinetics.

These results demonstrate that morphology engineering of silver nanostructures is a promising pathway for developing advanced die attach materials that can withstand the extreme operating conditions of WBG power semiconductor devices while maintaining electrical and thermal performance throughout the device lifetime.

#### Nanoporous metal films

Nanoporous metal films have been studied as promising candidates for die-attach applications in WBG power semiconductor devices. These materials have three-dimensional interconnected network of nanoscale ligaments and pores. The distinctive nanostructure of these materials leads to enhanced sintering behavior at relatively low temperatures (200–300 °C) and allows them to maintain thermal stability at operating temperatures above 250 °C.

Nanoporous metals are typically synthesized through dealloying, which involves the selective dissolution of one or more elements from a precursor alloy, leaving behind a sponge-like structure. Nishikawa et al. [150] demonstrated Au nanoporous bonding with exceptional high-temperature reliability, maintaining shear strengths above 25 MPa after isothermal aging at 250 °C for 1000 h. Similarly, Kim [151] explored Ag nanoporous films for high-temperature die-attach applications, showing that the microstructure and mechanical properties could be optimized at bonding temperatures of 300–350 °C. Most recently, Mohan et al. [152] synthesized nanoporous Cu films through electrochemical dealloying of Cu–Si, which achieved strong metallurgical bonding to bulk Cu at temperatures as low as 200–250 °C and moderate pressure (6–9 MPa). Unlike nanoparticle pastes that contain organic additives, nanoporous metal films are organic-free, minimizing the risk of voiding and associated reliability concerns [151].

Table 3 provides a comprehensive comparison of key performance metrics across the various nanocomposite die attach materials reviewed in Sects. 2 and 3. This table systematically compares processing conditions, mechanical properties, thermal properties, electrical properties, porosity, and reliability test results for both nanocomposite solders and nano-sintering materials. The comparative analysis reveals several important trends across material categories. Nanocomposite solders generally exhibit higher processing temperatures (> 240 °C) and demonstrate good shear strength while maintaining relatively lower thermal conductivities compared to sintered alternatives. In contrast, nano-sintering materials can be processed at moderate temperatures (~ 200 °C) and demonstrate superior thermal conductivity with enhanced high-temperature stability but often exhibit higher porosity levels. Advanced processing technologies such as TLPS and core–shell systems are specifically designed to optimize particular performance aspects, effectively combining the processing advantages of conventional solders with the enhanced high-temperature capabilities that approach those of sintered materials.

**Table 3:** Comprehensive performance comparison of nanocomposite die attach materials for WBG power semiconductor packaging

Material system	Category	Processing conditions	Mechanical properties	Microstructural properties	Thermal properties	Electrical properties	Porosity (%)	Test	References
*Nanocomposite solders*									
SAC305	SAC alloy	Reflow: 240 °C	Hardness: 0.159 GPa	IMC thickness: ~ 6.0 μm	N.A	N.A	N.A	N.A	[58]
SAC305–0.5 wt.% NiO	Metal Oxide Enhanced	Reflow: 240 °C	Hardness: ↑54.1% vs pure SAC305	IMC thickness: ~ 4.3 μm	N.A	N.A	N.A	N.A	[58]
SAC305–1.5 wt.% NiO	Metal Oxide Enhanced	Reflow: 240 °C	Hardness: ↑80.5% vs pure SAC305	IMC thickness: ~ 2.6 μm	N.A	N.A	N.A	N.A	[58]
SAC305–2.5 wt.% NiO	Metal Oxide Enhanced	Reflow: 240 °C	Hardness: ↑91.8% vs pure SAC305	IMC thickness: ~ 2.4 μm	N.A	N.A	N.A	N.A	[58]
SAC305	SAC alloy	Reflow: 250 °C Dwell time: 3600 s	N.A	IMC thickness: 13.15 μm IMC grain size: 11.07 μm	N.A	N.A	N.A	N.A	[63]
SAC305–0.1 wt.% TiO_2_	Metal Oxide Enhanced	Reflow: 250 °C Dwell time: 3600 s	N.A	IMC thickness: 10.33 μm IMC grain size: 9.91 μm	N.A	N.A	N.A	N.A	[63]
SAC105	SAC alloy	Reflow: 260 °C	Shear strength: (Before TC) ~ 62 MPa (After TC) 38.65 MPa	IMC thickness: (Before TC) ~ 2.5 μm (After TC) ~ 3.7 μm	CTE: 29.0 × 10^−6^/ °C	N.A	N.A	Thermal cycling test (− 55 to 125 °C for 1000 cycles)	[79]
SAC105–6 nm TiO_2_	Metal Oxide Enhanced	Reflow: 260 °C	Shear strength: (Before TC) ~ 63 MPa, (After TC) 50.33 MPa	IMC thickness: (Before TC) ~ 2.2 μm (After TC) ~ 2.89 μm	CTE: 24.6 × 10^−6^/ °C	N.A	N.A	Thermal cycling test (− 55 to 125 °C for 1000 cycles)	[79]
SAC105–20 nm TiO_2_	Metal Oxide Enhanced	Reflow: 260 °C	Shear strength: (Before TC) ~ 61 MPa, (After TC) 44.44 MPa)	IMC thickness: (Before TC) ~ 2.3 μm (After TC) ~ 3.23 μm	CTE: 26.0 × 10^−6^/ °C	N.A	N.A	Thermal cycling test (− 55 to 125 °C for 1000 cycles)	[79]
SAC	SAC alloy	Reflow: 250 °C Reaction time: 30 min	Elastic modulus: ~ 51 MPa Shear modulus: ~ 19 MPa Shear strength: 33.2 MPa	IMC thickness: (Cu substrate) 9.2 μm (Au/Ni-plated Cu substrate) 4.8 μm	N.A	N.A	N.A	N.A	[80]
SAC–1 wt.% Al_2_O_3_	Metal Oxide Enhanced	Reflow: 250 °C Reaction time: 30 min	Elastic modulus: ~ 59 MPa Shear modulus: ~ 22 MPa Shear strength: 40 MPa	IMC thickness: (Cu substrate) 7.9 μm (Au/Ni-plated Cu substrate) 3.2 μm	N.A	N.A	N.A	N.A	[80]
Sn–3.5Ag	SAC alloy	Reflow: 250 °C Total time: ~ 20 min	N.A	IMC thickness: 5.6 μm	N.A	N.A	N.A	Thermal aging (180 °C for 312 h)	[81]
Sn–3.5Ag–0.2 wt.% ZrO_2_	Metal Oxide Enhanced	Reflow: 250 °C Total time: ~ 20 min	N.A	IMC thickness reduction up to ~ 22%	N.A	N.A	N.A	Thermal aging (180 °C for 312 h)	[81]
Sn–3.5Ag–1.0 wt.% ZrO_2_	Metal Oxide Enhanced	Reflow: 250 °C Total time: ~ 20 min	N.A	IMC thickness reduction up to ~ 5%	N.A	N.A	N.A	Thermal aging (180 °C for 312 h)	[81]
SAC305	SAC alloy	Reflow: 250 °C	Shear strength: (1 reflow cycle): 38 MPa (16 reflow cycle): 31.6 MPa (After 40 days aging time): 28.8 MPa	IMC thickness(1 reflow cycle): 2.1 μm β-Sn grain size: 10–15 μm	N.A	N.A	N.A	Multiples reflow (1–16 cycles, 250 °C ) Thermal aging (150 °C for 10–40 days)	[74]
SAC305–0.5 wt.% SrTiO_3_	Metal Oxide Enhanced	Reflow: 250 °C	Shear strength: (1 reflow cycle): 39.1 MPa (16 reflow cycle): 35.3 MPa (After 40 days aging time): 33.5 MPa	IMC thickness(1 reflow cycle): 1.7 μm β-Sn grain size: 7–10 μm	N.A	N.A	N.A	Multiples reflow (1–16 cycles, 250 °C ) Thermal aging (150 °C for 10–40 days)	[74]
SAC105	SAC alloy	Reflow: 260 °C Dwell time: 50 s	Ultimate tensile strength: 27.9 MPa Elongation: 21.6%	IMC thickness: 0.9 μm β-Sn grain size: 21.71 μm	CTE: 25.0 × 10^−6^/K	N.A	N.A	Thermal aging (150 °C for 1008 h)	[82]
SAC105–0.3 wt.% NiO/ZrO_2_	Metal Oxide Enhanced	Reflow: 260 °C Dwell time: 50 s	Ultimate tensile strength: (As-cast) 34.8 MPa (1008 h aging) 19.6 MPa Elongation: (As-cast) 27.9% (1008 h aging) 29.7%	IMC thickness: 0.9 μm β-Sn grain size: 12.48 μm	N.A	N.A	N.A	Thermal aging (150 °C for 1008 h)	[82]
SAC105–0.3 wt.% ZrO_2_	Metal Oxide Enhanced	Reflow: 260 °C Dwell time: 50 s	Ultimate tensile strength: 29.4 MPa Elongation: 16.5%	N.A	N.A	N.A	N.A	N.A	[82]
Sn95.5Ag3.8Cu0.7	SAC alloy	Reflow with 200 kPa compressive force	Elastic modulus: 23.4 ± 2.3 GPa	N.A	Total thermal interface resistance: 1.6–2.8 K mm^2^ W^−1^ Bulk thermal conductivity: 64 W m^−1^ K^−1^	N.A	N.A	N.A	[36]
SMNPC (solder matrix nano polymer composite)	Nanocomposite solder	Reflow with 200 kPa compressive force	Elastic modulus: 8.1 ± 1.1 GPa	N.A	Total thermal interface resistance: 2.2–3.7 K mm^2^ W^−1^ Through-plane thermal conductivity: ~ 22 W m^−1^ K^−1^ In-plane thermal conductivity: ~ 42 W m^−1^ K^−1^	N.A	N.A	Thermal cycling test (− 40 to 115 °C for 1000 cycles) < 20% resistance change in thermal resistance during thermal cycling test	[36]
Sn–3.5Ag–0.7Cu	SAC alloy	N.A	Melting temperature: 219.22 °C Hardness(292 K): ~ 0.042 GPa Elastic modulus(292 K): ~ 35 GPa	Average grain size: 2.81 μm	N.A	N.A	N.A	Nanoindentation testing for creep resistance evaluation Activation energy: 36.0 kJ/mole	[83]
SAC/Ni-coated(0.1 wt.%) MW-CNTs	Nanocomposite solder	N.A	Melting temperature: 221.34 °C Hardness(292 K): ~ 0.087 GPa Elastic modulus(292 K): ~ 41 GPa	Average grain size: 2.64 μm	N.A	N.A	N.A	Nanoindentation testing for creep resistance evaluation Activation energy: 48.4 kJ/mole	[83]
*Nano-sintering materials*									
AgNP	AgNP paste	Pressure-less bonding at 200 °C for 20 min	Shear strength: 60 MPa	Grain size: 21 nm	Thermal conductivity: 229 W m^−1^ K^−1^	N.A	27%	N.A	[131]
Ag (pressure-assisted)	Ag paste	Pressure-less bonding at 300 °C for 1 h on DBC substrate with Ni/Ag metallization	Shear strength (Initial) > 15.3 MPa (24 h aged at 300 °C) > 15.3 MPa	N.A	N.A	N.A	Pore size increased with aging time, but pore precentage remained constant	Thermal cycling test (− 55 to 300 °C) < 300 cycles endurance Thermal aging (300 °C) > 2000 h endurance	[132]
Ag (pressure-less)	Ag paste	Pressure-assisted bonding (7.6 MPa) at 300 °C for 1 min on DBC substrate with Ni/Ag metallization	Shear strength: (Initial) > 15.3 MPa (24 h aged at 300 °C) > 15.3 MPa	N.A	N.A	N.A	Pore size increased with aging time, but pore precentage remained constant	Thermal cycling test (− 55 to 300 °C) < 750 cycles endurance Thermal aging (300 °C) > 2000 h endurance	[132]
AgNP	AgNP paste	Sintering 200 °C for 20 min after 10 s O_2_ plasma activation (Pressure-less)	Shear strength: > 22.5 MPa	N.A	Thermal conductivity: > 25 W m^−1^ K^−1^	N.A	~ 10%	N.A	[135]
AgNP/20 wt.% NW	Composite paste	Bonding temperature: 200 °C without pressure	Shear strength: 16.4 ± 0.4 MPa (vs pure AgNP: 13.9 ± 0.5 MPa)	N.A	N.A	N.A	The strength decreased considerably when 30% Ag NWs were used due to the porous structure produced in the joint	N.A	[136]
CuNP	Bimodal Cu particle paste (80 nm/300 nm = 3:7)	Pressure-assisted bonding (20 MPa) at 250 °C for 5 min	Shear strength: 102.46 MPa (sintered N_2_ atmosphere)	N.A	Thermal conductivity: 284 W m^−1^ K^−1^	N.A	11.78%	N.A	[137]
Cu + Ag composite	Composite paste	Pressure-assisted bonding (2 MPa) at 250 °C , Ar-H_2_ (5% H_2_) atmosphere	Shear strength: (Cu3Ag1) 22.8 MPa (Cu1Ag3) 32.6 MPa	N.A	N.A	Electrical resistivity: (Cu3Ag1) 26.33 µΩ cm (Cu1Ag3) 8.74 µΩ cm	(Cu3Ag1) 7.37% (Cu1Ag3) 1.92%	N.A	[138]
Ag@Si20%	Composite paste	Pressure-less sintering at 250 °C for 30 min in air	Shear strength: 33.99 MPa	N.A	CTE: 11.08 ppm/K (vs 16.03 for pure Ag)	N.A	N.A	Thermal cycling test (− 50 to 250 °C for 1000 cycles) Strength retention: 64.93%	[139]
**Advanced processing technologies**									
CuNP + Sn-58Bi + PVP (PVP molecular weight: 360,000)	TLPS	Bonding temperature: 220 °C without pressure in H_2_ Bonding time: 60 min	Shear strength: 8.9 MPa Fracture energy: ~ 10 mJ	N.A	N.A	~ 6 × 10^−6^ Ω cm	N.A	high-temperature storage tests (200 °C for 1000 h) Bonding strength and fracture energy decreased drastically with aging times of 500 h	[88]
Cu particle + SAC305 + polyimide resin	TLPS	Preheated at 100 °C for 60 min and bonded at 250 °C for 1 min in a reflow furnace (No pressure, N_2_ atmosphere)	Shear strength: ~ 12 MPa	N.A	Thermal conductivity: 25.9 W m^−1^ K^−1^	N.A	N.A	Thermal cycling test (− 55 to 175 °C for 1200 cycles) The unbonded area of the TLPS joints did not increase during 1200 cycles)	[89]
Cu@Sn	Core–shell structure	Reflow soldering at 250 °C for 8–40 min	Shear strength: (20 min reflow) 29.35 MPa at 400 °C (40 min reflow) 18.78 MPa at 500 °C	IMC thickness: 1–2 μm	N.A	N.A	several voids were found in the bondline	Thermal shock test (− 55 to 200 °C for 500 cycles) No large voids or gaps were observed throughout the large bonding area in the bonded IGBTs samples both before and after thermal cycling	[89]
CuNP + Ni–Sn (47 wt.% sn)	Nanoparticle/solder hybrid joints	The first and second firing steps were performed at 473 K for 10 min and 623 K for 5 min, respectively (H_2_ atmosphere, 3–500 kPa pressure)	Bonding strength: (298 K) ~ 37 MPa (498 K) ~ 8 MPa	N.A	N.A	N.A	N.A	High temperature shear test (298–498 K)	[148]
Dendritic Ag nanostructure (80 wt.% Ag paste)	Dendritic nanostructures	Sintered under 10 MPa pressure for 30 min with various temperatures (150, 200, 250, 300 °C)	Shear strength: 3–28 MPa with increase in sintering temperature	N.A	Thermal conductivity: 2.61, 29.78, 63.81, and 78.89 W m^−1^ K^−1^ for 150, 200, 250 and 300 °C , respectively	4.01 × 10^–4^ Ω cm at 100 °C sintering temperature 9.85 × 10^–6^ Ω cm at 300 °C sintering temperature	N.A	Thermal cycling test (− 55° to 125 °C for 1000 cycles) The average shear strength was 25.6 MPa, 26.1 MPa, 25.4 MPa, 22.9 MPa, and 26.1 MPa after 200, 400, 600, 800, and 1000 thermal cycles, respectively (sintering temperature 300 °C sample)	[143]
Au nanoporous	Nanoporous metal films	Pressure-assisted (20 MPa) bonding at 250–350 °C for 30 min in N_2_ atmosphere	Shear strength:12.5–25 MPa with increase in bonding temperature (dealloying time of 4 h sample)	The dealloying for 4 h results in ligaments with an average size of 9.3 ± 1.7 nm	N.A	N.A	N.A	Thermal storage test (250 °C for 1000 h) Initial strength (32 MPa) was decreased to 27 MPa after 1000 h (dealloying time of 1 h sample)	[144]
Ag nanoporous	Nanoporous metal films	Pressure-assisted (20 MPa) bonding at 200–400 °C (soak time: 30 min) in air and N_2_ atmosphere	Shear strength: (Air, 200 °C) 14.4 MPa (N_2_, 200 °C) 16.2 MPa (Air, 400 °C) 22 MPa (N_2_, 400 °C) 27 MPa	N.A	N.A	N.A	(200 °C) 22.5% (400 °C) 13%	N.A	[145]

N.A: Not Available

## Reliability of die attach materials for WBG power semiconductors

The reliability of die attach materials in WBG semiconductor packages is a critical concern for next-generation power electronic systems. These materials must maintain mechanical integrity, thermal performance, and electrical functionality under the increasingly extreme operating conditions characteristic of WBG devices. Compared with conventional silicon-based semiconductors, which typically operate at junction temperatures of 125–150 °C, WBG semiconductors function at substantially higher temperatures, often exceeding 200–300 °C. Additionally, these devices experience rapid thermal cycling, high power densities, and rapid switching speeds throughout their operational lifetime. The unique stress encountered in WBG applications has necessitated the development of specialized reliability assessment methodologies and failure mode analyses that extend beyond traditional semiconductor packaging evaluation techniques.

In this section, we systematically analyze the reliability of nano-enabled die attach materials for WBG applications at multiple levels: (1) intrinsic material level, focusing on fundamental material degradation mechanisms; (2) package level, examining the response of die attach interfaces to thermal and electrical stress; and (3) module-field level, evaluating long-term performance in application-specific conditions. This approach provides comprehensive insight into reliability challenges and enables the development of strategies to enhance the performance and longevity of WBG power semiconductor packages.

### Intrinsic level reliability

Intrinsic level reliability focuses on fundamental material degradation mechanisms that occur within the nano-enabled die attach materials themselves. At this level, the microstructural stability and inherent material properties play decisive roles in determining long-term reliability. Several key intrinsic reliability factors have been identified through extensive research in recent years.

#### Microstructural evolution

The microstructural evolution of nanoparticle-based die attach materials under thermal stress is a primary concern for intrinsic reliability. Unlike conventional solder materials that experience IMC growth during thermal aging, nano-sintered materials undergo distinctly different degradation mechanisms. For silver nanoparticle–based systems, grain coarsening has been identified as the predominant microstructural change during high-temperature exposure. Zhang et al. [153] conducted a systematic investigation of nano-silver sintered die attach for SiC power semiconductor devices at temperatures up to 350 °C. Their findings revealed a progressive transformation of the initially fine nanocrystalline structure to a coarser microstructure with prolonged high-temperature exposure. The die attach interface experienced significant microstructural changes during thermal aging. The initially homogeneous nano-silver structure with grain sizes of 100–200 nm evolved to a coarser structure with grains exceeding 1–2 μm after 800 h of aging at 350 °C. This grain coarsening process typically follows a classical Oswald ripening mechanism, where larger grains grow at the expense of smaller grains to minimize the total interfacial energy of the system. The coarsening rate exhibits temperature-dependent behavior following an Arrhenius relationship, with activation energies typically ranging from 0.5 to 1.2 eV depending on the specific nanoparticle composition and processing conditions [153, 154].

For nanocomposite solder systems, the microstructural evolution is considerably more complex due to the interaction between the solder matrix and dispersed nanoparticles. Research by Chellvarajoo et al. [58] on NiO-reinforced SAC305 solder revealed that properly dispersed nanoparticles effectively slowed grain coarsening by pinning grain boundaries. The presence of thermally stable ceramic nanoparticles at grain boundaries created Zener pinning effects that significantly reduced grain boundary mobility and suppressed microstructural coarsening. This microstructural stabilization maintained the mechanical performance of solder joints during thermal aging.

#### Porosity evolution

Porosity is an inherent characteristic of sintered nanoparticle die attach materials that significantly affect their reliability. Unlike solders that form dense, fully consolidated joints, nano-sintered materials retain a certain degree of porosity even under optimized processing conditions. Although the initial porosity is determined primarily by processing parameters such as sintering temperature, pressure, and duration, the evolution of porosity during thermal cycling has profound implications for long-term reliability.

Wang et al. [131] investigated the relationship between the organic shell thickness on silver nanoparticles and the resulting porosity after sintering. Their findings demonstrate that thinner organic shells facilitated more efficient densification, resulting in lower initial porosity (approximately 27% versus 40% for thicker shells). That study further revealed that porosity evolution during thermal aging followed distinct patterns depending on the initial microstructure. Samples with lower initial porosity exhibited greater microstructural stability during thermal aging, with minimal changes in pore size and distribution after 1000 h at 150 °C. Conversely, samples with higher initial porosity showed accelerated pore coarsening and coalescence during identical aging conditions. This resulted in significantly higher porosity increases of 15–20%, which led to corresponding reductions in both thermal and mechanical performance.

**Fig. 28:**
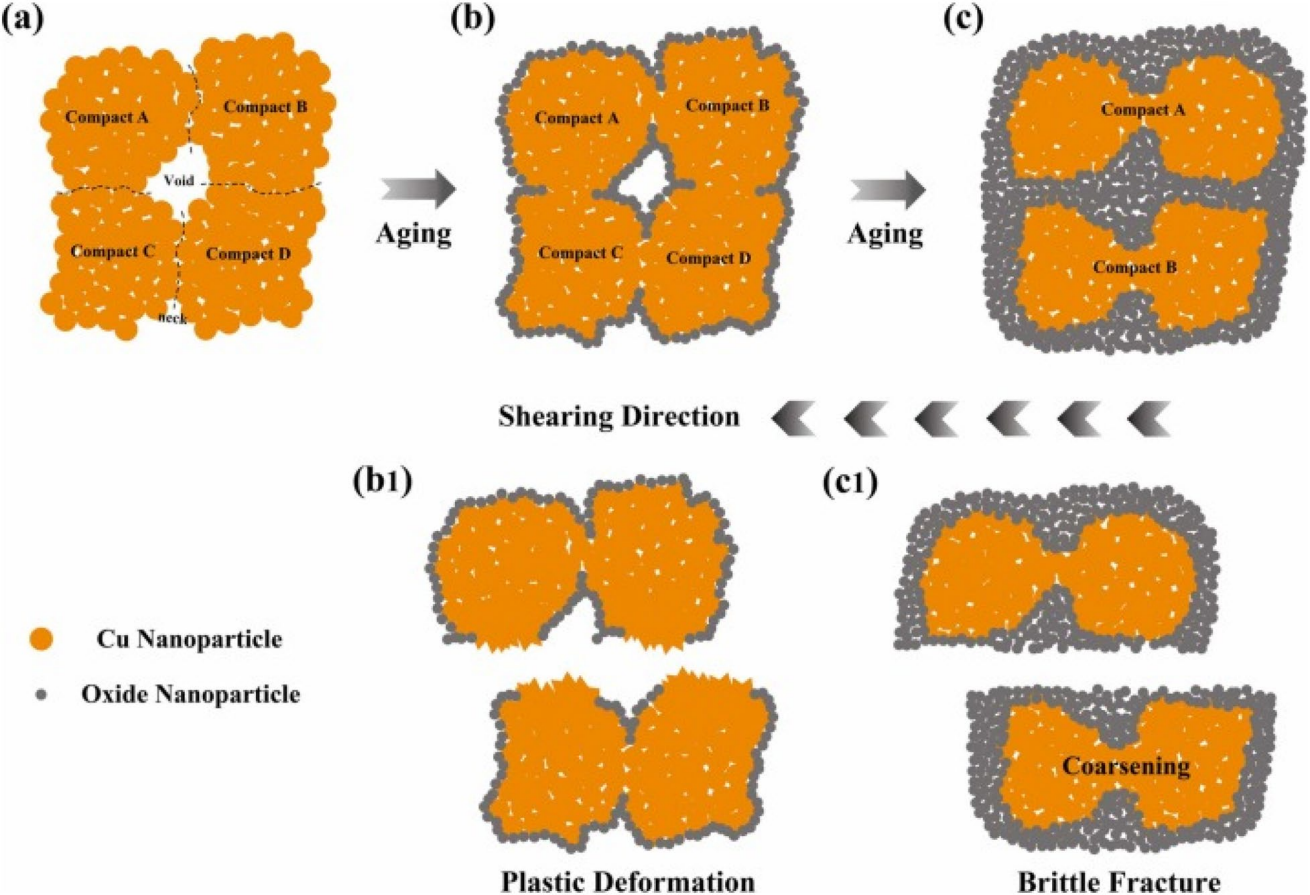
Schematic illustration of the coarsening process in a sintered Cu structure. [Source [121]: Reprinted from Corrosion Science, Vol. 209, Y. Zuo, A. Robador, M. Wickham, and S.H. Mannan, "Unraveling the complex oxidation effect in sintered Cu nanoparticle interconnects during high temperature aging", 110713, Copyright (2022), with permission from Elsevier.]

For copper nanoparticle–based die attach materials, porosity evolution is even more important due to oxidation effects. Zuo et al. [121] conducted a comprehensive analysis of sintered copper nanoparticle interconnects during high-temperature aging up to 250 °C. Their research revealed that nanopores in sintered copper die attach materials serve as preferential oxidation sites due to their high surface energy and accessibility to oxygen (Fig. 28). These initial pores allow oxygen to enter, causing oxidation. The oxidation process creates volume expansion, which forms more pores. This creates a harmful cycle that continuously accelerates material degradation. That study highlighted the critical importance of controlling both initial porosity and oxygen access to enhance the reliability of copper nanoparticle–based die attach materials.

#### Interface stability

The stability of interfaces between the die attach material and the metallization layers on both the die and substrate is another critical aspect of intrinsic reliability. Unlike conventional solder joints where interfacial degradation primarily involves IMC growth, nano-enabled die-attach materials face different interfacial challenges. For silver nanoparticle systems, diffusion-related phenomena at the silver-metallization interface significantly affect long-term reliability.

Zhang et al. [153] compared the interfacial stability of nano-silver sintered joints with different substrate metallization: bare copper, silver-plated copper, and electroless nickel immersion gold (ENIG). Their results demonstrate substantially different interface evolution mechanisms. For silver-plated substrates, excellent interfacial stability was observed with minimal diffusion or reaction products even after 800 h at 350 °C. Conversely, ENIG-metallized substrates exhibited significant interfacial degradation after only 400 h at the same temperature, characterized by void formation and nickel oxide growth at the interface. The study revealed that direct silver-to-silver interfaces provide superior reliability due to the absence of dissimilar material reactions and associated voiding mechanisms.

For nanocomposite solder systems, interfacial stability is predominantly governed by the growth kinetics of IMCs, which can be effectively modulated by nanoparticle additions. Wodak et al. [81] demonstrated that incorporating ZrO_2_ nanoparticles into Sn-3.5Ag solder significantly altered the IMC growth kinetics during thermal aging at 453 K (179.85 °C). The ZrO_2_ nanoparticles transformed the interfacial Cu_6_Sn_5_ morphology from scallop-like structures to more planar configurations, reducing the effective diffusion coefficient from 7.84 × 10^–18^ m^2^ s^−1^ in undoped joints to just 2.89 × 10^–18^ m^2^ s^−1^ in joints containing 1.0 wt.% ZrO_2_. This substantial reduction in the IMC growth rate can improve reliability during thermal cycling by preserving interfacial mechanical properties.

### Package level reliability

Package level reliability focuses on the response of die attach interfaces to the complex stresses experienced within the semiconductor package environment. At this level, the interaction between different package components and associated thermo-mechanical stresses are the predominant reliability factors. Several key package-level reliability aspects have been extensively investigated for nano-enabled die attach materials in WBG applications.

#### Thermal cycling reliability

Thermal cycling is one of the most stringent reliability tests for die attach materials in WBG semiconductor packages. During thermal cycling, the substantial CTE mismatch between the WBG semiconductor die (4.0 ppm K^−1^ for SiC) and substrate materials (17 ppm K^−1^ for copper, 7 ppm K^−1^ for alumina) generates significant thermomechanical stress at the die attach interface [155]. This stress is particularly concentrated at the corners and edges of the die, creating conditions conducive to crack initiation and propagation.

**Fig. 29:**
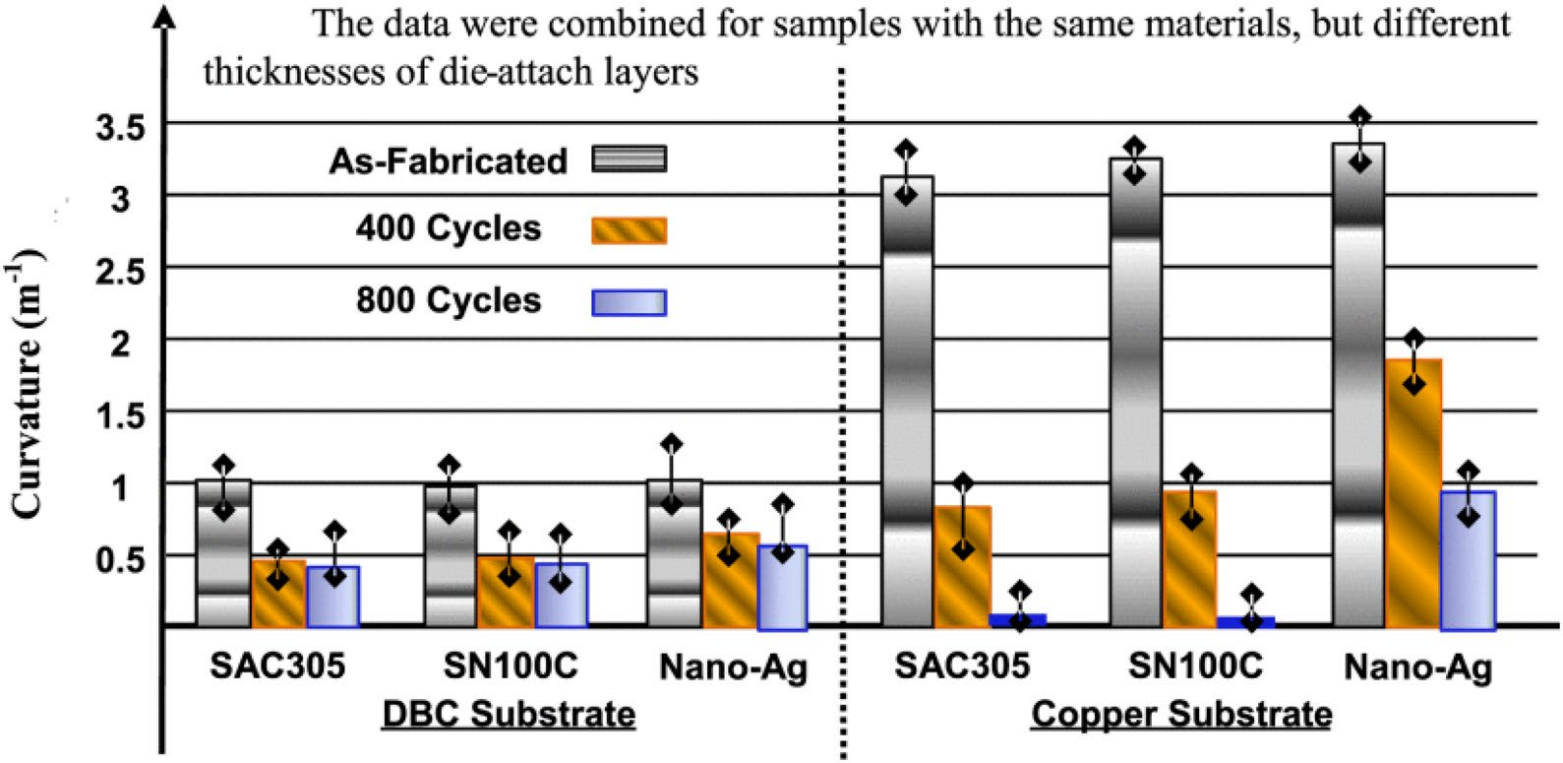
Curvatures decrease with thermal cycling for samples with different substrates and die attach layers [Source [156]: Reprinted with permission from IEEE Transactions on Components, Packaging and Manufacturing Technology. L. Jiang, T.G. Lei, K.D. Ngo, G.-Q. Lu, and S. Luo, "Evaluation of Thermal Cycling Reliability of Sintered Nanosilver Versus Soldered Joints by Curvature Measurement", IEEE Transactions on Components, Packaging and Manufacturing Technology, Vol. 4, No. 5, pp. 751-761, 2014. © 2014 IEEE.]

Jiang et al. [156] conducted an extensive comparison between nano-silver sintered joints and conventional lead-free solders (SN100C and SAC305) during thermal cycling between − 40 and 125 °C. They developed an innovative methodology using curvature measurements as a quantitative indicator of die-attach degradation. After 800 thermal cycles, nano-silver sintered joints on copper substrates maintained 30% of their original curvature with no visible crack formation. In contrast, soldered joints lost more than 95% of their curvature and exhibited significant cracking (Fig. 29). The superior thermal cycling performance of nano-silver joints was attributed to their unique microstructure with distributed porosity that effectively accommodates strain, lower elastic modulus (approximately 10 GPa compared to 30 GPa for solders), and absence of brittle IMCs.

For nanocomposite solders, thermal cycling reliability is significantly enhanced by the incorporation of appropriately selected nanoparticles. Wen et al. [79] demonstrated that adding TiO_2_ nanoparticle to SAC105 solder substantially improved thermal cycling reliability. After 1000 thermal cycles between − 40 and 125 °C, the SAC105–TiO_2_ nanocomposite solder maintained high shear strength (approximately 32 MPa), compared with the rapid degradation observed in unmodified SAC105 solder (below 20 MPa). Microstructural analysis revealed that the TiO_2_ nanoparticles effectively suppressed both β-Sn grain coarsening and interfacial IMC growth during thermal cycling, preserving the mechanical integrity of the solder joint throughout the test duration.

The incorporation of silicon particles into silver sintering pastes is another innovative way to enhance thermal cycling reliability. Liu et al. [139] developed an Ag@Si composite sintering strategy that demonstrated exceptional thermal cycling performance between − 50 and 250 °C. As shown in Fig. 25, the Ag@Si20% composite joints maintained structural integrity with minimal cracking even after 1000 thermal cycles, whereas pure silver joints exhibited extensive crack networks. This remarkable improvement was attributed to CTE modification (from 16.03 ppm K^−1^ for pure Ag to 11.08 ppm K^−1^ for Ag@Si20%), which significantly reduced the thermal expansion mismatch with the SiC die.

#### Power cycling reliability

Whereas thermal cycling evaluates passive thermal stress resistance, power cycling reliability assesses the performance of die attach materials during active electrical operation conditions. Power cycling subjects the package to heat pulses internally generated during device switching, creating temperature gradients and localized heating that can accelerate specific failure mechanisms not captured by thermal cycling alone.

**Fig. 30:**
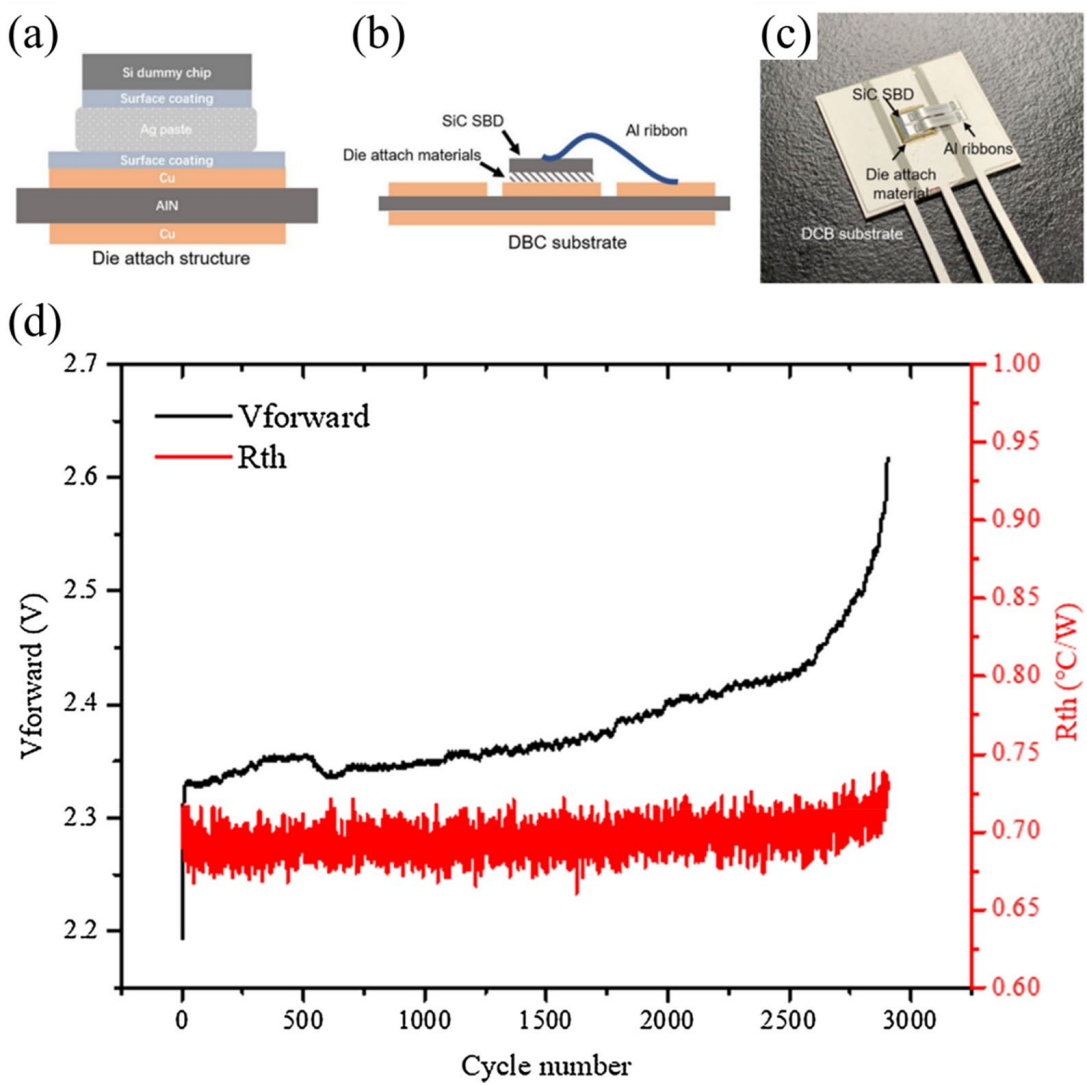
Schematic cross-section of **a** the die attach joint and **b** SiC diode module, **c** Image of the prepared SiC diode module, and **d** Forward voltage and junction-to-case thermal resistance variation during the power cycling test. [Source [157]: Z. Zhang, C. Chen, A. Suetake, M.-C. Hsieh, and K. Suganuma, "Reliability of Ag Sinter-Joining Die Attach Under Harsh Thermal Cycling and Power Cycling Tests", Journal of Electronic Materials, Vol. 50(12), pp. 6597-6606, 2021. © 2021 The Minerals, Metals & Materials Society. Reproduced with permission from SNCSC.]

Zhang et al. [157] evaluated the power cycling reliability of silver-sintered die-attach for SiC diodes (Fig. 30a–c) in extreme conditions (initial junction temperature of 200 °C, temperature swing of 157 °C). The sintered silver die attach material used ball-milled silver flakes containing nano-subgrains, which enabled excellent sinter ability at relatively low temperatures (250 °C) without requiring external pressure. The power cycling results (Fig. 30d) demonstrated exceptional stability, with the sintered joints maintaining consistent performance through 2901 cycles. The junction-to-case thermal resistance remained stable at approximately 0.7 °C W^−1^ for the first 2600 cycles, showing only a minimal 5.3% increase at the final stage of testing. However, during the power cycling test, the forward voltage (V_f_) increased with a significant increment of 13.4% by the end of testing. This evidence suggests that the primary failure mode was ribbon bond lift-off rather than die attach degradation.

The researchers explained the failure progression mechanism as follows: the different thermal expansion coefficients between the semiconductor materials and metallic interconnects generated severe thermal stress during heating and cooling cycles. That stress initially concentrated at the topside ribbon bond, causing micro-cracking and eventual lift-off. The ribbon bond degradation increased the forward voltage drop, which in turn raised the power consumption and junction temperature. That elevated temperature then accelerated the degradation in a self-reinforcing cycle. A computed tomography analysis confirmed the absence of new voids or microcracks in the die-attach layer throughout the power cycling test, demonstrating the exceptional thermal–mechanical stability of the silver-sintered joint under extreme thermal-electrical stresses.

**Fig. 31:**
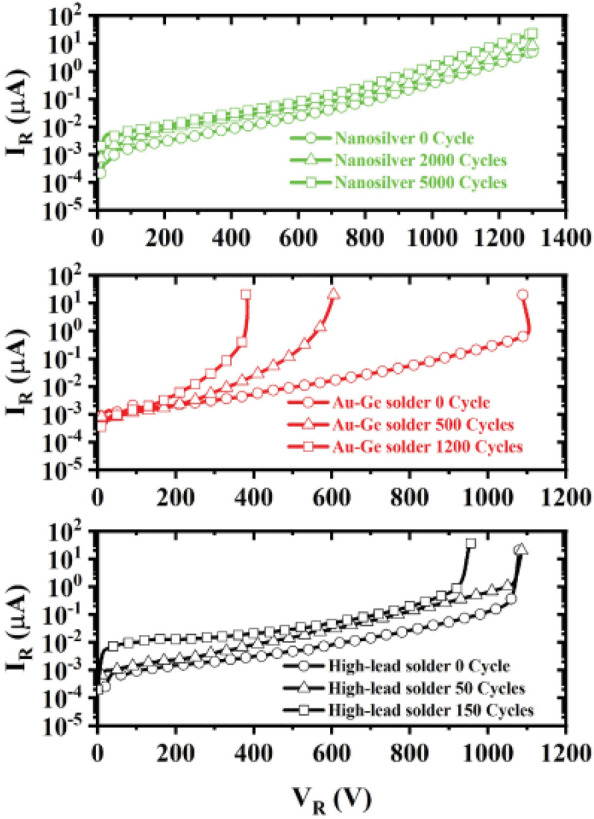
V_R_ and I_R_ variations of SiC JBS devices using three die attach materials at 300°C PC [Source [158]: Reprinted with permission from IEEE Journal of Emerging and Selected Topics in Power Electronics. Z. Hu et al., "Degradation in Electrothermal Characteristics and Failure Mechanism of SiC JBS With Different Die Attach Materials Under 300 °C Power Cycle Stress", IEEE Journal of Emerging and Selected Topics in Power Electronics, Vol. 12, No. 4, pp. 3619-3628, 2024. © 2024 IEEE.]

A more comprehensive comparison study by Hu et al. [158] evaluated SiC junction barrier Schottky (JBS) diodes with three different die-attach materials (high-lead solder, Au–Ge solder, and nano-silver paste) at 300 °C power cycling conditions. As shown in Fig. 31, nano-silver significantly outperformed the conventional solders, enduring 4000 cycles, compared with only 50 cycles for high-lead solder and 1200 cycles for Au–Ge solder. The superior performance of nano-silver was attributed to its unique single-metal structure that prevented the formation of the IMCs and Kirkendall voids that typically develop in conventional solders under power cycling conditions. Additionally, the nanostructured silver layer possessed unique microstructural features, including high-density twin boundaries and refined grain structure. These features enabled better thermal and electrical performance at elevated temperatures. Consequently, the material is ideally suited for WBG device applications in extreme operating environments.

#### High-temperature storage reliability

High-temperature storage testing evaluates the stability of die-attach materials during sustained high-temperature exposure without electrical operation. This test primarily assesses the material degradation mechanisms that occur during isothermal conditions, such as grain coarsening, IMCs growth, and interface reactions.

**Fig. 32:**
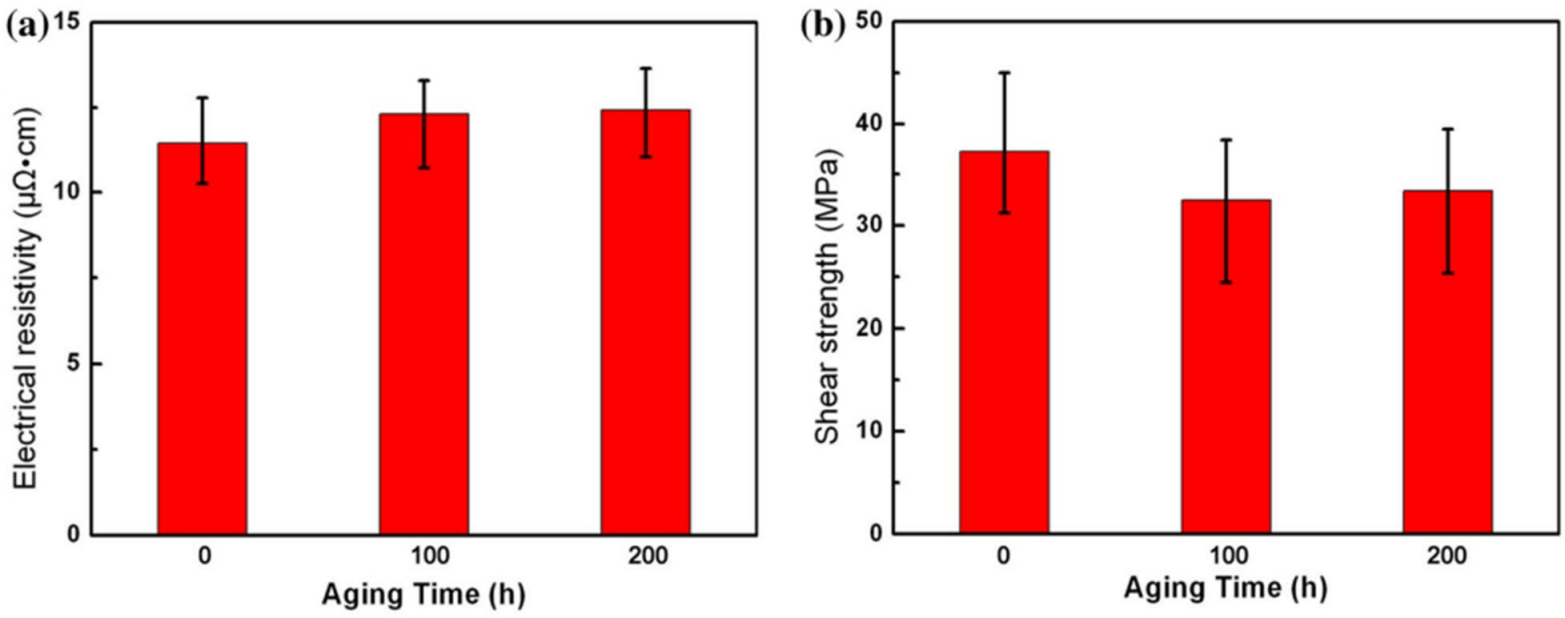
Variation in **a** Electric electrical resistivity for reflowed preform and **b** Shear strengths of the bond line before and after isothermal aging tests at 200°C for 100–200 h [Source [159]: Fuwen Yu et al., "Rapid Formation of Full Intermetallic Bondlines for Die Attachment in High-Temperature Power Devices Based on Micro-sized Sn-Coated Ag Particles", JOM: The Journal of the Minerals, Metals & Materials Society (TMS), Vol. 71(9), 2019, Reproduced with permission from SNCSC.]

Yu et al. [159] developed an innovative die attach approach using micro-sized Sn-coated Ag particles that rapidly formed Ag_3_Sn IMCs bond lines after just 10 min at 250 °C. The resulting joints exhibited excellent high-temperature storage reliability with exceptional thermal stability due to the high melting point (480 °C) of the Ag_3_Sn IMCs. After isothermal aging at 200 °C for 200 h, the shear strength decreased by only 11.7%, maintaining values above 20 MPa. This outstanding thermal stability was attributed to the thermodynamically stable Ag_3_Sn microstructure with fine grains and high-density twin boundaries that effectively resisted coarsening during high-temperature exposure (Fig. 32).

For silver nanoparticle systems, the optimal metallization strategy significantly affects high-temperature storage reliability. Zhang et al. [153] compared various metallization approaches for nanosilver die-attach applications at temperatures up to 350 °C. Their findings revealed that standard ENIG surface finishes failed after only 400 h of high-temperature storage at 350 °C due to nickel oxide formation at the interface. However, incorporating an electroplated silver diffusion barrier extended reliability beyond 800 h in identical conditions. That study demonstrated the critical importance of metallization selection for extreme temperature applications, with silver-to-silver interfaces providing superior reliability for silver nanoparticle-based die-attach materials.

### Module-field level reliability

Module-field level reliability extends the evaluation of die attach materials to system-level performance in application-specific conditions, while considering interactions with other module components and environmental factors. This level of reliability assessment provides the most comprehensive understanding of how nano-enabled die-attach materials will perform in actual WBG power electronic systems throughout their operational lifetime.

#### Electric field effects

The unique electrical characteristics of WBG semiconductors, particularly their high breakdown voltage capabilities (typically exceeding 1200 V, with some devices reaching 10 kV), introduce significant electric field stresses within the package [10]. These intense electric fields (2–3 MV cm^−1^) can accelerate degradation mechanisms in die attach materials, especially at the interfaces where field enhancement occurs due to geometric discontinuities or material transitions.

**Fig. 33:**
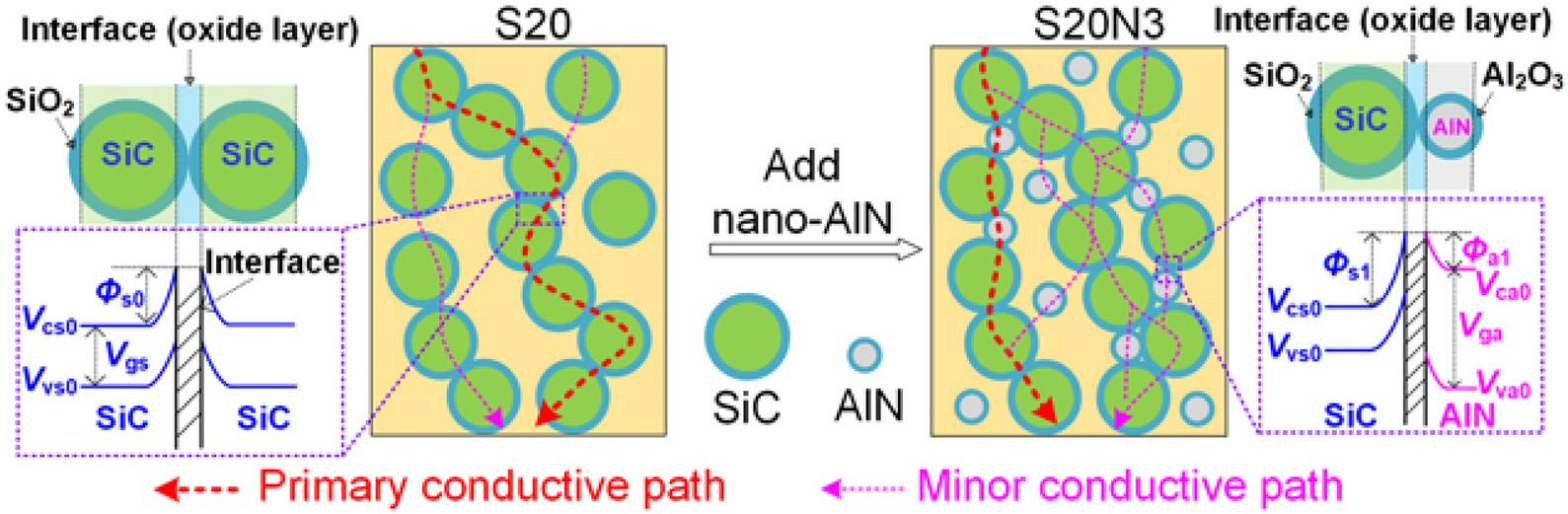
Schematic representing the mechanism for the nonlinear conductive characteristics of S20 and S20N3 [Source [160]: Wang et al. “Enhanced field-dependent conductivity and material properties of nano-AlN/micro-SiC/ silicone elastomer hybrid composites for electric stress mitigation in high-voltage power modules”, Nanotechnology, Vol. 33(47), pp. 475706, 2022, DOI: 10.1088/1361-6528/ac8aa0. © IOP Publishing. Reproduced with permission. All rights reserved.]

Wang et al. [160] investigated electric field effects in WBG module packaging and demonstrated that incorporating nano-AlN fillers into micro-SiC/silicone elastomer composites significantly mitigated electric field stress. As shown in Fig. 33, the primary conductive path in conventional micro-SiC composites (S20) follows a tortuous route through SiC particle contacts. When nano-AlN particles were introduced (S20N3), they create additional minor conductive paths while also establishing higher interface barriers between dissimilar materials.

That study revealed unique mechanistic insights into how these nano-scale additions modify electrical behavior. As illustrated in Fig. 33, interface oxide layers (SiO_2_ and Al_2_O_3_) spontaneously formed on the SiC and AlN particles, creating Schottky-like barriers at particle interfaces. The heterogeneous m-SiC/n-AlN interfaces in S20N3 developed higher barrier heights (Φ_s1_) than the homogeneous m-SiC/m-SiC interfaces (Φ_s0_) in S20 due to the substantial bandgap difference between SiC (2.2 eV) and AlN (6.2 eV). The interface barriers played a crucial role in controlling charge transport through the composite. Higher barrier heights increased the switching electric field threshold. Conversely, these barriers enhanced nonlinear behavior after activation. Notably, S20N3 demonstrated superior nonlinear behavior with a higher nonlinearity coefficient (α = 6.12 compared with 4.02 for S20) and a higher switching electric field (1.75 kV mm^−1^ compared with 0.93 kV mm^−1^ for S20).

This nonlinear conductivity behavior effectively redistributed electric fields within the module, reducing field concentration at critical interfaces including the die attach region. That study further revealed that the hybrid nano–micro composite achieved superior electric field reduction (82.1% compared with pure silicone elastomer) and exhibited shorter dielectric relaxation times, directly addressing the critical challenge of partial discharge at triple points in high-voltage WBG power module packaging.

For metallized die-attach interfaces, Wang et al. [161] found that electric field enhancements of 1.5–2 times can occur at die-attach edge terminations in medium-voltage SiC modules. These concentrated electric fields accelerate interfacial degradation through mechanisms such as partial discharge, localized heating, and enhanced ion migration. That study demonstrated that optimizing metallization geometries and incorporating field-grading materials at critical interfaces can significantly enhance the electric field withstand capability of die-attach materials, particularly in applications exceeding 3.3 kV where electric field effects become increasingly dominant reliability factors.

## Conclusion

This comprehensive review has analyzed recent advances in nanomaterial-based die attach technologies for WBG power semiconductor packaging. The challenges of WBG semiconductor packaging, including high junction temperature, fast switching speeds, and severe thermomechanical stress, require the development of high-performance die attach materials. Nanocomposite solders incorporating metal oxide and metallic nanoparticles have demonstrated significant improvements in mechanical strength, thermal stability, and reliability through grain refinement, dispersion strengthening, and IMC growth inhibition. Advanced nano-sintering technologies have achieved exceptional thermal conductivity exceeding 200 W m^−1^ K^−1^ and high-temperature stability while enabling low-temperature processing. Novel nanostructured approaches such as core–shell particles, TLPS systems, and dendritic architecture have further expanded the flexibility and reliability of next-generation packaging solutions.

Despite these significant advances, several critical limitations constrain widespread adoption of nanomaterial-based die attach technologies. Processing challenges include high costs and scalability issues of noble metal-based nanomaterials, manufacturing complexity from multi-step synthesis and surface functionalization, and specialized equipment requirements for controlled atmosphere processing. Material performance limitations include inherent porosity in sintered structures that create thermal and electrical barriers, oxidation sensitivity of copper-based nanomaterials, and CTE mismatch between nanomaterials and WBG substrates. Additionally, there are reliability and standardization gaps, including lack of industry testing standards, insufficient long-term field data, and variations affecting reproducibility.

To advance these technologies toward commercial use, future research should focus on several key areas. First, developing low-cost materials by creating copper-based alternatives with better oxidation resistance and combining small amounts of expensive nanomaterials with cheaper conventional materials can reduce costs while maintaining performance. Second, improving manufacturing processes through in-situ monitoring technology, 3D printing technology, and faster sintering methods using microwave or laser heating will enhance production efficiency. Third, optimizing material properties by controlling porosity, creating materials with multiple functions, and developing self-healing capabilities will improve long-term performance. Finally, enhancing reliability through better prediction models, standardized testing methods, and monitoring systems will ensure consistent quality.

The integration of nanomaterials in die attach technologies represents a paradigm shift toward high-performance packaging solutions capable of meeting WBG semiconductor demands. While significant technical challenges remain, rapid progress in nanomaterial synthesis, processing techniques, and reliability understanding provides a clear pathway toward commercial implementation. The continued development of these technologies will be crucial for enabling the full potential of WBG semiconductors in next-generation applications including electric vehicles, renewable energy systems, and high-efficiency power conversion systems.

## Data Availability

The review is based on the published data and sources of data upon which conclusions can be found in the reference list.

## Abbreviations

A: Ampere
Ag: Silver
Ag_3_Sn: Silver–tin intermetallic compound
AgNO_3_: Silver nitrate
AgNP: Silver nanoparticle
AgNW: Silver nanowire
Al_2_O_3_: Aluminum oxide
AlN: Aluminum nitride
AT: Aging test
Au: Gold
Au-Ge: Gold-germanium
BFOM: Baliga’s figure of merit
BHN: Brinell hardness number
Bi: Bismuth
BiNi: Bismuth–Nickel
BLT: Bond line thickness
CNT: Carbon nanotube
CTE: Coefficient of thermal expansion
Cu: Copper
Cu_3_Sn: Copper–Tin intermetallic compound
Cu_6_Sn_5_: Copper–Tin intermetallic compound
DBC: Direct bonded copper
DMAB: Dimethylamine Borane
E_b_: Switching electric field
EBSD: Electron backscatter diffraction
EDS: Energy dispersive spectroscopy
ENIG: Electroless nickel immersion gold
est.: Estimate
eV: Electron volt
GaN: Gallium nitride
h: Hour
HRTEM: High-resolution transmission electron microscopy
HV: Vickers hardness
Hz: Hertz
IGBT: Insulated gate bipolar transistor
IMC: Intermetallic compound
I_R_: Reverse current
JBS: Junction Barrier Schottky
K: Kelvin
KOH: Potassium hydroxide
m-SiC: Mirco-sized silicon carbide
min: Minute
mm: Millimeter
MPa: Megapascal
MV: Megavolt
MW-CNT: Multi-walled carbon nanotube
n-AIN: Nano-sized aluminum nitride
NaBH_4_: Sodium borohydride
Ni: Nickel
NiO: Nickel oxide
nm: Nanometer
NP: Nanoparticle
NW: Nanowire
Pb: Lead
PC: Power cycling
ppm: Parts per million
PVP: Polyvinylpyrrolidone
RoHS: Restriction of hazardous substances
R_th_: Thermal resistance
s: Second
SAC: Sn–Ag–Cu (Tin–Silver–Copper)
SAC305: Sn–3.0Ag–0.5Cu (96.5 wt.% Sn, 3.0 wt.% Ag, 0.5 wt.% Cu)
SAC387: Sn–3.8Ag–0.7Cu (95.5 wt.% Sn, 3.8 wt.% Ag, 0.7 wt.% Cu)
SAC396: Sn–3.9Ag–0.6Cu (95.5 wt.% Sn, 3.9 wt.% Ag, 0.6 wt.% Cu)
SAC105: Sn–1.0Ag–0.5Cu (98.5 wt.% Sn, 1.0 wt.% Ag, 0.5 wt.% Cu)
SAC0705: Sn–0.7Ag–0.5Cu (98.8 wt.% Sn, 0.7 wt.% Ag, 0.5 wt.% Cu)
SAED: Selected area electron diffraction
SAM: Scanning acoustic microscopy
SEM: Scanning electron microscopy
Si: Silicon
SiC: Silicon carbide
SiO_2_: Silicon dioxide
SMNPC: Solder matrix nano polymer composite
Sn: Tin
Sn–58Bi: 58 Wt.% Bi, 42 wt.% Sn (Eutectic composition)
Sn–9Zn: Sn–9wt.%Zn (91 wt.% Sn, 9 wt.% Zn)
Sn–5Sb: Sn–5wt.%Sb (95 wt.% Sn, 5 wt.% Sb)
SrTiO_3_: Strontium titanate
SS: Shear strength
TC: Thermal cycling
TEM: Transmission electron microscopy
TiO_2_: Titanium dioxide
TLPS: Transient liquid phase sintering
TS: Tensile strength
V_f_: Forward voltage
V_R_: Reverse voltage
W: Watt
WBG: Wide bandgap
ZnO: Zinc oxide
ZrO_2_: Zirconium dioxide
°C: Degrees celsius
α: Nonlinearity coefficient
μm: Micrometer
Ω: Ohm
Φ_s_: Schottky Barrier height
